# Health Opportunity Costs: Assessing the Implications of Uncertainty Using Elicitation Methods with Experts

**DOI:** 10.1177/0272989X20916450

**Published:** 2020-05-22

**Authors:** Marta O. Soares, Mark J. Sculpher, Karl Claxton

**Affiliations:** Centre for Health Economics, University of York, York, UK; Centre for Health Economics, University of York, York, UK; Centre for Health Economics and Department of Economics, University of York, York, Yorkshire, UK

**Keywords:** elicitation, experts, health opportunity costs, threshold, uncertainty

## Abstract

Well-established methods of economic evaluation are used in many countries to inform decisions about the funding of new medical interventions. To guide such decisions, it is important to consider what health gains would be expected from the same level of investment elsewhere in the health care system. Recent research in the United Kingdom has evaluated the evidence available and the methods required to estimate the health effects of changes in health care expenditure within the National Health Service. Because of the absence of sufficiently broad-ranging data, assumptions were required in the previously mentioned work to estimate health effects in terms of a broader measure of health (quality-adjusted life-years), which is more relevant for policy. These assumptions constitute important sources of uncertainty. This work presents an application of the structured elicitation of the judgments of key individuals about these uncertain quantities. This article describes the design and conduct of the exercise, including the quantities elicited, the individual (rather than consensus) approach used, how uncertainty in knowledge was elicited (mode and bounds of an 80% credible interval), and methods to generate group estimates. It also reports on a successful application involving 28 clinical experts and 25 individuals with policy responsibilities. Although, as expected, most experts found replying to the questions challenging, they were able to express their beliefs quantitatively. Consistent across the uncertainties elicited, experts’ judgments suggest that the quality-adjusted life-year (QALY) impacts of changes in expenditure from earlier work using assumptions are likely to have been underestimated and the “central” estimate of health opportunity cost from that work (£12,936 per QALY) to have been overestimated.

A number of countries use well-established methods of economic evaluation^1^ to inform decisions about which new medical interventions warrant funding; examples include the National Institute for Health and Care Excellence (NICE) in the United Kingdom, Canadian Agency for Drugs and Technologies in Health (CADTH) in Canada, Autoridade Nacional do Medicamento e Produtos de Saúde, I. P. (INFARMED) in Portugal, and Health Intervention and Technology Assessment Program (HITAP) in Thailand.^2–4^ Economic evaluation identifies evidence on the expected health effects and costs of the intervention in relation to relevant alternatives. However, to fully inform a decision, there needs to be some consideration of how any health gains offered by the new intervention are to be assessed against any additional costs it imposes on health systems. A key piece of information to guide this assessment is an estimate of the health gains that could have been achieved elsewhere with the same levels of investment—the health opportunity costs—that is, to consider the health effects that could be generated by making the additional resources required for the new interventions available for other services and interventions that could be funded instead or the health effects of those activities that would need to be given up if these resources are committed to the new intervention.

A number of studies in different countries have based an assessment of opportunity costs on the empirical relationship between changes in health care expenditure and health outcome.^5–8^ Recent research in the United Kingdom used national data on expenditure and outcomes in different disease areas reported at a local level in the National Health Service (NHS).^9–11^ By exploiting the variation in expenditure and mortality outcomes, the relationship between changes in expenditure and mortality was estimated (while accounting for endogeneity). By using the effect of expenditure on the mortality and life-year burden of disease as a surrogate for the effects on a more complete measure of burden (one that also includes the quality-of-life burden of disease), a cost per quality-adjusted life-year (QALY) that reflects the likely impact of changes in expenditure on both mortality and morbidity was also reported.

These estimates of the marginal productivity of health care expenditure indicate the health that is expected to be forgone as a consequence of additional costs displacing other health care activities. They reflect what is likely to happen in the health care system, given current levels of information, local decision making, and the influence of other aspects of social value, which are not captured in measures of health such as QALYs. They represent the relevant expected health opportunity costs when the decision context is restricted to approving or rejecting a new intervention.^i^ In this context, it also indicates the maximum that the health care system can afford to pay for the additional benefits offered by a new intervention (e.g., the temporary monopoly price for pharmaceuticals protected by patent) without reducing the total number of QALYs generated.

The assumptions that were required to link the estimates of effects of changes in expenditure on the mortality burden of disease to the likely effect on QALYs constitute important sources of uncertainty. To inform these assumptions appropriately, the judgments of key individuals, such as those with substantive clinical or policy expertise, are important. Elicitation methods offer a systematic process for formalizing and quantifying, typically in probabilistic terms, individuals’ judgments about uncertain quantities.^12,13^

Elicitation is an important activity in many fields, including in support of decision making, where there may be significant uncertainties and their quantification can feed directly onto decisions. Furthermore, elicitation is a vital element of a Bayesian approach to statistics, the principles of which are core to decision analyses. Here, the use of prior information to augment existing data has an established theoretically basis, particularly where the empirical evidence is limited.^12^

This research presents an application of structured elicitation to inform estimates of expected health opportunity costs in the UK NHS, a key quantity to inform policy decisions. This constitutes a novel and important context for the use of structured elicitation, aiming to reflect uncertainty in the judgments required for policy appropriately and explicitly. We demonstrate the applicability of the elicitation exercise in practice. Its design draws from wider experience of elicitation in health technology assessment^14^ and literature from other areas of science (for example, refs. 15 and 16).

This article is structured as follows. The next section summarizes earlier work by Claxton et al.^9^ to estimate NHS marginal productivity and is the motivation for the current work. The following sections focus on the elicitation exercise, presenting its methods (design, conduct, and analyses) and the results of its application. The article finishes with a discussion including key policy implications.

## Summary of the Work by Claxton et al. and Overview of the Key Uncertainties Identified

Claxton et al.^9^ evaluated the relationship between expenditure and mortality using a cross-sectional design, seeking to identify differences in mortality across health care commissioning units (at the time of this research, there were 152 primary care trusts) that could be attributed to differences in NHS spend. Empirically, the research first quantified expenditure elasticities, that is, how changes in NHS expenditure in a given year were allocated between Programme Budgeting Categories (PBCs), which reflect broad disease areas characterized by International Classification of Disease (ICD) codes.

Second, the research estimated outcome elasticities, that is, how changes in expenditure by a PBC (in a particular year) altered PBC-specific mortality rates (using national data on mortality reported for ICDs or groups of ICDs, mapped onto PBCs). Analyses adjusted for important covariates (including need) and used instrumental variables to estimate causal effects overcoming the problem of endogeneity.

Results showed that the mortality effects of changes in spend could be identified for only 11 of the 23 PBCs (such as cancer and gastrointestinal disorders). For the remaining disease areas (such as mental health disorders), health care focuses primarily on improving health-related quality of life (HRQoL). Across the 11 PBCs for which mortality effects were detected, empirically based estimates of how changes in total NHS expenditure affect mortality were generated, returning the following point estimates (using 2008 expenditure and 2008–2010 mortality): £105,872 for the cost per death averted, £23,360 for the cost per life-year, and £28,045 for the cost per life-year where life-years were adjusted for HRQoL.

However, an estimate of health opportunity costs relevant for policy needs also to consider the following (Table 1):

A. whether changes in expenditure have effects beyond the year of expenditure (this can be termed *duration of effects*),
B. how the effects of changes in expenditure on mortality relate to effects on a broader measure of health that incorporates both duration and HRQoL impacts (QALYs; this can be termed *surrogacy*), and
C. how changes in expenditure affect health in disease areas for which the previous work could not measure a mortality effect (this can be termed *extrapolation*).

In the original research,^9^ very limited data were available with which to assess each of these questions, and hence assumptions were made (listed in Table 1). These were used to obtain a central estimate of health opportunity costs (expressed as a cost per QALY) across all disease areas of £12,936 per QALY. An analysis of the uncertainty imposed by the empirical estimates (the expenditure elasticities estimated for each of the 23 PBCs and the outcome elasticities estimated for 11 of these) indicated that the probability of this central estimate being less than £20,000 per QALY was 0.89.^9^

**Table 1:** Key Uncertainties and Assumptions Made in the Original Work by Claxton et. al.^9^

Key Uncertainty		Description	Assumptions on Key Uncertainties in Claxton et al.^9^
A	Duration of effects	Changes in expenditure may have an effect on mortality beyond the year of expenditure	Effects restricted to the year of expenditure change
B	Surrogacy	How the effects of changes in expenditure on mortality relate to effects on a broader measure of health that incorporates both duration and health-related quality-of-life effects (quality-adjusted life-years)	Assumed to be proportionate; that is, the effects of changes in expenditure on mortality that were empirically estimated were used as the best estimate of the effects of expenditure on quality-adjusted life-years
C	Extrapolation	How changes in expenditure affect health in disease areas for which previous work could not measure a mortality effect	Assumed to be proportionate; that is, the effects of changes in expenditure on health (quality-adjusted life-years) for disease areas for which previous work could measure a mortality effect were used as the best estimate of the effect of expenditure on health for disease areas for which previous work could not measure a mortality effect

## Methods

This research aimed at formally eliciting the beliefs of key individuals on the 3 judgments outlined above (and in Table 1), which are required for a policy-relevant estimate of health opportunity costs. Another uncertain quantity that was elicited concerned the expected life-years gained from averting a death. This is not required to evaluate health opportunity costs in terms of QALYs (although it is important to distinguish morbidity from mortality impacts on the QALY estimate), and hence, for conciseness, methods and results of the elicitation for this quantity are not described in this article but are available elsewhere.^16^ Uncertainty in knowledge was explicitly elicited throughout.^12,17,18^ The design of the exercise sought to minimize the use of cognitive heuristics that may lead to bias.^19–21^

Two groups of individuals were considered: the first comprised clinical experts, acting as substantive experts in key disease areas, and the second included policy experts, defined as individuals drawn from organizations that develop or implement policy or that have a major interest in policy in this area. These individuals are not expected to have specific substantive expertise in key clinical areas. Policy experts were asked for their judgments on the quantities of interest once they had considered the information that had been elicited from clinical experts. As such, the elicited judgments from policy experts reconcile their own judgments together with the views of the substantive (clinical) experts.

This exercise did not seek to establish consensus, as such methods are known to have a number of limitations (e.g., because of the fact that aggregation is done implicitly, dominant individuals may imbalance group dynamics, and consensus methods are known to return overly precise judgments).^22^ Hence, experts were asked to give their opinions individually (and discouraged from interacting), and a group estimate was generated analytically (detailed below).^12^

All aspects of the exercise (design, conduct, and analyses) were protocolled in advance.^23^

### What Quantities Were Elicited?

The elicitation questionnaire focused on the effects on the population health of changes in NHS expenditure in a particular year (all else unchanged). Experts were prompted to think of changes in expenditure that were significant but still represented a small proportion of NHS expenditure.

The first uncertain quantity concerned the duration of effects. A 2-part question was used (section A, Table 2) that first asked about the duration of mortality effects beyond the first year. Second, it asked about the magnitude of mortality effects in the second, third, and fourth years after the change in expenditure. Participants were asked to express the latter as a proportion of the effect in the first year, because the effect on the first year is an estimable quantity (and was the focus of the empirical work in Claxton et al.^9^). Using a relative quantity allows for conditional independence to be reasonably assumed and avoids the burdensome task of eliciting dependency. Conditional independence was also assumed in the elicitation of other uncertain quantities, and the accompanying diagram in Table 2 illustrates the conditional relationships specified. Note that the wording intentionally asked for the effects that can be attributed to changes in expenditure in a particular year and hence was able to identify future (lagged) effects causal to that year’s change in spend.

**Table 2:** Summary of the Wuantities Elicited with Diagrammatic Representation

Question	Diagramatic Representation
Section A *Question.* For how many more years (beyond the year of increased expenditure) would you expect disease-specific mortality rates to be reduced? *Question.* From an increase in expenditure in a particular year, how do reductions in mortality rates in subsequent years compare (in proportionate terms) to the reduction observed in the first year? This was elicited separately for the second, third, and fourth years. Refers to quantities A2 yr, A3 yr, and A4 yr, respectively, in the diagram.	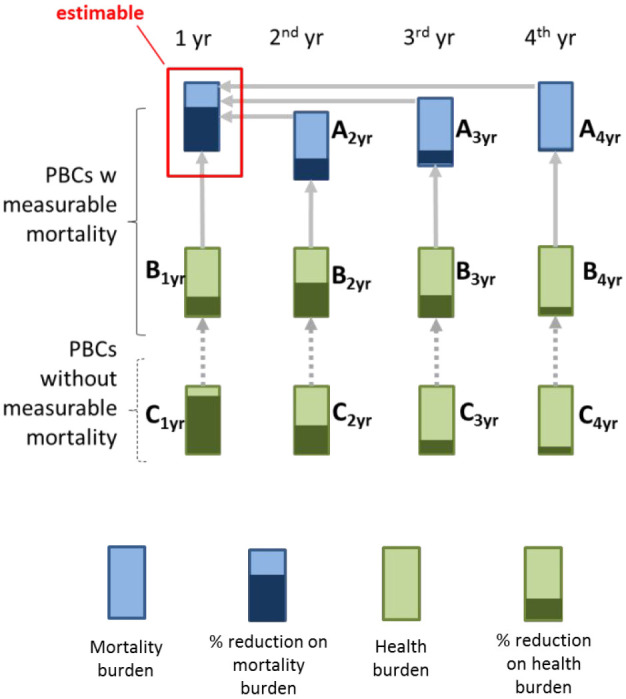
Section B *Question*. If expenditure is increased in a particular year, how many times bigger (or smaller) are proportionate reductions on quality-adjusted life-year burden when compared with proportionate reductions on mortality burden? We elicited for the year of increased expenditure (first year) and also for any later effects of expenditure on the second, third, and fourth years subsequent to increased expenditure. Refers to quantities B1 yr, B2 yr, B3 yr, and B4 yr, respectively, in the diagram.	
Section C *Question*. How much bigger (or smaller) are reductions in health burden (quality-adjusted life-years) when expenditure is increased, for example, in “mental health disorders” instead of disease areas with a measured effect of increased expenditure on mortality (average effect across all disease areas in this group). This was elicited for the year of expenditure (first year) and also for any later effects of expenditure on subsequent years (second, third, and fourth years). Refers to quantities C1 yr, C2 yr, C3 yr, and C4 yr, respectively, in the diagram.	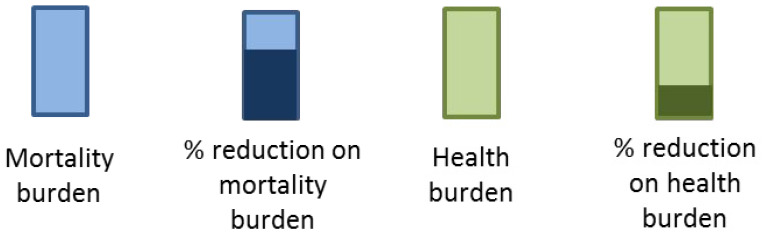

The second uncertain quantity subject to elicitation related to the surrogacy relationship and aimed to establish the effects of increased expenditure on a year’s QALY burden (section B, Table 2). QALY burden was defined as comprising of the life-years lost due to premature mortality (due to disease) in the year of interest, adjusted for quality, plus any impacts on the level of HRQoL from disease in individuals alive in that year. This was elicited separately for the year of expenditure (first year) and subsequent years (second, third, and fourth years). To allow for conditional independence, it was formulated as relative to effects on mortality burden in the same year.

The third uncertain quantity related to extrapolation (section C, Table 2). Experts were asked about reductions in QALY burden in disease areas that did not have measurable mortality effects (e.g., mental health). They were asked to express these reductions proportionally in relation to the average QALY burden reduction from an increase in NHS expenditure across all disease areas with measurable mortality effects. Again, this was elicited separately for the year of expenditure (first year) and subsequent years (second, third, and fourth years).

Although elicited judgments are likely to differ between disease areas, it was considered too burdensome for the experts to present their judgments for each of the 23 PBCs. Hence, 7 disease areas (circulatory, respiratory, gastrointestinal, neurological, mental health, endocrinology, musculoskeletal) were selected. These were chosen because changes in expenditure and changes in mortality in those areas are the most important drivers of the central estimate of health opportunity cost and most sensitive to the surrogacy and extrapolation assumptions. Estimates were elicited from experts separately for each of these 7 main PBCs and a single estimate for the remaining PBCs combined. These are heterogeneous and broad disease areas, so in responding to questions, experts were asked to consider the ICDs within each PBC for which an increase in expenditure is more likely to fall.

### Which Experts?

We aimed to recruit purposively 20 clinicians (at least 2 from each clinical area^ii^) and 20 individuals affiliated with selected policy-relevant organisations.^iii,24^ Responses from experts were anonymous, but the organizations they belong to were recorded (policy experts), as were the clinical areas of expertise (clinical and relevant policy experts), to facilitate analysis of between-expert heterogeneity.^14^

### How Were the Different Quantities Elicited?

It was important for elicitation to reflect experts’ uncertainty, so experts were asked for multiple summaries on each quantity.^12^ One was the mode (the value the expert believes to be most likely, their best guess) as it is generally thought that experts can more easily report this than the mean or median.^12,25^ The other summary estimates were the bounds of a credible interval (Crl; the Bayesian equivalent to confidence intervals).^iv^ Evidence shows that while eliciting CrI is intuitive, there is a clear tendency for these to be too narrow (a bias called “overconfidence”); that is, people believe their estimates are more accurate than is justified.^26^ This limitation is acknowledged, but experts’ time constraints were a major consideration.^27^ Hence, strategies were adopted to minimize the potential for bias: 80% CrI were elicited as these typically show less overconfidence than 95% CrIs,^12^ and single limit estimates were also elicited—in which the lower bound is elicited first and then the upper bound separately—as these are also thought to produce wider estimates than asking directly for the range.^28,29^ Hence, the wording used in this work was as follows:

(Mode) My best guess for the value of this quantity is . . . .

(Lower bound of 80% CrI): I am very certain (90% certain) that the true value for this quantity is *higher* than . . . .

(Upper bound of 80% CrI): I am very certain (90% certain) that the true value for this quantity is *lower* than . . . .

### Conduct of the Exercise

A paper questionnaire was developed (Supplementary Appendix 1) and extensively piloted. To facilitate appropriate training, the exercise was, where possible, conducted in groups (workshops). A training session for experts was developed that described the objectives of the elicitation exercise; clarified concepts such as those of uncertainty, variability, and heterogeneity; familiarized experts with the quantities the research sought to elicit; described and explained the impact of bias and heuristics; and trained experts on the methods of elicitation used (Supplementary Appendix 2).^30–32^ This was delivered by 2 of the authors (K.C. and M.O.S.).

Throughout the exercise, individuals were encouraged to revisit and revise their answers to previous questions,^33^ but we did not record when this occurred. At the end of each section of the exercise, participants were asked whether they were confident the answers they had given reflected their views and uncertainties. Response options were “yes,”“not sure,” and “no.” Individuals were also provided with opportunities for free-text feedback.

The judgments from clinical experts were elicited prior to those of policy experts. The judgments from clinical experts were summarized (histograms of the modes and upper and lower CrI bounds) and presented to policy experts to help them formulate their judgments using the same elicitation tool (Supplementary Appendix 3).

### Analyses and Pooling across Experts

Analyses were conducted in Excel 2010.^34^ In describing the elicited beliefs, the first step was to fit a distribution to each quantity elicited from each individual expert.^30,35^ The quantities of interest here ranged between 0 and +infinity and were fitted with the log-normal distribution as prespecified.^23^ Given that 3 summaries were elicited from each expert, more than 1 type of 2-parameter distribution can reasonably reflect their judgments. It was protocolled^23^ that, to reflect this additional uncertainty, 2 alternative (2-parameter) distributions would be fitted: one using the lower bound of the CrI and the mode and another using the upper bound and the mode.^v^ A unique distribution for each quantity elicited by each expert was then derived by linear pooling of the 2 distributions (i.e., pooling means and variances).^vi^ Further details on this stage of analysis is presented in Supplementary Appendix 4.

After describing each expert’s judgment for each quantity using distributions, these were pooled together to derive a single distribution for the group. Linear pooling was used^12^ with equal weights across experts^4^ to preserve the individual judgments in the collective (pooled) judgment.^14,26^ Linear pooling means that, if the experts’ distributions for a single quantity are identical, the pooled distribution is also identical to the individuals’ distributions. Also, if there is the support from at least 1 expert that the quantity of interest takes a particular value, the pooled distribution will also show some support for that value.^12,36^

The primary analysis reflects the pooled results from clinical experts, and the secondary analysis reflects the pooled results from policy experts.

### Sensitivity Analyses

Two sensitivity analyses were protocolled.^23^ One explored heterogeneity (i.e., between-expert uncertainty) by 1) considering only responses of clinical experts in the clinical specialty relating to the disease area in the question and 2) by grouping policy experts based on the type of organization to which they belonged (see footnote iii). The second protocolled sensitivity analysis disregarded those responses when individuals indicated they were not confident that the response reflected their views and uncertainties. A third and final sensitivity analysis was not protocolled and provided a qualitative assessment of the implications of using a Gamma distribution, instead of the log-normal, in the fitting.

## Results

### Primary Analyses Using Substantive (Clinical) Experts’ Responses

Twenty-eight clinical experts participated in 3 (group) workshops and 4 individual interviews.^vii^ A summary of the pooled distributions across all clinical experts is presented in Table 3.

**Table 3:** Duration of Effects, Surrogacy and Extrapolation – All Clinical Experts Pooled

		Year 1	Year 2	Year 3	Year 4	Total Additional Duration (y)^a^
		Mode [Mean] (Lower, Upper Bounds of the 80% Credible Interval)				
Circulatory	Mortality effects (v. year 1)	*Estimable from data*	0.2 [1.5] (0.2, 3.4)	0.1 [1.2] (0.1, 2.6)	0 [0.9] (0.1, 2.1)	3.4 [11.2] (2.4, 23.7)
	Surrogacy (v. same year)	0.3 [2.9] (0.3, 6.6)	0.4 [2.9] (0.4, 6.6)	0.3 [2.9] (0.3, 6.5)	0.2 [2.9] (0.2, 6.6)	—
Respiratory	Mortality effects (v. year 1)	*Estimable from data*	0.1 [1.5] (0.1, 3.4)	0.1 [0.7] (0.1, 1.6)	0 [0.6] (0, 1.5)	1.3 [8.9] (1.1, 20.1)
	Surrogacy (v. same year)	0.3 [3.8] (0.3, 8.7)	0.2 [3.9] (0.3, 8.8)	0.1 [3.4] (0.2, 7.5)	0.1 [3.4] (0.1, 7.4)	—
Gastrointestinal	Mortality effects (v. year 1)	*Estimable from data*	0.1 [1.7] (0.1, 3.8)	0 [1.1] (0, 2.4)	0 [0.9] (0, 1.9)	0.7 [11.4] (0.8, 26)
	Surrogacy (v. same year)	0.4 [3.6] (0.4, 8.2)	0.1 [4.5] (0.2, 9.9)	0.2 [4.3] (0.2, 9.6)	0.2 [4.2] (0.2, 9.4)	—
Neurological	Mortality effects (v. year 1)	*Estimable from data*	0 [1.3] (0, 2.8)	0 [0.9] (0, 1.9)	0 [1] (0, 1.9)	0.7 [5.9] (0.7, 13.2)
	Surrogacy (v. same year)	0.4 [4.3] (0.4, 9.7)	0.7 [3.3] (0.5, 7.3)	0.7 [2.9] (0.5, 6.4)	0.3 [3.2] (0.3, 7.4)	—
Endocrinology	Mortality effects (v. year 1)	*Estimable from data*	0.1 [1.4] (0.1, 3.1)	0 [1] (0.1, 2.3)	0 [0.6] (0, 1.4)	2 [9] (1.5, 19.7)
	Surrogacy (v. same year)	0.1 [4.7] (0.1,10)	0.1 [6.2] (0.2, 13.2)	0.1 [5.2] (0.1, 10.6)	0 [5.5] (0.1, 11.1)	—
Others with mortality	Mortality effects (v. year 1)	*Estimable from data*	0.1 [1.8] (0.1, 4)	0 [1.1] (0, 2.4)	0 [0.9] (0, 1.8)	2.1 [9.5] (1.6, 20.8)
	Surrogacy (v. same year)	0.1 [4.7] (0.2, 10.3)	0.3 [5.4] (0.3, 12.2)	0.3 [6.4] (0.3, 14.3)	0.2 [8.8] (0.3, 19.2)	—
Mental health	Extrapolation (v. same year)	0.7 [4] (0.6, 8.8)	0.6 [3.8] (0.5, 8.5)	0.6 [3.6] (0.5, 7.9)	0.6 [3.3] (0.5, 7.3)	—
Musculoskeletal		0.5 [4.7] (0.5, 10.7)	0.5 [4.1] (0.4, 9.3)	0.6 [3.4] (0.5, 7.5)	0.6 [3.1] (0.5, 7)	—
Others without mortality		0.6 [3.4] (0.5, 7.5)	0.4 [3.2] (0.4, 7.1)	0.7 [2.5] (0.5, 5.3)	0.6 [2.6] (0.4, 5.8)	—

a Beyond the year of increased expenditure.

**Figure 1:**
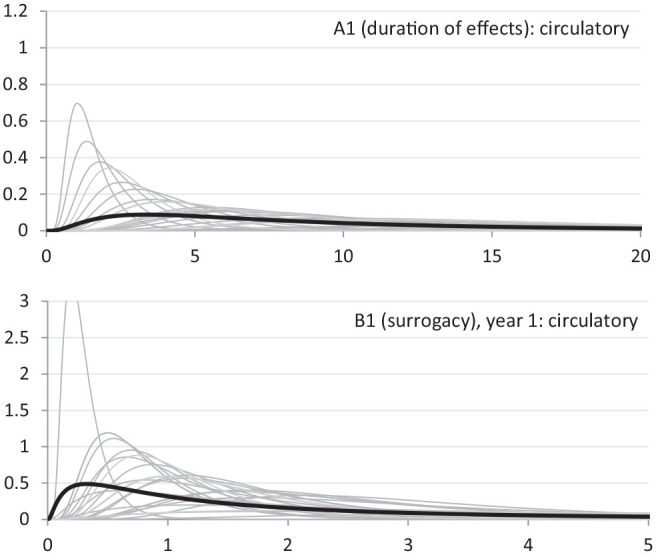
Illustration of individual experts’ fitted distributions (gray) and the pooled distribution (black) of clinical experts.

Results of question A1 (duration of effects) indicate that changes in NHS expenditure in a particular year are expected to affect mortality in subsequent years. The mean duration of effects is highest for circulatory and gastrointestinal (approximately 11 additional years) and lowest for neurological disease (approximately 6 additional years). The pooled distribution shows considerable uncertainty, as demonstrated by its wide 80% CrI. As an illustration, the top panel of Figure 1 shows the individual experts’ distributions for the duration of effects in circulatory disease (in gray), overlaid with the pooled distribution across all experts (in black). Note that the uncertainty in the pooled distribution reflects not just each individual’s uncertainty but also between-expert heterogeneity.

Experts’ judgments suggest that, across all disease areas, mortality effects beyond year 1 are expected to be higher than effects in the first year (section B). In circulatory disease, for example, it is expected that the effect in the second year is 1.5 times that in the first year. This can be interpreted to reflect the preventative nature of much of the expenditure in this disease area, in which health benefits of current expenditure are higher in the future. The magnitude of expected mortality effects decreases over time for all disease areas. For example, in circulatory disease, surrogacy on the third year is expected to be 1.2 and in the fourth year 0.9. The pooled distributions are wide, and the 80% CrI includes the value of 1.

Experts’ judgments indicate that surrogacy relationships are expected to be greater than 1 in the year of expenditure for all disease areas (between 2.9 and 3.7, see Table 3). This implies that changes in spend are expected to reduce QALY burden proportionately more than mortality burden, although this is associated with considerable uncertainty. The individual experts’ distributions on the surrogacy relationship in year 1 for circulatory disease have been graphically presented in the bottom panel of Figure 1. Only 5 of the 27 distributions (1 expert did not complete this question) have mean estimates below or equal to 1 (results not presented here). The pooled distribution across the 27 experts shows a mean of 2.9 and an 80% CrI suggesting the true value lies between 0.3 and 6.6 (Table 3). Over time, expected values for surrogacy do not fall below 1.

Extrapolation relationships follow the same pattern as surrogacy, with expected values consistently above 1 (between 2.6 and 4.7). The 80% CrI seem to reduce width over time.

### Secondary Analysis Using Policy Experts’ Responses

Twenty-five policy experts participated in 2 workshops (affiliations in endnote viii). Table 4 presents a summary of pooled distributions.

**Table 4:** Duration of Effects, Surrogacy and Extrapolation – All Policy Experts Pooled

		Year 1	Year 2	Year 3	Year 4	Total Additional Duration (y)^a^
		Mode [Mean] (Lower, Upper Bounds of the 80% Credible Interval)				
Circulatory	Mortality effects (v. year 1)	*Estimable from data*	0.2 [1.4] (0.2, 3.1)	0.3 [1] (0.2, 2)	0.2 [0.8] (0.1, 1.7)	1.2 [15.1] (1.2, 34.3)
	Surrogacy (v. same year)	0.2 [2.8] (0.2, 6.3)	0.3 [2.3] (0.2, 5.1)	0.3 [2.3] (0.3, 5.2)	0.3 [2.4] (0.3, 5.4)	—
Respiratory	Mortality effects (v. year 1)	*Estimable from data*	0.3 [1.1] (0.2, 2.3)	0.2 [0.6] (0.1, 1.4)	0 [0.6] (0, 1.4)	0.4 [11.3] (0.5, 25)
	Surrogacy (v. same year)	0.6 [2.4] (0.4, 5.2)	0.5 [2.7] (0.4, 6.1)	0.3 [2.6] (0.3, 5.9)	0.3 [2.9] (0.3, 6.5)	—
Gastrointestinal	Mortality effects (v. year 1)	*Estimable from data*	0.1 [2] (0.1, 4.6)	0.1 [1.6] (0.1, 3.6)	0 [1.9] (0.1, 4.1)	1.2 [16.9] (1.3, 38.4)
	Surrogacy (v. same year)	0.4 [2.8] (0.3, 6.3)	0.4 [2.9] (0.3, 6.5)	0.4 [3.1] (0.3, 6.9)	0.5 [2.7] (0.4, 5.9)	—
Neurological	Mortality effects (v. year 1)	*Estimable from data*	0.3 [1.3] (0.2, 2.9)	0.2 [1.2] (0.1, 2.6)	0.1 [1.2] (0.1, 2.8)	1 [17.8] (1.2, 40.2)
	Surrogacy (v. same year)	1.2 [2.9] (0.8, 5.8)	1 [2.7] (0.7, 5.6)	0.7 [2.5] (0.5, 5.3)	0.7 [2.3] (0.5, 4.9)	—
Endocrinology	Mortality effects (v. year 1)	*Estimable from data*	0.1 [1.9] (0.1, 4.3)	0.1 [1.3] (0.1, 2.8)	0.1 [1.1] (0.1, 2.4)	0.6 [12.6] (0.7, 28.3)
	Surrogacy (v. same year)	0.8 [2.4] (0.5, 5)	0.7 [2.6] (0.5, 5.5)	0.7 [2.6] (0.5, 5.6)	0.7 [2.8] (0.5, 6.2)	—
Others with mortality	Mortality effects (v. year 1)	*Estimable from data*	0.1 [1.3] (0.1, 3)	0.3 [0.9] (0.2, 1.8)	0.2 [0.9] (0.1, 1.9)	0.7 [13.5] (0.8, 30.4)
	Surrogacy (v. same year)	0.3 [2] (0.3, 4.5)	0.3 [2.4] (0.3, 5.3)	0.5 [2.1] (0.3, 4.6)	0.2 [2.2] (0.2, 5.1)	—
Mental health	Extrapolation (v. same year)	1 [3.9] (0.7, 8.4)	1 [3.4] (0.7, 7.3)	0.8 [3] (0.6, 6.4)	0.7 [2.9] (0.5, 6.2)	—
Musculoskeletal		0.6 [5] (0.5, 11.2)	1.2 [3.1] (0.8, 6.4)	0.9 [2.7] (0.7, 5.7)	0.7 [2.5] (0.5, 5.3)	—
Others without mortality		0.3 [4.2] (0.3, 9.6)	0.3 [3] (0.3, 6.9)	0.6 [2.5] (0.4, 5.4)	0.5 [2.3] (0.4, 4.9)	—

a Beyond the year of increased expenditure.

**Figure 2:**
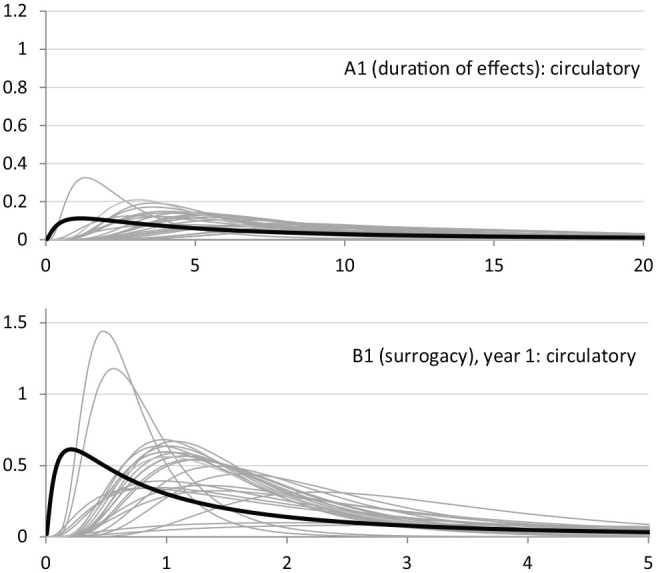
Illustration of individual experts’ fitted distributions (gray) and the pooled distribution (black) of policy experts.

Results were fairly similar to those obtained with the pool of clinical experts, but between-expert variation was lower for this group of experts (exemplified in Figure 2 for duration of effects, top panel, and surrogacy, bottom panel, in circulatory disease). With respect to mortality effects, policy experts generally indicated higher duration (in terms of expected values) than clinical experts and a similar magnitude over time.

In terms of surrogacy, expected values are also comparable with those of clinical experts. Expected values do not fall below 1 (although CrI include 1); for example, for respiratory, surrogacy had an expected value of 2.9. Expected extrapolation relationships also follow similar patterns to those of clinical experts but decrease slightly faster over time.

### Face Validity and Qualitative Feedback

The information provided by individual experts is reproduced in item 4 of the Supplemental Material. Only a very small proportion of clinical experts (1/28 in section A, 3/28 in section B, and 0/24 in section C) indicated their responses did not reflect their views and uncertainties, with the remaining answering “yes” or “unsure” (respectively, 16 and 11 out of 28 in section A, 7 and 19 out of 28 in section B, and 14 and 10 out of 24 in section C). This was qualitatively similar for policy experts. Qualitative feedback was insightful regarding the reasons for these responses. Participants, both clinical and policy, consistently mentioned that the heterogeneity across the ICDs that composed the different disease areas made responding to questions particularly challenging. Some clinical experts also found it difficult to answer questions on disease areas that did not relate to their specialism. Some policy experts also indicated that they relied heavily on the clinical experts’ answers. The qualitative feedback did not suggest that the answers lacked face validity but instead explains the wide distributions returned by participants.

### Sensitivity Analysis

Results of sensitivity analyses are shown in full in Supplementary Appendix 5. Here, we present only a qualitative summary of results.

Results did not change meaningfully when removing individuals who indicated their responses did not reflect their views and uncertainties (item 2.1A in Supplementary Appendix 5). When also removing individuals who responded “not sure” to this question (i.e., considering only those who responded “yes”), differences were again not meaningful, except for surrogacy, for which means were slightly higher across all disease areas (item 2.1B in Supplementary Appendix 5). In terms of heterogeneity in the primary analysis (item 2.2 in Supplementary Appendix 5), the pooled distribution of clinicians in their clinical area of expertise shows some differences in relation to the pooled results across all clinicians (see, for example, the mean duration of mortality effects for circulatory, gastrointestinal, and neurological diseases). The magnitude of such effects over time is (in general) higher for circulatory and neurological diseases. Expected surrogacy relationships are similar for the year of expenditure, except for neurological disease, for which experts indicate surrogacy to be higher. Expected extrapolation relationships are lower for mental health, in the first year and over subsequent years, but higher for the first year in musculoskeletal disease.

In terms of heterogeneity in secondary analyses (item 2.3 in Supplementary Appendix 5), of note is the pooled distribution for group G2 (the biggest group comprising of 15 of the total 25 experts, including “governmental bodies,” such as the Department of Health and Social Care or Public Health England), which presents generally lower expected values and more precise distributions than the overall group. This implies that the heterogeneity introduced by the remaining groups is contributing to a widening of the CrI.

The post hoc sensitivity analyses evaluating an alternative distribution to represent experts’ beliefs (item 2.4 in Supplementary Appendix 5) shows overall conclusions to be robust but that the magnitude of effects is sensitive to the choice: the log-normal distribution (prespecified in our analyses plan) has a heavier tail than the Gamma (implemented in sensitivity analyses) and hence generally returns higher expected values when fitted to the same mode and CrI bounds.

## Discussion

This research developed an exemplar elicitation exercise aimed at quantitatively gathering the (uncertain) beliefs of individuals on a set of quantities for which there is currently insufficient evidence but that are central to an estimate of health opportunity costs for the UK’s NHS. Resourcing decisions in the NHS require consideration of health opportunity costs, and hence this work has direct relevance for current policy in the United Kingdom. Despite being motivated by earlier research,^9^ this work will also have longer-term relevance as the judgments elicited can be used to support other empirical studies for the United Kingdom, including those using different econometric methodologies, as these can be expected to suffer from the same evidence gaps.

Elicited judgments should not replace high-quality evidence, and it is paramount that primary evidence is collected on each of the uncertain quantities covered here. Our work, however, was designed in such a way that, as new evidence reports on individual quantities, the judgments elicited on the other quantities can be retained for use in policy. This was achieved by defining quantities as conditionally independent. The work presented here is also important internationally, as it can be adapted for evaluations pertaining to other countries or settings, beyond the UK’s NHS.

The group estimates obtained provide a summary of the beliefs of multiple experts on quantities for which there currently is no evidence. There are, therefore, important implications for a meaningful estimate of health opportunity costs for use in policy. First, regarding the duration of mortality effects, the original analyses^9^ assumed impacts only in the year of expenditure. The results from the current work, however, indicate that mortality effects are expected also to occur in subsequent years. This suggests that the original work underestimated the QALY impacts of changes in expenditure. Second, the original work assumed perfect surrogacy in the effects of changes expenditure between mortality burden and total QALY burden. The results from this research indicate, however, that surrogacy is expected to be greater than 1 (this holds across disease areas for the first, second, and third years), indicating that the effects of changes in expenditure on total QALY burden are, in proportionate terms, expected to be higher than (rather than equal to) those on mortality burden. Again, this suggests that the original work underestimated the QALY impacts of changes in expenditure. Third, in terms of extrapolation, the original work assumed changes in spend to have equal effects on diseases with, and without, measured mortality effects. This work demonstrates that the extrapolation relationship is generally expected to be greater than 1. That is, the health effects in disease areas without measured mortality effects are expected to be higher than what was assumed in the original work. Consistently across the 3 uncertainties, experts’ judgments suggest the QALY impact of changes in expenditure are likely to be underestimated when using the assumptions that underpin the “central” estimate of £12,936 per QALY reported in Claxton et al.^9^

The exercise was carefully developed to align with the scope of the policy question, was piloted extensively, and was accompanied by an extensive training package to support experts and guide them through the tasks. As a consequence, it ran successfully. Experts were able to express their beliefs quantitatively, with only a few indicating their answers did not reflect their views (i.e., were not face valid). However, in approximately half of the answers, individuals indicated they were unsure that their answers reflected their views or uncertainties. Feedback left in open text did not, however, indicate these answers were not face valid but instead suggested that the breadth of the questions meant that the distributions retrieved were wide. Convening individuals in groups aided the delivery of the standardized training package and maximized expert engagement. However, it also made recruitment difficult: 132 clinical and 84 policy experts were contacted to recruit effective samples of 28 and 25, respectively. Issues with recruitment in elicitation have been recognized elsewhere.^27^

As expected, the level of uncertainty in knowledge expressed by the individual experts was large, and group estimates were highly uncertain (as evident by the wide CrIs). In their feedback (Supplementary Appendix 6), experts consistently indicated that heterogeneity in the broad disease areas contributed to the uncertainty expressed in their responses. However, eliciting for “finer” definitions of disease, for example, 3-digit ICD codes of which there are more than 1500, would have been unfeasibly burdensome. Therefore, future research could instead provide further information to experts to help them make judgments about which ICDs may matter the most within each disease area.

The design of an elicitation exercise requires a number of methodological choices to be made, many of which are example specific. This exercise used methods established in the literature and justifies the choices made. However, it is important to acknowledge that methods research in this area is limited and that little is known about how different choices affect results. For example, although there is some evidence that consensus methods present a number of challenges inherent to group interaction (see the Methods section), its accuracy in relation to individual elicitation is largely unknown.

This article demonstrates that structured elicitation can feasibly be used to explicitly quantify the judgments required to delimit important policy problems, judgments that otherwise would still need to be made implicitly and without the support of relevant experts. In this work, we focused on achieving a relevant estimate of health opportunity costs, a central quantity for policy on health care resource allocation decisions. We have learned that the methods used here (i.e., the elicitation protocol) are applicable in this novel context. For example, the elicitation of the mode and bounds of an 80% CrI was widely understood by the experts, and experts working close to policy valued the summaries of the judgments of clinical experts provided. We also learned that there are challenges in eliciting policy-relevant, but broad-ranging, quantities. Such broad-ranging quantities are by definition uncertain, and structured expert elicitation makes this explicit.

## Supplemental Material

Manuscript_expert_elicitation_HOC_MDM_4_Appendix1_online_supp – Supplemental material for Health Opportunity Costs: Assessing the Implications of Uncertainty Using Elicitation Methods with Experts Click here for additional data file. Supplemental material, Manuscript_expert_elicitation_HOC_MDM_4_Appendix1_online_supp for Health Opportunity Costs: Assessing the Implications of Uncertainty Using Elicitation Methods with Experts by Marta O. Soares, Mark J. Sculpher and Karl Claxton in Medical Decision Making

Manuscript_expert_elicitation_HOC_MDM_4_Appendix2_online_supp – Supplemental material for Health Opportunity Costs: Assessing the Implications of Uncertainty Using Elicitation Methods with Experts Click here for additional data file. Supplemental material, Manuscript_expert_elicitation_HOC_MDM_4_Appendix2_online_supp for Health Opportunity Costs: Assessing the Implications of Uncertainty Using Elicitation Methods with Experts by Marta O. Soares, Mark J. Sculpher and Karl Claxton in Medical Decision Making

Manuscript_expert_elicitation_HOC_MDM_4_Appendix3_online_supp – Supplemental material for Health Opportunity Costs: Assessing the Implications of Uncertainty Using Elicitation Methods with Experts Click here for additional data file. Supplemental material, Manuscript_expert_elicitation_HOC_MDM_4_Appendix3_online_supp for Health Opportunity Costs: Assessing the Implications of Uncertainty Using Elicitation Methods with Experts by Marta O. Soares, Mark J. Sculpher and Karl Claxton in Medical Decision Making

Manuscript_expert_elicitation_HOC_MDM_4_Appendix4_online_supp – Supplemental material for Health Opportunity Costs: Assessing the Implications of Uncertainty Using Elicitation Methods with Experts Click here for additional data file. Supplemental material, Manuscript_expert_elicitation_HOC_MDM_4_Appendix4_online_supp for Health Opportunity Costs: Assessing the Implications of Uncertainty Using Elicitation Methods with Experts by Marta O. Soares, Mark J. Sculpher and Karl Claxton in Medical Decision Making

Manuscript_expert_elicitation_HOC_MDM_4_Appendix5_online_supp – Supplemental material for Health Opportunity Costs: Assessing the Implications of Uncertainty Using Elicitation Methods with Experts Click here for additional data file. Supplemental material, Manuscript_expert_elicitation_HOC_MDM_4_Appendix5_online_supp for Health Opportunity Costs: Assessing the Implications of Uncertainty Using Elicitation Methods with Experts by Marta O. Soares, Mark J. Sculpher and Karl Claxton in Medical Decision Making

Manuscript_expert_elicitation_HOC_MDM_4_Appendix6_online_supp – Supplemental material for Health Opportunity Costs: Assessing the Implications of Uncertainty Using Elicitation Methods with Experts Click here for additional data file. Supplemental material, Manuscript_expert_elicitation_HOC_MDM_4_Appendix6_online_supp for Health Opportunity Costs: Assessing the Implications of Uncertainty Using Elicitation Methods with Experts by Marta O. Soares, Mark J. Sculpher and Karl Claxton in Medical Decision Making

## Footnotes

i Decision makers may also compare the proposed investment to other specific disinvestments or alternative investments. However, they still need to consider how these compare with what the health care system would be expected to deliver (i.e., an estimate of marginal productivity is still relevant). If the decision maker had full information about all interventions that are or could be provided for all indications and subgroups of the population and was also tasked with the wholesale redesign of the health care system, well-established mathematical programming solutions would be possible and appropriate. The marginal productivity would be the outcome of this optimization (i.e., the shadow price of the expenditure constraint from solving the dual problem).

ii Primary care was added to the list for the broad expertise these individuals have of all clinical areas (and for this reason, these would be overrepresented in relation to others). Clinical experts were identified through involvement with decision-making bodies (e.g., NICE guidelines and appraisal committees), involvement with research, or by recommendation from colleagues.

iii Department of Health, NHS England (NHSE), Public Health England (PHE), National Institute for Health and Care Excellence (NICE), Joint Committee on Vaccination and Immunisation (JCVI), NHS Clinical Commissioners (NHSCC), Association of the British Pharmaceutical Industry (ABPI), patients’ organizations.

iv This is a variable interval method (in which the expert identifies values for the quantity that correspond to specified percentiles of his or her subjective distribution).

v The log-normal distribution was chosen for positive quantities because a closed-form solution to the percentiles and the mode is generally available. When using 2 summaries from experts, the parameters of the log-normal distribution can be directly evaluated, in closed form, without requiring simulation. However, in some cases in which a log-normal was fitted to a lower bound and mode from experts, there was no solution. In this case, minimum square errors were used to find the distribution parameters that provided the closest solution. This was implemented using the Solver Add-in of Excel, a simulation-based procedure.

vi Linear pooling takes the 2 (or more) distributions as 2 groups of the same population. Hence, the means and variances can be pooled directly using the following relations:$\begin{array}{c} \mu_{\mathrm{pooled}}=(\mu_{1}+\mu_{2})/2\\ {\sigma^{2}}_{\mathrm{pooled}}=\left(\sigma_{1}^{2}+\mu_{1}^{2}+\sigma_{2}^{2}+\mu_{2}^{2}\right)/2-\mu_{\mathrm{pooled}}^{2} \end{array}$where $\mu_{1}$ and $\sigma_{1}^{2}$ are the mean and variance, respectively, of the first distribution, and $\mu_{2}$ and $\sigma_{2}^{2}$ the mean and variance of the second distribution. Note that these 2 groups are equally weighted, which, in this case, means the sample size of each is assumed equal and thus does not appear in the equations.

vii Of the 28 experts recruited, 5 indicated circulatory expertise, 2 respiratory, 3 gastrointestinal, 2 neurological, 3 endocrinology, 3 mental health, 1 musculoskeletal, 3 primary care, 5 indicated other, and 3 indicated no clinical expertise. All except 2 individuals indicated only 1 area of expertise. Of these 2 experts, 1 indicated circulatory, neurological, musculoskeletal, and other (rehabilitation) and another indicated respiratory and primary care.

## References

[1] Drummond M Sculpher MJ Claxton K Stoddart GL Torrance GW, eds. Methods for the Economic Evaluation of Health Care Programmes. 4th ed. Oxford, UK: Oxford University Press; 2015.

[2] HITAP. 2017 Available from: http://www.hitap.net/en

[3] Infarmed. 2017 Available from: http://www.infarmed.pt/

[4] National Institute for Health and Care Excellence. 2017 Available from: https://www.nice.org.uk

[5] Vallejo-Torres L Garcia-Lorenzo B Serrano-Aguilar P. Estimating a cost-effectiveness threshold for the Spanish NHS. Health Econ. 2018;27(4):746–61. 10.1002/hec.3633 29282798

[6] Karnon J, ed. Estimating a reference ICER for Australia. Presented at: 12th International Health Economics Association (iHEA) World Congress; 2017; Boston, MA.

[7] Stadhouders N, ed. Estimating marginal benefits of healthcare spending in the Netherlands. Presented at: 12th International Health Economics Association (iHEA) World Congress; 2017; Boston, MA.

[8] Edoka I, ed. Estimating marginal returns to healthcare spending in South Africa: an instrumental variable approach. Presented at: 12th International Health Economics Association (iHEA) World Congress; 2017; Boston, MA.

[9] Claxton K Martin S Soares M, et al Methods for the estimation of the National Institute for Health and Care Excellence cost-effectiveness threshold. Health Technol Assess. 2015;19(14):1–503, v–vi. 10.3310/hta19140 PMC4781395 25692211

[10] Martin S Rice N Smith PC. Does health care spending improve health outcomes? Evidence from English programme budgeting data. J Health Econ. 2008;27(4):826–42. 10.1016/j.jhealeco.2007.12.002 18261812

[11] Martin S Rice N Smith PC. Comparing costs and outcomes across programmes of health care. Health Econ. 2012;21(3):316–37. 10.1002/hec.1716 21322086

[12] O’Hagan A. Uncertain Judgements: Eliciting Experts’ Probabilities. Chichester, UK: Wiley; 2006.

[13] Cooke R. Experts in Uncertainty: Opinion and Subjective Probability in Science. Oxford, UK: Oxford University Press; 1991.

[14] Soares MO Sharples L Morton A Claxton K Bojke L. Experiences of structured elicitation for model-based cost-effectiveness analyses. Value Health. 2018;21(6):715–23. 10.1016/j.jval.2018.01.019 PMC6021555 29909877

[15] European Food Safety Authority. Guidance on Expert Knowledge Elicitation in Food and Feed Safety Risk Assessment. EFSA J. 2014;12(6):3734.

[16] Dias LC Morton A Quigley JL, eds. Elicitation: The Science and Art of Structuring Judgement. New York: Springer; 2018.

[17] Hora SC. Aleatory and epistemic uncertainty in probability elicitation with an example from hazardous waste management. Reliab Eng Syst Safe. 1996;54(2–3):217–23.

[18] Griffin SC Claxton KP Palmer SJ Sculpher MJ. Dangerous omissions: the consequences of ignoring decision uncertainty. Health Econ. 2011;20(2):212–24. 10.1002/hec.1586 20091763

[19] Montibeller G von Winterfeldt D. Cognitive and motivational biases in decision and risk analysis. Risk Anal. 2015;35(7):1230–51. 10.1111/risa.12360 25873355

[20] Kynn M. The ‘heuristics and biases’ bias in expert elicitation. J Roy Stat Soc A Stat Soc. 2008;171:239–64.

[21] Tversky A Kahneman D. Judgment under uncertainty: heuristics and biases. Science. 1974;185(4157):1124–31. 10.1126/science.185.4157.1124 17835457

[22] Clemen RT Winkler RL. Combining probability distributions from experts in risk analysis. Risk Anal. 1999;19(2):187–203. 10.1111/0272-4332.202015 10859775

[23] (EEPRU) PRUiEEoHaHCI. Allocative efficiency 2017 Available from: http://scharr.dept.shef.ac.uk/eepru_wordpress/allocative-efficiency/

[24] Bolger F, ed. The selection of experts for (probabilistic) expert knowledge elicitation. In: Elicitation. New York: Springer; 2018 p. 393–443.

[25] Peterson C Miller A. Mode median + mean as optimal strategies. J Exp Psychol. 1964;68(4):363–7. 10.1037/h0040387 14217915

[26] Garthwaite PH Kadane JB O’Hagan A. Statistical methods for eliciting probability distributions. J Am Stat Assoc. 2005;100(470):680–700.

[27] Kadane JB Wolfson LJ. Experiences in elicitation. J Roy Stat Soc D Stat. 1998;47(1):3–19.

[28] Teigen KH Jorgensen M. When 90% confidence intervals are 50% certain: on the credibility of credible intervals. Appl Cogn Psych. 2005;19(4):455–75.

[29] Speirs-Bridge A Fidler F McBride M Flander L Cumming G Burgman M. Reducing overconfidence in the interval judgments of experts. Risk Anal. 2010;30(3):512–23. 10.1111/j.1539-6924.2009.01337.x 20030766

[30] Gosling J. Methods for eliciting expert opinion to inform health technology assessment. 2014 Available from: https://pdfs.semanticscholar.org/38eb/a762cdaf5d6dae2fee2063bf776d5facec5b.pdf

[31] Arkes HR. Costs and benefits of judgment errors—implications for debiasing. Psychol Bull. 1991;110(3):486–98.

[32] Sellier AL Scopelliti I Morewedge CK. Debiasing training improves decision making in the field. Psychol Sci. 2019;30(9):1371–9. 10.1177/0956797619861429 31347444

[33] Winman A Hansson P Juslin P. Subjective probability intervals: how to reduce overconfidence by interval evaluation. J Exp Psychol Learn. 2004;30(6):1167–75. 10.1037/0278-7393.30.6.1167 15521796

[34] Microsoft. Microsoft Office Excel [computer software]. Redmond, WA: Microsoft; 2010.

[35] Winkler RL. Assessment of prior distributions in Bayesian analysis. J Am Stat Assoc. 1967;62(319):776–800.

[36] Dietrich F List C. Probabilistic opinion pooling. 2014 Available from: http://personal.lse.ac.uk/list/PDF-files/OpinionPoolingReview.pdf

