# Supplementary material for: Health Opportunity Costs: Assessing the Implications of Uncertainty Using Elicitation Methods with Experts
Source: Med Decis Making. 2020 May 22;40(4):448–59. doi: 10.1177/0272989X20916450 (PMC7509606; doi:10.1177/0272989X20916450)
Supplement: Manuscript_expert_elicitation_HOC_MDM_4_Appendix1_online_supp – Supplemental material for Health Opportunity Costs: Assessing the Implications of Uncertainty Using Elicitation Methods with Experts [file Manuscript_expert_elicitation_HOC_MDM_4_Appendix1_online_supp.pdf]

Health opportunity costs in the NHS: assessing the implications of uncertainty using elicitation methods with experts

Work funded by the Department of Health's Policy Research Unit in Economic Evaluation of Health and Care Interventions

Context: Decisions about health care interventions in the UK consider not only the expected benefit associated with each intervention, but also the consequences of any additional costs imposed to the NHS. These 'opportunity costs' are incurred in various ways. For example, if new investments are being considered that impose additional costs in the NHS, other patient services and activities can be expected to be affected, with implications for their health outcomes.

A recent MRC and NIHR-funded research project, using routinely available data, aimed to provide an empirical basis for the estimate of expected opportunity costs.[1] Programme budgeting data was used to quantify NHS expenditure, which considers the costs of activities such as prevention and health promotion, primary care services and prescribing, inpatient and outpatient activities including treatment, diagnostic, other medical devices and other services (such as mental health services), ambulance and A&E, community care and social care activities. The study quantified how changes in the NHS expenditure in a given year are allocated between disease areas, and how changes in expenditure by disease area in a particular year affect the rates of disease-specific mortality in that year.

However, the implications of changes in NHS expenditure cannot be assumed only to relate to an effect on mortality in the same year of expenditure. There should also be consideration of (A) the possibility of changes in expenditure having effects beyond the year of expenditure; (B) how the effects of changes in expenditure on mortality relate to effects on a broader measure of health that incorporates both duration and quality of life impacts (life years adjusted for quality); (C) how changes in expenditure affect health in disease areas for which previous work[1] could not measure a mortality effect; and (D) the additional life-years associated with any reduction in the rate of mortality. There are only very limited data available, if any, on each of these items. To help to establish which assumptions are the most appropriate, this questionnaire aims to elicit your beliefs over these important uncertainties.

[1] Claxton et al. Methods for the Estimation of the NICE Cost Effectiveness Threshold. HTA. 2015;19(14):503.

The exercise: The questions are difficult, and they will require some careful consideration. There are no right or wrong answers and we do not expect you to provide definitive answers. On the contrary, you will be asked to think honestly about the uncertainty in your answer to each question and to express how uncertain you are about it in your responses.

Your contribution: You will be asked to give your opinion individually (not in groups). The information provided, including any personal details, will be kept anonymous and confidential, stored securely, and only accessed by those carrying out the study.

Health opportunity costs in the NHS: assessing the implications of uncertainty using elicitation methods with experts

Work funded by the Department of Health's Policy Research Unit in Economic Evaluation of Health and Care Interventions

About you

1. Please choose the option(s) that best describe the policy organisation(s) of which you are part:

I have no involvement in policy ☐

Governmental Bodies (DH, NHSE, PHE) ☐

Non Departmental Public Bodies and Independent Departmental Expert Committees (NICE, JCVI) ☐

Industry-related bodies (ABPI) ☐

Patient representative organisations ☐

Other (please specify): ____________________

2. Please choose the option(s) that best describe the clinical area(s) in which you are specialised:

I have no clinical expertise ☐

Circulatory ☐

Respiratory ☐

Gastrointestinal ☐

Neurological ☐

Mental health ☐

Endocrinology ☐

Musculoskeletal ☐

Primary care ☐

Other clinical area (please specify): ____________________

Questionnaire

This questionnaire focusses on the effects to population health of changes to NHS expenditure in a particular year (all else remaining unchanged). These changes may be increases or decreases in expenditure but, for clarity, throughout this questionnaire we will refer to increases in expenditure. We would like you to think of changes in expenditure that are significant, but still represent a small proportion of NHS' expenditure.

We will ask you to consider broad disease areas such as 'problems of the respiratory system' or 'problems of the musculoskeletal system'. Each disease area is broad and in Appendix 1 you will find the ICD (International Classification of Diseases) codes that define them. These may be very heterogeneous. Therefore, you may think of a specific condition/intervention when you first work through each section, but the answers we need should consider the ICDs where an increase in expenditure is more likely to fall.

Section A

If, in a particular year, NHS expenditure is increased for a specific disease area (all else remaining unchanged) individuals in that disease area are expected to face lower disease-related mortality rates in that same year. Increasing expenditure in a particular year may, however, also affect the disease-specific mortality rates in subsequent years (of those same individuals or other individuals being cared for in the future). The following questions aim to ask about your beliefs on the effects in subsequent years.

A1. On average, for how many more years (beyond the year of increased expenditure) would you expect disease-specific mortality rates to be reduced in each of the specific disease areas listed below?

Please specify values equal to or bigger than 0.
If you cannot distinguish between two or more disease areas please repeat the same values

Please specify values equal to or bigger than 0.	My best guess for the value of this quantity is:	I am very certain (90% certain) that the true value for this quantity is … … higher than:	… lower than:
Consider each of the following disease areas:			
Circulatory	_______ years	_______ years	_______ years
Respiratory	_______ years	_______ years	_______ years
Gastrointestinal	_______ years	_______ years	_______ years
Neurological	_______ years	_______ years	_______ years
Endocrinology	_______ years	_______ years	_______ years
Cancers and Tumours; Problems of the genitourinary system; Infectious diseases; Problems due to trauma and injuries; Maternity and reproductive health; Conditions of neonates	_______ years	_______ years	_______ years

Refer to Appendix 1 for a detailed list of ICD10 codes included in each disease area

A2. Consider the reduction in mortality rate in the first year as a proportion of the total mortality rate associated with disease in that same year (the proportionate reduction). This question asks you about your beliefs on the magnitude of reductions in mortality in subsequent years. You are asked to express these as a proportion of the effect in the 1st year (guidance is provided in the bullet points below). For simplicity, we cover only the 2nd, 3rd and 4th years; however, we acknowledge that you might have indicated a longer duration of effects in question A1 and we will take full consideration of this in analyses.

From an increase in expenditure in a particular year, how do reductions in mortality rates in subsequent years compare to the reduction observed in the first year?

. A value of 0 indicates no reduction in mortality expected in the second/third/fourth year
. A value of 0.5 indicates the effects in the 2nd year to be half the effects in the first year. For example, if we assume a reduction in mortality of 5% in the first year, then in the 2nd year we would expect a reduction of 2.5% (5% x 0.5).
. A value of 1 indicates the effects in the 2nd year to be equal to those in the first year
. A value of 1.5 would indicate the reduction in the 2nd year to be 1.5 times that of the first year. For example, if we assume a reduction of 5% in the first year, then in the 2nd year you would expect a reduction of 7.5% (5% x 1.5).
If you cannot distinguish between two or more disease areas please repeat the same values

	My best guess for the value of this quantity is:	I am very certain (90% certain) that the true value for this quantity is … … higher than:	… lower than:
Circulatory			
2nd year, in relation to the 1st year:	_________	_________	_________
3rd year, in relation to the 1st year:	_________	_________	_________
4th year, in relation to the 1st year:	_________	_________	_________
Respiratory			
2nd year, in relation to the 1st year:	_________	_________	_________
3rd year, in relation to the 1st year:	_________	_________	_________
4th year, in relation to the 1st year:	_________	_________	_________
Gastrointestinal			
2nd year, in relation to the 1st year:	_________	_________	_________
3rd year, in relation to the 1st year:	_________	_________	_________
4th year, in relation to the 1st year:	_________	_________	_________
Neurological			
2nd year, in relation to the 1st year:	_________	_________	_________
3rd year, in relation to the 1st year:	_________	_________	_________
4th year, in relation to the 1st year:	_________	_________	_________
Endocrinology			
2nd year, in relation to the 1st year:	_________	_________	_________
3rd year, in relation to the 1st year:	_________	_________	_________
4th year, in relation to the 1st year:	_________	_________	_________
Cancers and Tumours; Problems of the genitourinary system; Infectious diseases; Problems due to trauma and injuries; Maternity and reproductive health; Conditions of neonates			
2nd year, in relation to the 1st year:	_________	_________	_________
3rd year, in relation to the 1st year:	_________	_________	_________
4th year, in relation to the 1st year:	_________	_________	_________

Refer to Appendix 1 for a detailed list of ICD10 codes included in each disease area

A3. Are you confident the answers you gave to questions A1 and A2 reflect your views and uncertainties?

YES NOT SURE NO (circle your response).

If you responded NOT SURE or NO, please give us some more detail as to why:

____

____

____

____

____

____

____

____

____

____

____

____

Please stop here and wait for the next presentation

Reminder: This questionnaire focusses on the effects to population health of changes to NHS expenditure in a particular year (all else remaining unchanged). We would like you to think of changes in expenditure that are significant, but still represent a small proportion of NHS expenditure.

We will ask you to consider broad disease areas (see Appendix 1 for a list of the ICD codes that define them). The answers we need should consider the ICDs within a disease area where an increase in expenditure is more likely to fall.

Section B.

The previous section concerned the expected effects of increased expenditure on mortality rates. We expressed these mortality effects as proportionate reductions in the total rates of mortality associated with particular diseases, which can be thought of as reductions in *mortality burden*. But there is a broader *health burden* to disease that considers its impact on both the rate of mortality (and any life-years lost as a consequence) and on the level of health-related quality of life of individuals. Such measure of burden can be described as per patient quality-adjusted life-years lost due to disease.

B1. This question asks about your beliefs on how the effects of increased expenditure on health burden compare with its effects on mortality burden. In other words, if expenditure is increased in a particular year, how many times bigger (or smaller) are proportionate reductions on quality-adjusted life year burden when compared to proportionate reductions on mortality burden?

We will ask you to indicate your beliefs on this relationship for the year of increased expenditure (1st year) and also for any later effects of expenditure on the 2nd, 3rd and 4th years subsequent to increased expenditure. When asked about the 2nd year, for example, compare the effects on health burden in the 2nd year following increased expenditure with effects on mortality burden in the 2nd year following increased expenditure (guidance is provided in the bullet points below). When thinking of effects on health burden in a particular year, you should not only consider reductions in mortality burden in that year, and future life-years saved as a consequence of these mortality effects, but also effects on quality of life in that year.

How do the effects of increased expenditure on health burden compare with its effects on mortality burden?

Please specify values equal to or bigger than 0

- *a value of zero indicates no reduction in health burden*
- *a value of 1 indicates that the proportionate reduction in health burden is the same as that in mortality burden*
- *values between zero and 1 indicate that the proportionate effect of increased expenditure on health burden is less than that on mortality burden. For example, a value of 0.5 indicates that increased expenditure is expected to produce half the reduction in health burden than that it produces on mortality burden*
- *values higher than 1 indicate that the proportionate effect of increased expenditure on health burden is more than that on mortality burden. For example, a value of 1.5 indicates that increased expenditure is expected to produce 1.5 times the reduction in health burden than that it produces on mortality burden*

If you cannot distinguish between two or more disease areas please repeat the same values

Please specify values equal to or bigger than 0.	My best guess for the value of this quantity is:	I am very certain (90% certain) that the true value for this quantity is … … higher than:	… lower than:
Circulatory			
1st year (the year of expenditure)	________	________	________
2nd year	________	________	________
3rd year	________	________	________
4th year	________	________	________
Respiratory			
1st year (the year of expenditure)	________	________	________
2nd year	________	________	________
3rd year	________	________	________
4th year	________	________	________
Gastrointestinal			
1st year (the year of expenditure)	________	________	________
2nd year	________	________	________
3rd year	________	________	________
4th year	________	________	________
Neurological			
1st year (the year of expenditure)	________	________	________
2nd year	________	________	________
3rd year	________	________	________
4th year	________	________	________
Endocrinology			
1st year (the year of expenditure)	________	________	________
2nd year	________	________	________
3rd year	________	________	________
4th year	________	________	________
Cancers and Tumours, Problems of the genitourinary system, Infectious diseases, Problems due to trauma and injuries, Maternity and reproductive health, Conditions of neonates			
1st year (the year of expenditure)	________	________	________
2nd year	________	________	________
3rd year	________	________	________
4th year	________	________	________

Refer to Appendix 1 for a detailed list of ICD10 codes included in each disease area

B2. Are you confident the answers you gave to question B1 reflect your views and uncertainties?

YES NOT SURE NO (circle your response).

If you responded NOT SURE or NO, please give us some more detail as to why:

____

____

____

____

____

____

____

____

____

____

____

____

Please stop here and wait for the next presentation

<u>Reminder:</u> This questionnaire focusses on the effects to population health of changes to NHS expenditure in a particular year (all else remaining unchanged). We would like you to think of changes in expenditure that are significant, but still represent a small proportion of NHS expenditure.

We will ask you to consider broad disease areas (see Appendix 1 for a list of the ICD codes that define them). The answers we need should consider the ICDs within a disease area where an increase in expenditure is more likely to fall.

Section C.

Previous research was able to identify a measurable effect of changes in expenditure on mortality rates for some disease areas; in others, such effects could not be measured:

The previous section asked you to consider how an increase in NHS expenditure would be expected to reduce the health burden (quality-adjusted life-years lost due to disease). It focussed on disease areas for which previous research was able to identify a measurable mortality effect from increased expenditure:

- Disease areas **with** measurable effects of changes in expenditure[1]: Problems of circulation; Problems of the respiratory system; Problems of the gastrointestinal system; Endocrine, nutritional and metabolic problems; Neurological; Cancers and Tumours; Problems of the genitourinary system; Infectious diseases; Problems due to trauma and injuries; Maternity and reproductive health; Conditions of neonates

In the previous section, you were asked to consider individual disease areas in this group; now we would like you to consider the average effect across all disease areas in this group.

This section asks about your beliefs regarding how these effects on health burden compare to those in specific disease areas that did not have measurable mortality effects. These are:

- Disease areas **without** measurable effects of changes in expenditure[1]: Mental Health Disorders; Problems of the Musculoskeletal system; Disorders of Blood; Problems of Learning Disability; Problems of Vision; Problems of Hearing; Dental problems; Problems of the Skin; Adverse events and poisoning; Other

Specifically, we ask how much bigger (or smaller) are reductions in health burden (quality-adjusted life-years) when expenditure is increased, for example, in 'mental health disorders' instead of disease areas with a measured effect of increased expenditure on mortality (average effect across all disease areas in this group).

Please indicate your beliefs on this relationship for the year of expenditure (1st year) and also for any later effects of expenditure on subsequent years (2nd, 3rd and 4th).

[1] Refer to Appendix 1 for a detailed list of ICD10 codes included in each disease area

C1. How do reductions in health burden (quality-adjusted life-years lost due to disease) from an increase in NHS expenditure in the following disease areas compare to reductions in health burden from increased expenditure across all disease areas with measurable mortality effects?

Please specify values equal to or bigger than 0

. a value of zero indicates that the specific disease area without measurable mortality effects from increased expenditure (e.g. mental health disorders) would not see health burden reduced from increased expenditure

. a value of 1 indicates that the effects on health burden of increased expenditure for the specific disease area under consideration (e.g. mental health disorders) would be equal to that observed across disease areas with measurable mortality effects from increased expenditure

. values between zero and 1 indicate that the proportionate effect of increased expenditure on health burden is expected to be less in the specific disease area being considered (e.g. mental health disorders) than in those with measurable mortality effects. For example, a value of 0.5 would indicate an expected reduction in health burden in 'mental health disorders', for example, of half that in disease areas with measurable mortality effects.

. values higher than 1 indicate that the proportionate effect of increased expenditure on health burden is expected to be more in the specific disease area being considered (e.g. mental health disorders) than in those with measurable mortality effects. For example, a value of 1.5 would indicate an expected reduction in health burden in 'mental health disorders' that is 1.5 times that in disease areas with measurable mortality effects.

If you cannot distinguish two or more disease areas please repeat the same values for them

Please specify values equal to or bigger than 0	My best guess for the value of this quantity is:	I am very certain (90% certain) that the true value for this quantity is …	
		… higher than:	… lower than:
Mental health disorders			
Think of the 1st year (year of expenditure):	_________	_________	_________
Think of the 2nd year	_________	_________	_________
Think of the 3rd year:	_________	_________	_________
Think of the 4th year:	_________	_________	_________
Problems of the Musculoskeletal system			
Think of the 1st year (year of expenditure):	_________	_________	_________
Think of the 2nd year	_________	_________	_________
Think of the 3rd year:	_________	_________	_________
Think of the 4th year:	_________	_________	_________
Disorders of Blood, Problems of Learning Disability, Problems of Vision, Problems of Hearing, Dental problems, Problems of the Skin, Adverse events and poisoning, Other			
Think of the 1st year (year of expenditure):	_________	_________	_________
Think of the 2nd year	_________	_________	_________
Think of the 3rd year:	_________	_________	_________
Think of the 4th year:	_________	_________	_________

Refer to Appendix 1 for a detailed list of ICD10 codes included in each disease area

C2. Are you confident the answers you gave to question C1 reflect your views and uncertainties?

YES NOT SURE NO (circle your response).

If you responded NOT SURE or NO, please give us some more detail as to why:

____

____

____

____

____

____

____

____

____

____

____

____

Please stop here and wait for the next presentation

Reminder: This questionnaire focusses on the effects to population health of changes to NHS expenditure in a particular year (all else remaining unchanged). We would like you to think of changes in expenditure that are significant, but still represent a small proportion of NHS expenditure.

We will ask you to consider broad disease areas (see Appendix 1 for a list of the ICD codes that define them). The answers we need should consider the ICDs within a disease area where an increase in expenditure is more likely to fall.

Section D

Previous work used available data to evaluate the expected reduction in mortality rates in a particular year from an increase in NHS expenditure in that same year[2]. The way in which this mortality effect was estimated means that some of the deaths identified as being averted in the spend year were averted for at least 3 years, others for at least 2 years and others for at least 1 year. From the available data, we could not evaluate for how much longer each of these deaths was averted. This is what the next two questions focus on. This is only applicable to disease areas for which changes in expenditure have had measurable mortality effects.

[2] Claxton et al. Methods for the Estimation of the NICE Cost Effectiveness Threshold. HTA. 2015;19(14):503.

D1. Of those patients that have seen their deaths averted by at least 3 years, 2 years and 1 year, what proportion are likely to return to (or exceed) the life expectancy of the general population of the same age and gender?

Please specify values between 0 and 1, where 0 represents that no patient will get back to, or exceed, the life expectancy of the general population of the same age and gender, and 1 that all patients will get back to the life expectancy of the general population of the same age and gender.

Please specify values between 0 and 1		My best guess for the value of this quantity is:	I am very certain (90% certain) that the true value for this quantity is … … higher than:	… lower than:
Circulatory				
For patients that had death averted for at least:	3 years	_________	_________	_________
	2 years	_________	_________	_________
	1 year	_________	_________	_________
Respiratory				
For patients that had death averted for at least:	3 years	_________	_________	_________
	2 years	_________	_________	_________
	1 year	_________	_________	_________
Gastrointestinal				
For patients that had death averted for at least:	3 years	_________	_________	_________
	2 years	_________	_________	_________
	1 year	_________	_________	_________
Neurological				
For patients that had death averted for at least:	3 years	_________	_________	_________
	2 years	_________	_________	_________
	1 year	_________	_________	_________
Endocrinology				
For patients that had death averted for at least:	3 years	_________	_________	_________
	2 years	_________	_________	_________
	1 year	_________	_________	_________
Cancers and Tumours; Problems of the genitourinary system; Infectious diseases; Problems due to trauma and injuries; Maternity and reproductive health; Conditions of neonates				
For patients that had death averted for at least:	3 years	_________	_________	_________
	2 years	_________	_________	_________
	1 year	_________	_________	_________

Refer to Appendix 1 for a detailed list of ICD10 codes included in each disease area

D2. Now consider only those patients who have <u>not</u> returned to, or exceeded, normal life expectancy. Please report your beliefs on their life expectancy as a proportion of the life expectancy in the general population of the same age and gender:

Please specify values between 0 and 1. Note that 0 represents immediate death and 1 the life expectancy of the general population of the same age and gender. For example, a value of 0.5 indicates that a patient in that disease area whose death was averted by an increase in expenditure, but who does not return to normal life expectancy, would have half the life expectancy of the general population of the same age and gender.

Please specify values between 0 and 1.		My best guess for the value of this quantity is:	I am very certain (90% certain) that the true value for this quantity is …	
			… higher than:	… lower than:
Circulatory				
For patients that had death averted for at least:	3 years	_________	_________	_________
	2 years	_________	_________	_________
	1 year	_________	_________	_________
Respiratory				
For patients that had death averted for at least:	3 years	_________	_________	_________
	2 years	_________	_________	_________
	1 year	_________	_________	_________
Gastrointestinal				
For patients that had death averted for at least:	3 years	_________	_________	_________
	2 years	_________	_________	_________
	1 year	_________	_________	_________
Neurological				
For patients that had death averted for at least:	3 years	_________	_________	_________
	2 years	_________	_________	_________
	1 year	_________	_________	_________
Endocrinology				
For patients that had death averted for at least:	3 years	_________	_________	_________
	2 years	_________	_________	_________
	1 year	_________	_________	_________
Cancers and Tumours; Problems of the genitourinary system; Infectious diseases; Problems due to trauma and injuries; Maternity and reproductive health; Conditions of neonates				
For patients that had death averted for at least:	3 years	_________	_________	_________
	2 years	_________	_________	_________
	1 year	_________	_________	_________

Refer to Appendix 1 for a detailed list of ICD10 codes included in each disease area

D3. Are you confident the answers you gave to questions D1 and D2 reflect your views and uncertainties?

YES NOT SURE NO (circle your response).

If you responded NOT SURE or NO, please give us some more detail as to why:

____

____

____

____

____

____

____

____

____

____

____

____

This was the last set of questions.

Now please take some time to look through your answers to questions in sections A to D again and revise them if needed

E. Comments and feedback

E1. If you have any comments about any aspects of today please add them here.

____

____

____

____

____

____

____

____

____

____

____

____

____

____

____

____

Appendix 1 – Full list of diseases considered (based on ICD-10 3-digit codes) with each disease area

Disease areas with measurable mortality effects from changes in expenditure

Problems of circulation: Rheumatic fever without mention of heart involvement; Rheumatic fever with heart involvement; Rheumatic chorea; Rheumatic mitral valve diseases; Rheumatic aortic valve diseases; Rheumatic tricuspid valve diseases; Multiple valve diseases; Other rheumatic heart diseases; Essential (primary) hypertension; Hypertensive heart disease; Hypertensive heart and renal disease; Secondary hypertension; Angina pectoris; Acute myocardial infarction; Subsequent myocardial infarction; Other acute ischemic heart diseases; Chronic ischemic heart disease; Pulmonary embolism; Other pulmonary heart diseases; Other diseases of pulmonary vessels; Acute pericarditis; Other diseases of pericardium; Acute and subacute endocarditis; Nonrheumatic mitral valve disorders; Nonrheumatic aortic valve disorders; Nonrheumatic tricuspid valve disorders; Pulmonary valve disorders; Endocarditis, valve unspecified; Acute myocarditis; Cardiomyopathy; Atrioventricular and left bundle-branch block; Other conduction disorders; Cardiac arrest; Paroxysmal tachycardia; Atrial fibrillation and flutter; Other cardiac arrhythmias; Heart failure; Complications and ill-defined descriptions of heart disease; Subarachnoid hemorrhage; Intracerebral hemorrhage; Other nontraumatic intracranial hemorrhage; Cerebral infarction; Stroke, not specified as hemorrhage or infarction; Other cerebrovascular diseases; Atherosclerosis; Aortic aneurysm and dissection; Other aneurysm; Other peripheral vascular diseases; Arterial embolism and thrombosis; Other disorders of arteries and arterioles; Diseases of capillaries; Phlebitis and thrombophlebitis; Portal vein thrombosis; Other venous embolism and thrombosis; Varicose veins of lower extremities; Varicose veins of other sites; Other disorders of veins; Nonspecific lymphadenitis; Other noninfective disorders of lymphatic vessels and lymph nodes; Hypotension; Postprocedural disorders of circulatory system, not elsewhere classified ; Other and unspecified disorders of circulatory system; Congenital malformations of cardiac chambers and connections; Congenital malformations of cardiac septa; Congenital malformations of pulmonary and tricuspid valves; Congenital malformations of aortic and mitral valves; Other congenital malformations of heart; Congenital malformations of great arteries; Congenital malformations of great veins; Other congenital malformations of peripheral vascular system; Other congenital malformations of circulatory system; Chagas' disease; Abnormalities of heart beat; Cardiac murmurs and other cardiac sounds; Gangrene, not elsewhere classified; Abnormal blood-pressure reading, without diagnosis; Shock, not elsewhere classified

Problems of the respiratory system: Respiratory tuberculosis, not confirmed bacteriologically or histologically; Miliary tuberculosis; Infection due to other mycobacteria; Diphtheria; Whooping cough; Actinomycosis; Nocardiosis; Chlamydia psittaci infection; Cytomegaloviral disease; Coccidioidomycosis; Histoplasmosis; Blastomycosis; Paracoccidioidomycosis; Sporotrichosis; Aspergillosis; Cryptococcosis; Zygomycosis; Acute nasopharyngitis [common cold]; Acute sinusitis; Acute pharyngitis; Acute tonsillitis; Acute laryngitis and tracheitis; Acute obstructive laryngitis [croup] and epiglottitis; Acute upper respiratory infections of multiple and unspecified sites; Influenza due to identified influenza virus; Influenza, virus not identified; Viral pneumonia, not elsewhere classified; Pneumonia due to Streptococcus pneumoniae; Pneumonia due to Hemophilus influenzae; Bacterial pneumonia, not elsewhere classified; Pneumonia due to other infectious organisms, not elsewhere classified; Pneumonia, organism unspecified; Acute bronchitis; Acute bronchiolitis; Unspecified acute lower respiratory infection; Vasomotor and allergic rhinitis; Chronic rhinitis, nasopharyngitis, and pharyngitis; Chronic sinusitis; Nasal polyp; Other disorders of nose and nasal sinuses; Chronic diseases of tonsils and adenoids; Peritonsillar abscess; Chronic laryngitis and laryngotracheitis; Diseases of vocal cords and larynx, not elsewhere classified; Other diseases of upper respiratory tract; Bronchitis, not specified as acute or chronic; Simple and mucopurulent chronic bronchitis; Unspecified chronic bronchitis; Emphysema; Other chronic obstructive pulmonary disease; Asthma; Status asthmaticus; Bronchiectasis; Coalworker's pneumoconiosis; Pneumoconiosis due to asbestos and other mineral fibers; Pneumoconiosis due to dust containing silica; Pneumoconiosis due to other inorganic dusts; Unspecified pneumoconiosis; Pneumoconiosis associated with tuberculosis; Airway disease due to specific organic dust; Hypersensitivity pneumonitis due to organic dust; Respiratory conditions due to inhalation of chemicals, gases, fumes, and vapors; Pneumonitis due to solids and liquids; Respiratory conditions due to other external agents; Adult respiratory distress syndrome; Pulmonary edema; Pulmonary eosinophilia, not elsewhere classified; Other interstitial pulmonary diseases; Abscess of lung and mediastinum; Pyothorax; Pleural effusion, not elsewhere classified; Pleural plaque; Pneumothorax; Other pleural conditions; Postprocedural respiratory disorders, not elsewhere classified;

Respiratory failure, not elsewhere classified; Other respiratory disorders; Congenital malformations of nose; Congenital malformations of larynx; Congenital malformations of trachea and bronchus; Congenital malformations of lung; Other congenital malformations of respiratory system; Hemorrhage from respiratory passages; Cough; Abnormalities of breathing; Other symptoms and signs involving the circulatory and respiratory systems; Abnormal findings in specimens from respiratory organs and thorax; Abnormal findings on diagnostic imaging of lung

Problems of the gastrointestinal system: Cholera; Typhoid and paratyphoid fevers; Other salmonella infections; Shigellosis; Other bacterial intestinal infections; Other bacterial foodborne intoxications, not elsewhere classified; Amebiasis; Other protozoal intestinal diseases; Viral and other specified intestinal infections; Diarrhea and gastroenteritis of infectious origin; Leptospirosis; Other spirochetal infections; Yellow fever; Acute hepatitis A; Acute hepatitis B; Other acute viral hepatitis; Chronic viral hepatitis; Unspecified viral hepatitis; Candidiasis; Echinococcosis; Teniasis; Diphyllobothriasis and sparganosis; Other cestode infections; Trichinellosis; Strongyloidiasis; Enterobiasis; Other intestinal helminthiases, not elsewhere classified; Unspecified intestinal parasitism; Other helminthiases; Hemorrhoids; Esophageal varices; Diseases of salivary glands; Stomatitis and related lesions; Other diseases of lip and oral mucosa; Diseases of tongue; Esophagitis; Gastroesophageal reflux disease; Other diseases of esophagus; Gastric ulcer; Duodenal ulcer ; Peptic ulcer, site unspecified ; Gastrojejunal ulcer ; Gastritis and duodenitis; Dyspepsia; Other diseases of stomach and duodenum; Acute appendicitis; Other appendicitis; Unspecified appendicitis; Other diseases of appendix; Inguinal hernia; Femoral hernia; Umbilical hernia; Ventral hernia; Diaphragmatic hernia; Other abdominal hernia; Unspecified abdominal hernia; Crohn's disease [regional enteritis]; Ulcerative colitis; Other noninfective gastroenteritis and colitis; Vascular disorders of intestine; Paralytic ileus and intestinal obstruction without hernia; Diverticular disease of intestine; Irritable bowel syndrome; Other functional intestinal disorders; Fissure and fistula of anal and rectal regions; Abscess of anal and rectal regions; Other diseases of anus and rectum; Other diseases of intestine; Peritonitis; Other disorders of peritoneum; Alcoholic liver disease; Toxic liver disease; Hepatic failure, not elsewhere classified; Chronic hepatitis, not elsewhere classified; Fibrosis and cirrhosis of liver; Other inflammatory liver diseases; Other diseases of liver; Cholelithiasis; Cholecystitis; Other diseases of gallbladder; Other diseases of biliary tract; Acute pancreatitis; Other diseases of pancreas; Intestinal malabsorption; Other diseases of digestive system; Cleft palate; Cleft lip; Cleft palate with cleft lip; Other congenital malformations of tongue, mouth and pharynx; Congenital malformations of esophagus; Other congenital malformations of upper alimentary tract; Congenital absence, atresia, and stenosis of small intestine; Congenital absence, atresia, and stenosis of large intestine; Other congenital malformations of intestine; Congenital malformations of gallbladder, bile ducts, and liver; Other congenital malformations of digestive system; Hookworm diseases; Ascariasis; Trichuriasis; Abdominal and pelvic pain; Nausea and vomiting; Heartburn; Dysphagia; Flatulence and related conditions; Fecal incontinence; Hepatomegaly and splenomegaly, not elsewhere classified; Unspecified jaundice; Ascites; Other symptoms and signs involving the digestive system and abdomen; Abnormal findings in specimens from digestive organs and abdominal cavity

Endocrine, nutritional and metabolic problems: Congenital iodine-deficiency syndrome; Iodine-deficiency-related thyroid disorders and allied conditions; Subclinical iodine-deficiency hypothyroidism; Other hypothyroidism; Other nontoxic goiter; Thyrotoxicosis [hyperthyroidism]; Thyroiditis; Other disorders of thyroid; Insulin-dependent diabetes mellitus; Non-insulin-dependent diabetes mellitus ; Malnutrition-related diabetes mellitus ; Other specified diabetes mellitus ; Unspecified diabetes mellitus ; Nondiabetic hypoglycemic coma; Other disorders of pancreatic internal secretion; Hypoparathyroidism; Hyperparathyroidism and other disorders of parathyroid gland; Hyperfunction of pituitary gland; Hypofunction and other disorders of the pituitary gland; Cushing's syndrome; Adrenogenital disorders; Hyperaldosteronism; Other disorders of adrenal gland; Ovarian dysfunction; Testicular dysfunction; Disorders of puberty, not elsewhere classified; Polyglandular dysfunction; Diseases of thymus; Other endocrine disorders; Kwashiorkor; Nutritional marasmus; Marasmic kwashiorkor; Unspecified severe protein-energy malnutrition; Protein-energy malnutrition of moderate and mild degree; Retarded development following protein-energy malnutrition; Unspecified protein-energy malnutrition; Thiamin deficiency; Niacin deficiency [pellagra]; Deficiency of other B group vitamins; Ascorbic acid deficiency; Vitamin D deficiency; Other vitamin deficiencies; Dietary calcium deficiency; Dietary selenium deficiency; Dietary zinc deficiency; Deficiency of other nutrient elements; Other nutritional deficiencies; Sequelae of malnutrition and other nutritional deficiencies; Other hyperalimentation; Sequelae of hyperalimentation; Disorders of aromatic amino-acid metabolism; Disorders of branched-chain amino-acid metabolism and fatty-acid metabolism; Other disorders of amino-acid metabolism; Lactose intolerance; Other disorders of carbohydrate metabolism; Disorders of sphingolipid metabolism and other lipid storage disorders; Disorders of glycosaminoglycan metabolism; Disorders of glycoprotein metabolism; Disorders of purine and pyrimidine metabolism; Disorders of porphyrin and bilirubin metabolism; Disorders of mineral metabolism; Cystic fibrosis; Amyloidosis; Volume depletion; Other disorders of fluid, electrolyte, and acid-base balance; Other metabolic disorders; Postprocedural endocrine and

metabolic disorders, not elsewhere classified; Vitamin A deficiency; Symptoms and signs concerning food and fluid intake; Cachexia; Other general symptoms and signs; Elevated blood glucose level; Abnormal serum enzyme levels; Other abnormal findings of blood chemistry; Glycosuria

Neurological: Tuberculosis of nervous system; Meningococcal infection; Acute poliomyelitis; Atypical virus infections of central nervous system; Rabies; Mosquito-borne viral encephalitis; Tick-borne viral encephalitis; Other viral encephalitis, not elsewhere classified; Unspecified viral encephalitis; Viral meningitis; Unspecified viral infection of central nervous system; Varicella [chickenpox]; Zoster [herpes zoster]; Measles; Rubella [German measles]; Other viral diseases, not elsewhere classified; Plasmodium falciparum malaria; Cysticercosis; Bacterial meningitis, not elsewhere classified; Meningitis due to other and unspecified causes; Encephalitis, myelitis, and encephalomyelitis; Intracranial and intraspinal abscess and granuloma; Intracranial and intraspinal phlebitis and thrombophlebitis; Sequelae of inflammatory diseases of central nervous system; Huntington's disease; Hereditary ataxia; Spinal muscular atrophy and related syndromes; Parkinson's disease; Secondary parkinsonism; Other degenerative diseases of basal ganglia; Dystonia; Other extrapyramidal and movement disorders; Other degenerative diseases of nervous system, not elsewhere classified; Multiple sclerosis; Other acute disseminated demyelination; Other demyelinating diseases of central nervous system; Epilepsy; Status epilepticus; Other headache syndromes; Transient cerebral ischemic attacks and related syndromes; Sleep disorders; Disorders of trigeminal nerve; Facial nerve disorders; Disorders of other cranial nerves; Nerve root and plexus disorders; Mononeuropathies of upper limb; Mononeuropathies of lower limb; Other mononeuropathies; Hereditary and idiopathic neuropathy; Inflammatory polyneuropathy; Other polyneuropathies; Other disorders of peripheral nervous system; Myasthenia gravis and other myoneural disorders; Primary disorders of muscles; Other myopathies; Infantile cerebral palsy; Hemiplegia; Paraplegia and tetraplegia; Other paralytic syndromes; Disorders of autonomic nervous system; Hydrocephalus; Toxic encephalopathy; Other disorders of brain; Other diseases of spinal cord; Other disorders of central nervous system; Postprocedural disorders of nervous system, not elsewhere classified; Other disorders of nervous system, not elsewhere classified; Otalgia and effusion of ear; Pain and other conditions associated with female genital organs and menstrual cycle; Anencephaly and similar malformations; Encephalocele; Microcephaly; Congenital hydrocephalus; Other congenital malformations of brain; Spina bifida; Other congenital malformations of spinal cord; Other congenital malformations of nervous system; Migraine; Pain in throat and chest; Disturbances of skin sensation; Abnormal involuntary movements; Abnormalities of gait and mobility; Other lack of coordination; Other symptoms and signs involving the nervous and musculoskeletal systems; Pain associated with micturition; Somnolence, stupor, and coma; Other symptoms and signs involving cognitive functions and awareness; Dizziness and giddiness; Disturbances of smell and taste; Other symptoms and signs involving general sensations and perceptions; Symptoms and signs involving emotional state; Symptoms and signs involving appearance and behavior; Speech disturbances, not elsewhere classified; Dyslexia and other symbolic dysfunctions, not elsewhere classified; Voice disturbances; Headache; Pain, not elsewhere classified; Syncope and collapse; Convulsions, not elsewhere classified; Hyperhidrosis; Abnormal findings in cerebrospinal fluid; Abnormal findings on diagnostic imaging of central nervous system; Abnormal findings on diagnostic imaging of other body structures; Abnormal results of function studies

Others:

Cancers and Tumours: Malignant neoplasm of lip; Malignant neoplasm of base of tongue; Malignant neoplasm of other and unspecified parts of tongue; Malignant neoplasm of gum; Malignant neoplasm of floor of mouth; Malignant neoplasm of palate; Malignant neoplasm of other and unspecified parts of mouth; Malignant neoplasm of parotid gland; Malignant neoplasm of other and unspecified major salivary glands; Malignant neoplasm of tonsil; Malignant neoplasm of oropharynx; Malignant neoplasm of nasopharynx; Malignant neoplasm of pyriform sinus; Malignant neoplasm of hypopharynx; Malignant neoplasm of other and ill-defined sites in the lip, oral cavity, and pharynx; Malignant neoplasm of esophagus; Malignant neoplasm of stomach; Malignant neoplasm of small intestine; Malignant neoplasm of colon; Malignant neoplasm of rectosigmoid junction; Malignant neoplasm of rectum; Malignant neoplasm of anus and anal canal; Malignant neoplasm of liver and intrahepatic bile ducts; Malignant neoplasm of gallbladder; Malignant neoplasm of other and unspecified parts of biliary tract; Malignant neoplasm of pancreas; Malignant neoplasm of other and ill-defined digestive organs; Malignant neoplasm of nasal cavity and middle ear; Malignant neoplasm of accessory sinuses; Malignant neoplasm of larynx; Malignant neoplasm of trachea; Malignant neoplasm of bronchus and lung; Malignant neoplasm of thymus; Malignant neoplasm of heart, mediastinum, and pleura; Malignant neoplasm of other and ill-defined sites in the respiratory system and intrathoracic organs; Malignant neoplasm of bone and articular cartilage of limbs;

Malignant neoplasm of bone and articular cartilage of other and unspecified sites; Malignant melanoma of skin; Other malignant neoplasms of skin; Mesothelioma; Kaposi's sarcoma; Malignant neoplasm of peripheral nerves and autonomic nervous system; Malignant neoplasm of retroperitoneum and peritoneum; Malignant neoplasm of other connective and soft tissue; Malignant neoplasm of breast; Malignant neoplasm of vulva; Malignant neoplasm of vagina; Malignant neoplasm of cervix uteri; Malignant neoplasm of corpus uteri; Malignant neoplasm of uterus, part unspecified; Malignant neoplasm of ovary; Malignant neoplasm of other and unspecified female genital organs; Malignant neoplasm of placenta; Malignant neoplasm of penis; Malignant neoplasm of prostate; Malignant neoplasm of testis; Malignant neoplasm of other and unspecified male genital organs; Malignant neoplasm of kidney, except renal pelvis; Malignant neoplasm of renal pelvis; Malignant neoplasm of ureter; Malignant neoplasm of bladder; Malignant neoplasm of other and unspecified urinary organs; Malignant neoplasm of eye and adnexa; Malignant neoplasm of meninges; Malignant neoplasm of brain; Malignant neoplasm of spinal cord, cranial nerves, and other parts of central nervous system; Malignant neoplasm of thyroid gland; Malignant neoplasm of adrenal gland; Malignant neoplasm of other endocrine glands and related structures; Malignant neoplasm of other and ill‑defined sites; Secondary and unspecified malignant neoplasm of lymph nodes; Secondary malignant neoplasm of respiratory and digestive organs; Secondary malignant neoplasm of other sites; Malignant neoplasm without specification of site; Hodgkin's disease; Follicular [nodular] non-Hodgkin's lymphoma; Diffuse non-Hodgkin's lymphoma; Peripheral and cutaneous T-cell lymphomas; Other and unspecified types of non-Hodgkin's lymphoma; Malignant immunoproliferative diseases; Multiple myeloma and malignant plasma cell neoplasms; Lymphoid leukemia; Myeloid leukemia; Monocytic leukemia; Other leukemias of specified cell type; Leukemia of unspecified cell type; Other and unspecified malignant neoplasms of lymphoid, hematopoietic, and related tissue; Malignant neoplasms of independent (primary) multiple sites; Carcinoma in situ of oral cavity, esophagus, and stomach; Carcinoma in situ of other and unspecified digestive organs; Carcinoma in situ of middle ear and respiratory system; Melanoma in situ; Carcinoma in situ of skin; Carcinoma in situ of breast; Carcinoma in situ of cervix uteri; Carcinoma in situ of other and unspecified genital organs; Carcinoma in situ of other and unspecified sites; Benign neoplasm of mouth and pharynx; Benign neoplasm of major salivary glands; Benign neoplasm of colon, rectum, anus, and anal canal; Benign neoplasm of other and ill-defined parts of digestive system; Benign neoplasm of middle ear and respiratory system; Benign neoplasm of other and unspecified intrathoracic organs; Benign neoplasm of bone and articular cartilage; Benign lipomatous neoplasm; Hemangioma and lymphangioma, any site; Benign neoplasm of mesothelial tissue; Benign neoplasm of soft tissue of retroperitoneum and peritoneum; Other benign neoplasms of connective and other soft tissue; Melanocytic nevi; Other benign neoplasms of skin; Benign neoplasm of breast; Leiomyoma of uterus; Other benign neoplasms of uterus; Benign neoplasm of ovary; Benign neoplasm of other and unspecified female genital organs; Benign neoplasm of male genital organs; Benign neoplasm of urinary organs; Benign neoplasm of eye and adnexa; Benign neoplasm of meninges; Benign neoplasm of brain and other parts of central nervous system; Benign neoplasm of thyroid gland; Benign neoplasm of other and unspecified endocrine glands; Benign neoplasm of other and unspecified sites; Neoplasm of uncertain or unknown behavior of oral cavity and digestive organs; Neoplasm of uncertain or unknown behavior of middle ear and respiratory and intrathoracic organs; Neoplasm of uncertain or unknown behavior of female genital organs; Neoplasm of uncertain or unknown behavior of male genital organs; Neoplasm of uncertain or unknown behavior of urinary organs; Neoplasm of uncertain or unknown behavior of meninges; Neoplasm of uncertain or unknown behavior of brain and central nervous system; Neoplasm of uncertain or unknown behavior of endocrine glands; Polycythemia vera; Myelodysplastic syndromes; Other neoplasms of uncertain or unknown behavior of lymphoid, hematopoietic, and related tissue; Neoplasm of uncertain or unknown behavior of other and unspecified sites; Benign mammary dysplasia; Unspecified lump in breast

Problems of the genitourinary system: Congenital syphilis; Early syphilis; Late syphilis; Other and unspecified syphilis; Gonococcal infection; Chancroid; Granuloma inguinale; Trichomoniasis; Anogenital herpesviral [herpes simplex] infection; Other predominantly sexually transmitted diseases, not elsewhere classified; Unspecified sexually transmitted disease; Mumps; Schistosomiasis [bilharziasis]; Hypertensive renal disease; Acute nephritic syndrome; Rapidly progressive nephritic syndrome ; Recurrent and persistent hematuria ; Chronic nephritic syndrome ; Nephrotic syndrome ; Unspecified nephritic syndrome ; Isolated proteinuria with specified morphological lesion ; Hereditary nephropathy, not elsewhere classified ; Acute tubulo-interstitial nephritis; Chronic tubulo-interstitial nephritis; Tubulo-interstitial nephritis, not specified as acute or chronic; Obstructive and reflux uropathy; Drug- and heavy-metal-induced tubulo-interstitial and tubular conditions; Other renal tubulo-interstitial diseases; Acute renal failure; Chronic renal failure; Unspecified renal failure; Calculus of kidney and ureter; Calculus of lower urinary tract; Unspecified renal colic; Disorders resulting from impaired renal tubular function; Unspecified contracted kidney; Small kidney of unknown cause; Other disorders of kidney and ureter, not elsewhere classified; Cystitis; Neuromuscular dysfunction of bladder, not elsewhere classified; Other disorders of bladder; Urethritis and urethral

syndrome; Urethral stricture; Other disorders of urethra; Other disorders of urinary system; Hyperplasia of prostate; Inflammatory diseases of prostate; Other disorders of prostate; Hydrocele and spermatocele; Torsion of testis; Orchitis and epididymitis; Redundant prepuce, phimosis, and paraphimosis; Other disorders of penis; Inflammatory disorders of male genital organs, not elsewhere classified; Other disorders of male genital organs; Salpingitis and oophoritis; Inflammatory disease of uterus, except cervix; Inflammatory disease of cervix uteri; Other female pelvic inflammatory diseases; Diseases of Bartholin's gland; Other inflammation of vagina and vulva; Endometriosis; Female genital prolapse; Fistulae involving female genital tract; Noninflammatory disorders of ovary, fallopian tube, and broad ligament; Polyp of female genital tract; Other noninflammatory disorders of uterus, except cervix; Erosion and ectropion of cervix uteri; Dysplasia of cervix uteri; Other noninflammatory disorders of cervix uteri; Other noninflammatory disorders of vagina; Other noninflammatory disorders of vulva and perineum; Absent, scanty, and rare menstruation; Excessive, frequent, and irregular menstruation; Other abnormal uterine and vaginal bleeding; Menopausal and other perimenopausal disorders; Congenital malformations of ovaries, fallopian tubes, and broad ligaments; Congenital malformations of uterus and cervix; Other congenital malformations of female genitalia; Undescended testicle; Hypospadias; Other congenital malformations of male genital organs; Indeterminate sex and pseudohermaphroditism; Renal agenesis and other reduction defects of kidney; Cystic kidney disease; Congenital obstructive defects of renal pelvis and congenital malformations of ureter; Other congenital malformations of kidney; Other congenital malformations of urinary system; Chlamydial lymphogranuloma (venereum); Other sexually transmitted chlamydial diseases; Unspecified hematuria; Unspecified urinary incontinence; Retention of urine; Anuria and oliguria; Polyuria; Urethral discharge; Other symptoms and signs involving the urinary system; Isolated proteinuria; Abnormal findings in specimens from male genital organs; Abnormal findings in specimens from female genital organs

Infectious diseases: Plague; Tularemia; Brucellosis; Glanders and melioidosis; Rat-bite fevers; Other zoonotic bacterial diseases, not elsewhere classified; Leprosy [Hansen's disease]; Other tetanus; Scarlet fever; Streptococcal septicemia; Other septicemia; Bartonellosis; Other bacterial diseases, not elsewhere classified; Bacterial infection of unspecified site; Nonvenereal syphilis; Pinta [carate]; Relapsing fevers; Typhus fever; Spotted fever [tick-borne rickettsioses]; Q fever; Other rickettsioses; Other viral infections of the central nervous system, not elsewhere classified; Other mosquito-borne viral fevers; Other arthropod-borne viral fevers, not elsewhere classified; Unspecified arthropod-borne viral fever; Arenaviral hemorrhagic fever; Other viral hemorrhagic fevers, not elsewhere classified; Unspecified viral hemorrhagic fever; Smallpox; Monkeypox; Unspecified viral infection characterized by skin and mucous membrane lesions; Human immunodeficiency virus [HIV] disease with infectious and parasitic diseases; Human immunodeficiency virus [HIV] disease with malignant neoplasms; Human immunodeficiency virus [HIV] disease with other specified diseases; Human immunodeficiency virus [HIV] disease with other conditions; Unspecified human immunodeficiency virus [HIV] disease; Infectious mononucleosis; Viral infection of unspecified site; Mycetoma; Other mycoses, not elsewhere classified; Unspecified mycosis; Plasmodium vivax malaria; Plasmodium malariae malaria; Other parasitologically confirmed malaria; Unspecified malaria; Leishmaniasis; Pneumocystosis; Other protozoal diseases, not elsewhere classified; Unspecified protozoal disease; Other fluke infections; Dracunculiasis; Other infestations; Unspecified parasitic disease; Other infectious diseases; Dengue fever [classical dengue]; Dengue hemorrhagic fever; African trypanosomiasis; Filariasis; Fever of unknown origin; Laboratory evidence of human immunodeficiency virus [HIV]

Problems due to trauma and injuries: Superficial injury of head; Open wound of head; Fracture of skull and facial bones; Dislocation, sprain, and strain of joints and ligaments of head; Injury of cranial nerves; Injury of eye and orbit; Intracranial injury; Crushing injury of head; Traumatic amputation of part of head; Other and unspecified injuries of head; Superficial injury of neck; Open wound of neck; Fracture of neck; Dislocation, sprain, and strain of joints and ligaments at neck level; Injury of nerves and spinal cord at neck level; Injury of blood vessels at neck level; Injury of muscle and tendon at neck level; Crushing injury of neck; Traumatic amputation at neck level; Other and unspecified injuries of neck; Superficial injury of thorax; Open wound of thorax; Fracture of rib(s), sternum, and thoracic spine; Dislocation, sprain, and strain of joints and ligaments of thorax; Injury of nerves and spinal cord at thorax level; Injury of blood vessels of thorax; Injury of heart; Injury of other and unspecified intrathoracic organs; Crushing injury of thorax and traumatic amputation of part of thorax; Other and unspecified injuries of thorax; Superficial injury of abdomen, lower back, and pelvis; Open wound of abdomen, lower back, and pelvis; Fracture of lumbar spine and pelvis; Dislocation, sprain, and strain of joints and ligaments of lumbar spine and pelvis; Injury of nerves and lumbar spinal cord at abdomen, lower back, and pelvis level; Injury of blood vessels at abdomen, lower back, and pelvis level; Injury of intra-abdominal organs; Injury of pelvic organs; Crushing injury and traumatic amputation of part of abdomen, lower back, and pelvis; Other and unspecified injuries of abdomen, lower back, and pelvis; Superficial injury of shoulder and upper arm; Open wound of shoulder and upper arm; Fracture of shoulder and upper arm; Dislocation, sprain, and strain of joints

and ligaments of shoulder girdle; Injury of nerves at shoulder and upper arm level; Injury of blood vessels at shoulder and upper arm level; Injury of muscle and tendon at shoulder and upper arm level; Crushing injury of shoulder and upper arm; Traumatic amputation of shoulder and upper arm; Other and unspecified injuries of shoulder and upper arm; Superficial injury of forearm; Open wound of elbow and forearm; Fracture of forearm; Dislocation, sprain, and strain of joints and ligaments of elbow; Injury of nerves at forearm level; Injury of blood vessels at forearm level; Injury of muscle and tendon at forearm level; Crushing injury of forearm; Traumatic amputation of forearm; Other and unspecified injuries of forearm; Superficial injury of wrist and hand; Open wound of wrist and hand; Fracture at wrist and hand level; Dislocation, sprain, and strain of joints and ligaments at wrist and hand level; Injury of nerves at wrist and hand level; Injury of blood vessels at wrist and hand level; Injury of muscle and tendon at wrist and hand level; Crushing injury of wrist and hand; Traumatic amputation of wrist and hand; Other and unspecified injuries of wrist and hand; Superficial injury of hip and thigh; Open wound of hip and thigh; Fracture of femur; Dislocation, sprain, and strain of joint and ligaments of hip; Injury of nerves at hip and thigh level; Injury of blood vessels at hip and thigh level; Injury of muscle and tendon at hip and thigh level; Crushing injury of hip and thigh; Traumatic amputation of hip and thigh; Other and unspecified injuries of hip and thigh; Superficial injury of lower leg; Open wound of lower leg; Fracture of lower leg, including ankle; Dislocation, sprain, and strain of joints and ligaments of knee; Injury of nerves at lower leg level; Injury of blood vessels at lower leg level; Injury of muscle and tendon at lower leg level; Crushing injury of lower leg; Traumatic amputation of lower leg; Other and unspecified injuries of lower leg; Superficial injury of ankle and foot; Open wound of ankle and foot; Fracture of foot except ankle; Dislocation, sprain, and strain of joints and ligaments at ankle and foot level; Injury of nerves at ankle and foot level; Injury of blood vessels at ankle and foot level; Injury of muscle and tendon at ankle and foot level; Crushing injury of ankle and foot; Traumatic amputation of ankle and foot; Other and unspecified injuries of ankle and foot; Superficial injuries involving multiple body regions; Open wounds involving multiple body regions; Fractures involving multiple body regions; Dislocations, sprains, and strains involving multiple body regions; Crushing injuries involving multiple body regions; Traumatic amputations involving multiple body regions; Other injuries involving multiple body regions, not elsewhere classified; Unspecified multiple injuries; Fracture of spine, level unspecified; Other injuries of spine and trunk, level unspecified; Fracture of upper limb, level unspecified; Other injuries of upper limb, level unspecified; Fracture of lower limb, level unspecified; Other injuries of lower limb, level unspecified; Injury of unspecified body region; Foreign body on external eye; Foreign body in ear; Foreign body in respiratory tract; Foreign body in alimentary tract; Foreign body in genitourinary tract; Burn and corrosion of head and neck; Burn and corrosion of trunk; Burn and corrosion of shoulder and upper limb except wrist and hand; Burn and corrosion of wrist and hand; Burn and corrosion of hip and lower limb except ankle and foot; Burn and corrosion of ankle and foot; Burn and corrosion confined to eye and adnexa; Burn and corrosion of respiratory tract; Superficial frostbite; Frostbite with tissue necrosis; Frostbite involving multiple body regions and unspecified frostbite; Other effects of reduced temperature; Effects of air pressure and water pressure; Certain early complications of trauma

Maternity and reproductive health: Obstetrical tetanus; Male infertility; Habitual aborter; Female infertility; Complications associated with artificial fertilization; Ectopic pregnancy; Hydatidiform mole; Other abnormal products of conception; Spontaneous abortion; Medical abortion ; Other abortion ; Unspecified abortion ; Pre-existing hypertension complicating pregnancy, childbirth and the puerperium; Preexisting hypertensive disorder with superimposed proteinuria; Gestational [pregnancy-induced] edema and proteinuria without hypertension; Gestational [pregnancy-induced] hypertension without significant proteinuria; Gestational [pregnancy-induced] hypertension with significant proteinuria; Eclampsia; Unspecified maternal hypertension; Hemorrhage in early pregnancy; Excessive vomiting in pregnancy; Venous complications in pregnancy; Infections of genitourinary tract in pregnancy; Diabetes mellitus in pregnancy; Malnutrition in pregnancy; Maternal care for other conditions predominantly related to pregnancy; Abnormal findings on antenatal screening of mother; Multiple gestation; Complications specific to multiple gestation; Maternal care for known or suspected malpresentation of fetus; Maternal care for known or suspected disproportion; Maternal care for known or suspected abnormality of pelvic organs; Maternal care for known or suspected fetal abnormality and damage; Maternal care for other known or suspected fetal problems; Polyhydramnios; Other disorders of amniotic fluid and membranes; Premature rupture of membranes; Placental disorders; Placenta previa; Premature separation of placenta [abruptio placentae]; Antepartum hemorrhage, not elsewhere classified; False labor; Prolonged pregnancy; Preterm delivery; Failed induction of labor; Abnormality of forces of labor; Long labor; Obstructed labor due to malposition and malpresentation of fetus; Obstructed labor due to maternal pelvic abnormality; Other obstructed labor; Labor and delivery complicated by intrapartum hemorrhage, not elsewhere classified; Labor and delivery complicated by fetal stress [distress]; Labor and delivery complicated by umbilical cord complications; Perineal laceration during delivery; Other obstetric trauma; Postpartum hemorrhage; Retained placenta and membranes, without hemorrhage; Other complications of labor and delivery, not

elsewhere classified; Infections of breast associated with childbirth; Other disorders of breast and lactation associated with childbirth; Maternal infectious and parasitic diseases classifiable elsewhere but complicating pregnancy, childbirth, and the puerperium; Other maternal diseases classifiable elsewhere but complicating pregnancy, childbirth, and the puerperium

Conditions of neonates: Tetanus neonatorum; Newborn affected by maternal conditions that may be unrelated to present pregnancy; Newborn affected by maternal complications of pregnancy; Newborn affected by complications of placenta, cord, and membranes; Newborn affected by other complications of labor and delivery; Newborn affected by noxious influences transmitted via placenta or breast milk; Slow fetal growth and fetal malnutrition; Disorders related to short gestation and low birthweight, not elsewhere classified; Disorders related to long gestation and high birthweight; Intracranial laceration and hemorrhage due to birth injury; Other birth injuries to central nervous system; Birth injury to scalp; Birth injury to skeleton; Birth injury to peripheral nervous system; Other birth injuries; Intrauterine hypoxia; Birth asphyxia; Respiratory distress of newborn; Congenital pneumonia; Neonatal aspiration syndromes; Interstitial emphysema and related conditions originating in the perinatal period; Pulmonary hemorrhage originating in the perinatal period; Chronic respiratory disease originating in the perinatal period; Other respiratory conditions originating in the perinatal period; Cardiovascular disorders originating in the perinatal period; Congenital viral diseases; Bacterial sepsis of newborn; Other congenital infectious and parasitic diseases; Omphalitis of newborn with or without mild hemorrhage; Other infections specific to the perinatal period; Fetal blood loss; Umbilical hemorrhage of newborn; Intracranial nontraumatic hemorrhage of fetus and newborn; Hemorrhagic disease of newborn; Other neonatal hemorrhages; Hemolytic disease of fetus and newborn; Hydrops fetalis due to hemolytic disease; Kernicterus; Neonatal jaundice due to other excessive hemolysis; Neonatal jaundice from other and unspecified causes; Disseminated intravascular coagulation of newborn; Other perinatal hematological disorders; Transitory disorders of carbohydrate metabolism specific to newborn; Transitory neonatal disorders of calcium and magnesium metabolism; Other transitory neonatal endocrine disorders; Other transitory neonatal electrolyte and metabolic disturbances; Other intestinal obstruction of newborn; Necrotizing enterocolitis of newborn; Other perinatal digestive system disorders; Hypothermia of newborn; Other disturbances of temperature regulation of newborn; Other conditions of integument specific to newborn; Convulsions of newborn; Other disturbances of cerebral status of newborn; Feeding problems of newborn; Reactions and intoxications due to drugs administered to newborn; Disorders of muscle tone of newborn; Other conditions originating in the perinatal period; Sudden infant death syndrome

Disease areas **without** measurable mortality effects from changes in expenditure

Mental Health Disorders: Vascular dementia; Unspecified dementia; Organic amnesic syndrome, not induced by alcohol and other psychoactive substances; Delirium, not induced by alcohol and other psychoactive substances; Other mental disorders due to brain damage and dysfunction and to physical disease; Personality and behavioral disorders due to brain disease, damage, and dysfunction; Unspecified organic or symptomatic mental disorder; Mental and behavioral disorders due to use of alcohol; Mental and behavioral disorders due to use of opioids ; Mental and behavioral disorders due to use of cannabinoids ; Mental and behavioral disorders due to use of sedatives or hypnotics ; Mental and behavioral disorders due to use of cocaine ; Mental and behavioral disorders due to use of other stimulants, including caffeine ; Mental and behavioral disorders due to use of hallucinogens ; Mental and behavioral disorders due to use of tobacco ; Mental and behavioral disorders due to use of volatile solvents ; Mental and behavioral disorders due to multiple drug use and use of other psychoactive substances ; Schizophrenia; Schizotypal disorder; Persistent delusional disorders; Acute and transient psychotic disorders; Induced delusional disorder; Schizoaffective disorders; Other nonorganic psychotic disorders; Unspecified nonorganic psychosis; Manic episode; Bipolar affective disorder; Depressive episode; Recurrent depressive disorder; Persistent mood [affective] disorders; Other mood [affective] disorders; Unspecified mood [affective] disorder; Reaction to severe stress, and adjustment disorders; Dissociative [conversion] disorders; Somatoform disorders; Other neurotic disorders; Eating disorders; Sexual dysfunction, not caused by organic disorder or disease; Mental and behavioral disorders associated with the puerperium, not elsewhere classified; Psychological and behavioral factors associated with disorders or diseases classified elsewhere; Abuse of non-dependence-producing substances; Unspecified behavioral syndromes associated with physiological disturbances and physical factors; Specific personality disorders; Mixed and other personality disorders; Enduring personality changes, not attributable to brain damage and disease; Habit and impulse disorders; Gender identity disorders; Disorders of sexual preference; Psychological and behavioral disorders associated with sexual development and orientation; Other disorders of adult personality and behavior; Unspecified disorder of adult personality and behavior; Conduct disorders; Mixed disorders of conduct and emotions; Emotional disorders with onset specific to childhood; Disorders of social functioning with onset specific to childhood and adolescence; Tic disorders; Other behavioral and emotional disorders with onset usually occurring in childhood and adolescence; Mental disorder, not otherwise specified; Alzheimer's disease; Phobic anxiety disorders; Other anxiety disorders; Obsessive-compulsive disorder; Nonorganic sleep disorders

Problems of the Musculoskeletal system: Tuberculosis of other organs; Pyogenic arthritis; Reactive arthropathies; Seropositive rheumatoid arthritis; Other rheumatoid arthritis; Juvenile arthritis; Gout; Other crystal arthropathies; Other specific arthropathies; Other arthritis; Polyarthrosis; Coxarthrosis [arthrosis of hip]; Gonarthrosis [arthrosis of knee]; Arthrosis of first carpometacarpal joint; Other arthrosis; Acquired deformities of fingers and toes; Other acquired deformities of limbs; Disorders of patella; Internal derangement of knee; Other specific joint derangements; Other joint disorders, not elsewhere classified; Polyarteritis nodosa and related conditions; Other necrotizing vasculopathies; Systemic lupus erythematosus; Dermatopolymyositis; Systemic sclerosis; Other systemic involvement of connective tissue; Kyphosis and lordosis; Scoliosis; Spinal osteochondrosis; Other deforming dorsopathies; Ankylosing spondylitis; Other inflammatory spondylopathies; Spondylosis; Other spondylopathies; Cervical disc disorders; Other intervertebral disc disorders; Other dorsopathies, not elsewhere classified; Dorsalgia; Myositis; Calcification and ossification of muscle; Other disorders of muscle; Synovitis and tenosynovitis; Spontaneous rupture of synovium and tendon; Other disorders of synovium and tendon; Soft tissue disorders related to use, overuse, and pressure; Other bursopathies; Fibroblastic disorders; Shoulder lesions; Enthesopathies of lower limb, excluding foot; Other enthesopathies; Other soft tissue disorders, not elsewhere classified; Osteoporosis with pathological fracture; Osteoporosis without pathological fracture; Adult osteomalacia; Disorders of continuity of bone; Other disorders of bone density and structure; Osteomyelitis; Osteonecrosis; Paget's disease of bone [osteitis deformans]; Other disorders of bone; Juvenile osteochondrosis of hip and pelvis; Other juvenile osteochondrosis; Other osteochondropathies; Other disorders of cartilage; Other acquired deformities of musculoskeletal system and connective tissue; Postprocedural musculoskeletal disorders, not elsewhere classified ; Biomechanical lesions, not elsewhere classified; Other congenital malformations of face and neck; Congenital deformities of hip; Congenital deformities of feet; Congenital musculoskeletal deformities of head, face, spine, and chest; Other congenital musculoskeletal

deformities; Polydactyly; Syndactyly; Reduction defects of upper limb; Reduction defects of lower limb; Reduction defects of unspecified limb; Other congenital malformations of limb(s); Other congenital malformations of skull and face bones; Congenital malformations of spine and bony thorax; Osteochondrodysplasia with defects of growth of tubular bones and spine; Other osteochondrodysplasias; Congenital malformations of musculoskeletal system, not elsewhere classified; Other specified congenital malformation syndromes affecting multiple systems

Others:

Disorders of Blood: Iron deficiency anemia; Vitamin B12 deficiency anemia; Folate deficiency anemia; Other nutritional anemias; Anemia due to enzyme disorders; Thalassemia; Sickle-cell disorders; Other hereditary hemolytic anemias; Acquired hemolytic anemia; Acquired pure red cell aplasia [erythroblastopenia]; Other aplastic anemias; Acute posthemorrhagic anemia; Other anemias; Disseminated intravascular coagulation [defibrination syndrome]; Hereditary factor VIII deficiency; Hereditary factor IX deficiency; Other coagulation defects; Purpura and other hemorrhagic conditions; Agranulocytosis; Functional disorders of polymorphonuclear neutrophils; Other disorders of white blood cells; Diseases of spleen; Methemoglobinemia; Other diseases of blood and blood-forming organs; Certain diseases involving lymphoreticular tissue and reticulohistiocytic system; Immunodeficiency with predominantly antibody defects; Combined immunodeficiencies; Immunodeficiency associated with other major defects; Common variable immunodeficiency; Other immunodeficiencies; Sarcoidosis; Other disorders involving the immune mechanism, not elsewhere classified; Other congenital malformations, not elsewhere classified; Elevated erythrocyte sedimentation rate and abnormality of plasma viscosity; Abnormality of red blood cells; Abnormality of white blood cells, not elsewhere classified; Other abnormalities of plasma proteins

Problems of Learning Disability: Mild mental retardation; Moderate mental retardation; Severe mental retardation; Profound mental retardation; Other mental retardation; Unspecified mental retardation; Specific developmental disorders of speech and language; Specific developmental disorders of scholastic skills; Specific developmental disorder of motor function; Mixed specific developmental disorders; Pervasive developmental disorders; Other disorders of psychological development; Unspecified disorder of psychological development; Hyperkinetic disorders; Down's syndrome; Edwards' syndrome and Patau's syndrome

Problems of Vision: Trachoma; Other diseases caused by chlamydiae; Viral conjunctivitis; Toxoplasmosis; Hordeolum and chalazion; Other inflammation of eyelid; Other disorders of eyelid; Disorders of lacrimal system; Disorders of orbit; Conjunctivitis; Other disorders of conjunctiva; Disorders of sclera; Keratitis; Corneal scars and opacities; Other disorders of cornea; Iridocyclitis; Other disorders of iris and ciliary body; Other disorders of lens; Chorioretinal inflammation; Other disorders of choroid; Retinal detachments and breaks; Retinal vascular occlusions; Other retinal disorders; Glaucoma; Disorders of vitreous body; Disorders of globe; Optic neuritis; Other disorders of optic [second] nerve and visual pathways; Paralytic strabismus; Other strabismus; Other disorders of binocular movement; Visual disturbances; Blindness and low vision; Nystagmus and other irregular eye movements; Other disorders of eye and adnexa; Postprocedural disorders of eye and adnexa, not elsewhere classified; Congenital malformations of eyelid, lacrimal apparatus, and orbit; Anophthalmos, microphthalmos, and macrophthalmos; Congenital lens malformations; Congenital malformations of anterior segment of eye; Congenital malformations of posterior segment of eye; Other congenital malformations of eye; Onchocerciasis; Senile cataract; Other cataract; Disorders of refraction and accommodation

Problems of Hearing: Otitis externa; Other disorders of external ear; Eustachian salpingitis and obstruction; Other disorders of Eustachian tube; Mastoiditis and related conditions; Cholesteatoma of middle ear; Perforation of tympanic membrane; Other disorders of tympanic membrane; Other disorders of middle ear and mastoid; Otosclerosis; Disorders of vestibular function; Other diseases of inner ear; Other disorders of ear, not elsewhere classified; Postprocedural disorders of ear and mastoid process, not elsewhere classified ; Nonsuppurative otitis media; Suppurative and unspecified otitis media; Congenital malformations of ear causing impairment of hearing; Other congenital malformations of ear; Conductive and sensorineural hearing loss; Other hearing loss

Dental problems: Disorders of tooth development and eruption; Embedded and impacted teeth; Dental caries; Other diseases of hard tissues of teeth; Diseases of pulp and periapical tissues; Gingivitis and periodontal diseases; Other disorders of gingiva and edentulous alveolar ridge; Dentofacial anomalies [including malocclusion]; Other disorders of the teeth and supporting structures; Cysts of oral region, not elsewhere classified; Other diseases of jaws

Problems of the Skin: Anthrax; Erysipeloid; Listeriosis; Erysipelas; Yaws; Herpesviral [herpes simplex] infections; Viral warts; Other viral infections characterized by skin and mucous membrane lesions, not elsewhere classified; Dermatophytosis; Other superficial mycoses; Chromomycosis and pheomycotic abscess; Pediculosis and phthiriasis; Scabies; Myiasis; Staphylococcal scalded skin syndrome; Impetigo; Cutaneous abscess, furuncle, and carbuncle; Cellulitis; Acute lymphadenitis; Pilonidal cyst; Other local infections of skin and subcutaneous tissue; Pemphigus; Other acantholytic disorders; Pemphigoid; Other bullous disorders; Atopic dermatitis; Seborrheic dermatitis; Diaper [napkin] dermatitis; Allergic contact dermatitis; Irritant contact dermatitis; Unspecified contact dermatitis; Exfoliative dermatitis; Dermatitis due to substances taken internally; Lichen simplex chronicus and prurigo; Pruritus; Other dermatitis; Psoriasis; Parapsoriasis; Pityriasis rosea; Lichen planus; Other papulosquamous disorders; Urticaria; Erythema multiforme; Erythema nodosum; Other erythematous conditions; Sunburn; Skin changes due to chronic exposure to nonionizing radiation; Radiodermatitis; Other disorders of skin and subcutaneous tissue related to radiation; Nail disorders; Alopecia areata; Other nonscarring hair loss; Cicatricial alopecia [scarring hair loss]; Hair color and hair shaft abnormalities; Hypertrichosis; Acne; Rosacea; Follicular cysts of skin and subcutaneous tissue; Other follicular disorders; Eccrine sweat disorders; Apocrine sweat disorders; Vitiligo; Other disorders of pigmentation; Seborrheic keratosis; Acanthosis nigricans; Corns and callousities; Other epidermal thickening; Transepidermal elimination disorders; Pyoderma gangrenosum; Decubitus ulcer; Atrophic disorders of the skin; Hypertrophic disorders of the skin; Granulomatous disorders of skin and subcutaneous tissue; Lupus erythematosus; Other localized connective tissue disorders; Vasculitis limited to the skin, not elsewhere classified; Ulcer of lower limb, not elsewhere classified; Other disorders of skin and subcutaneous tissue, not elsewhere classified; Inflammatory disorders of breast; Hypertrophy of breast; Other disorders of breast; Congenital ichthyosis; Epidermolysis bullosa; Other congenital malformations of skin; Congenital malformations of breast; Other congenital malformations of integument; Phakomatoses, not elsewhere classified; Rash and other nonspecific skin eruption; Localized swelling, mass, and lump of skin and subcutaneous tissue; Other skin changes; Abnormal findings on diagnostic imaging of breast; Burn and corrosion of other internal organs; Burn and corrosion, body region unspecified; Burns classified according to extent of body surface involved; Corrosions classified according to extent of body surface involved

Adverse events and poisoning: Postprocedural disorders of digestive system, not elsewhere classified ; Other acute skin changes due to ultraviolet radiation; Androgenic alopecia; Failed attempted abortion; Complications of anesthesia during pregnancy; Complications of anesthesia during labor and delivery; Puerperal sepsis; Other puerperal infections; Venous complications in the puerperium; Obstetric embolism; Complications of anesthesia during the puerperium; Complications of the puerperium, not elsewhere classified; Obstetric death of unspecified cause; Death from any obstetric cause occurring more than 42 days but less than 1 year after delivery; Death from sequelae of direct obstetric causes; Congenital malformation syndromes due to known exogenous causes, not elsewhere classified; Complications following abortion and ectopic and molar pregnancy; Other disorders of the genitorurinary system; Findings of drugs and other substances, not normally found in blood; Poisoning by systemic antibiotics; Poisoning by other systemic anti-infectives and antiparasitics; Poisoning by hormones and their synthetic substitutes and antagonists, not elsewhere classified; Poisoning by nonopioid analgesics, antipyretics, and antirheumatics; Poisoning by narcotics and psychodysleptics [hallucinogens]; Poisoning by anesthetics and therapeutic gases; Poisoning by antiepileptic, sedative-hypnotic, and antiparkinsonism drugs; Poisoning by psychotropic drugs, not elsewhere classified; Poisoning by drugs primarily affecting the autonomic nervous system; Poisoning by primarily systemic and hematological agents, not elsewhere classified; Poisoning by agents primarily affecting the cardiovascular system; Poisoning by agents primarily affecting the gastrointestinal system; Poisoning by agents primarily acting on smooth and skeletal muscles and the respiratory system; Poisoning by topical agents primarily affecting skin and mucous membrane and by ophthalmological, otorhinolaryngological, and dental drugs; Poisoning by diuretics and other and unspecified drugs, medicaments, and biological substances; Toxic effect of alcohol; Toxic effect of organic solvents; Toxic effect of halogen derivatives of aliphatic and aromatic hydrocarbons; Toxic effect of corrosive substances; Toxic effect of soaps and detergents; Toxic effect of metals; Toxic effect of other inorganic substances; Toxic effect of carbon monoxide; Toxic effect of other gases, fumes, and vapors; Toxic effect of pesticides; Toxic effect of noxious substances eaten as seafood; Toxic effect of other noxious substances eaten as food; Toxic effect of contact with venomous animals; Toxic effect of aflatoxin and other mycotoxin food contaminants; Toxic effect of other and unspecified substances;

Unspecified effects of radiation; Effects of heat and light; Hypothermia; Asphyxiation; Effects of other deprivation; Maltreatment syndrome; Effects of other external causes; Adverse effects, not elsewhere classified; Complications following infusion, transfusion, and therapeutic injection; Complications of procedures, not elsewhere classified; Complications of cardiac and vascular prosthetic devices, implants, and grafts; Complications of genitourinary prosthetic devices, implants, and grafts; Complications of internal orthopedic prosthetic devices, implants, and grafts; Complications of other internal prosthetic devices, implants, and grafts; Failure and rejection of transplanted organs and tissues; Complications peculiar to reattachment and amputation; Other complications of surgical and medical care, not elsewhere classified

Other: Other trisomies and partial trisomies of autosomes, not elsewhere classified; Monosomies and deletions from the autosomes, not elsewhere classified; Balanced rearrangements and structural markers, not elsewhere classified; Turner's syndrome; Other sex chromosome abnormalities, female phenotype, not elsewhere classified; Other sex chromosome abnormalities, male phenotype, not elsewhere classified; Other chromosome abnormalities, not elsewhere classified; Malaise and fatigue; Senility; Hemorrhage, not elsewhere classified; Enlarged lymph nodes; Edema, not elsewhere classified; Lack of expected normal physiological development; Other abnormal immunological findings in serum; Other abnormal findings in urine; Abnormal findings in specimens from other organs, systems, and tissues; Other sudden death, cause unknown; Unattended death
