# Supplementary material for: Health Opportunity Costs: Assessing the Implications of Uncertainty Using Elicitation Methods with Experts
Source: Med Decis Making. 2020 May 22;40(4):448–59. doi: 10.1177/0272989X20916450 (PMC7509606; doi:10.1177/0272989X20916450)
Supplement: Manuscript_expert_elicitation_HOC_MDM_4_Appendix2_online_supp – Supplemental material for Health Opportunity Costs: Assessing the Implications of Uncertainty Using Elicitation Methods with Experts [file Manuscript_expert_elicitation_HOC_MDM_4_Appendix2_online_supp.pdf]

CHE
Centre For Health Economics

Health opportunity costs in the NHS:
assessing the implications of uncertainty using elicitation methods with experts

Work funded by the Department of Health's Policy Research Unit in Economic Evaluation of Health and Care Interventions

Marta Soares, Karl Claxton, Mark Sculpher

Centre for Health Economics, York

Why do health opportunity costs matter?

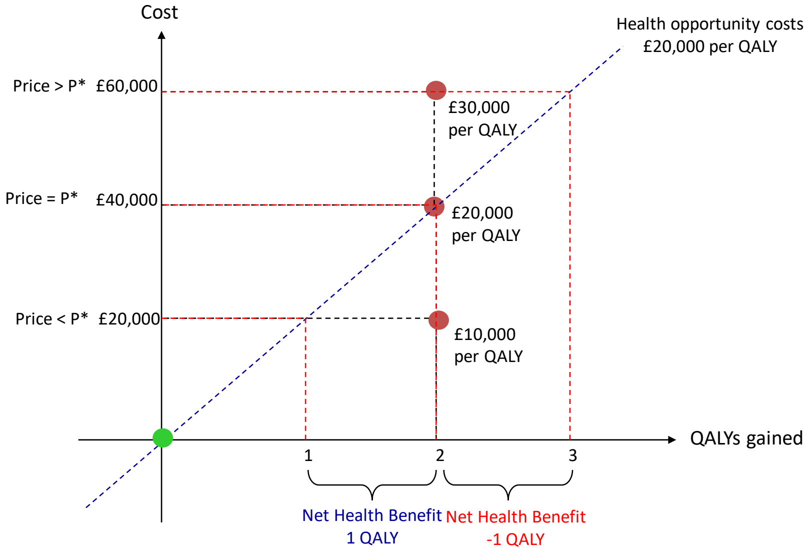

Need to compare benefits to opportunity costs

- What are the additional health benefits and additional costs of a proposed investment?
- What are the health effects of other things we could choose to do *or others are likely to do if the resources where made available for other uses?*
- What are the health effects of those things we will need to give up *or others are likely to give up if we commit these resources?*
- Expected health effects of changes in health expenditure
  - *Supply side – what we currently get from changes in NHS resources*

How can we estimate it?

- Estimate the relationship between changes in expenditure and outcomes
  - 23 Programme Budget Categories (PBCs)
    - Disease areas (groups of ICD codes)
    - All NHS expenditure allocated to each PBC
  - 152 Primary Care Trusts (PCTs)
    - PBC expenditure by PCT
    - PBC outcomes by PCT (mortality by ICD code)

- Claxton, K., Martin, S., Soares, M., et al.. Methods for the estimation of the NICE cost effectiveness threshold. **Health Technology Assessment**, 2015; 19(14) (see web page for more materials about this research https://www.york.ac.uk/che/research/teehta/thresholds/)
- Martin S, Rice N, Smith PC. Comparing costs and outcomes across programmes of health care. **Health Economics**. 2012 Mar;21(3):316-337.
- Martin S, Rice N, and Smith PC. Does health care spending improve health outcomes? Evidence from English programme budgeting data. **Journal of Health Economics** 2008; 27:826–42.

How can we estimate effects of expenditure on mortality?

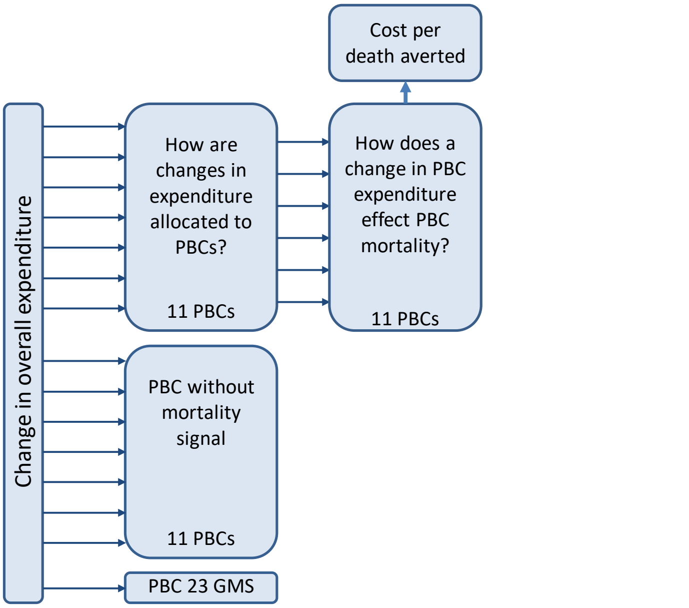

How can we estimate effects on life years

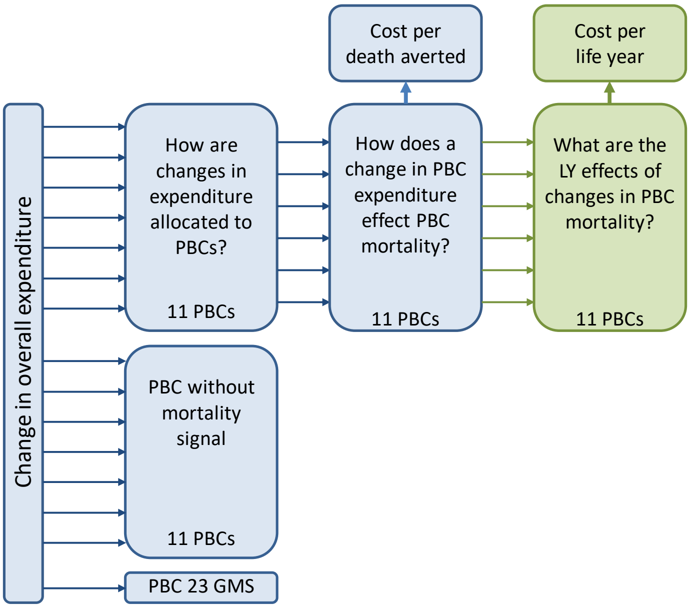

How can we adjust life years for quality?

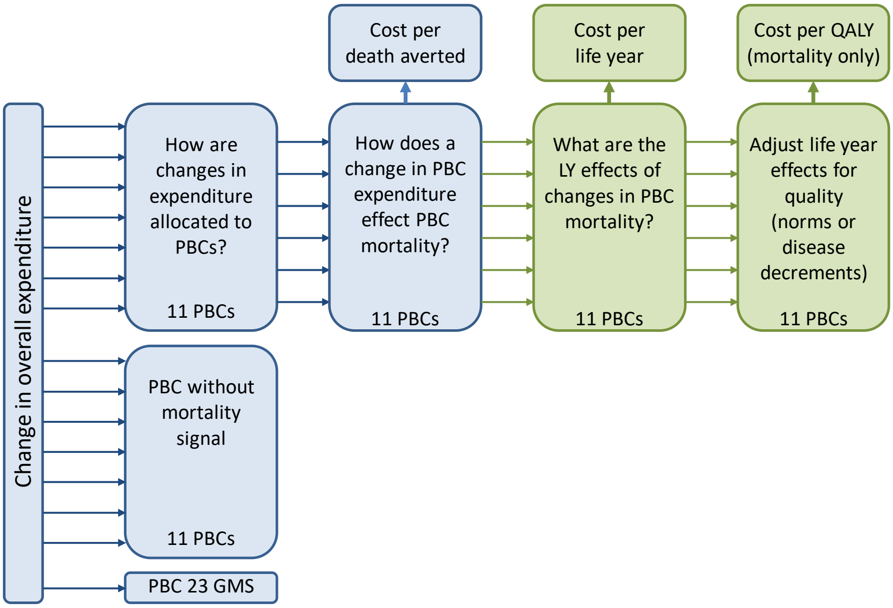

How can we account for possible effects on quality of life?

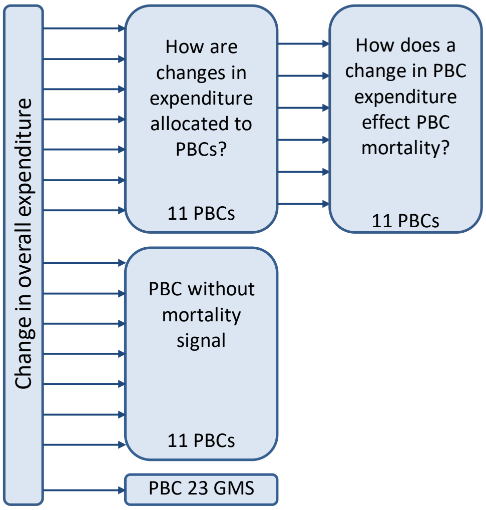

How can we account for possible effects on quality of life?

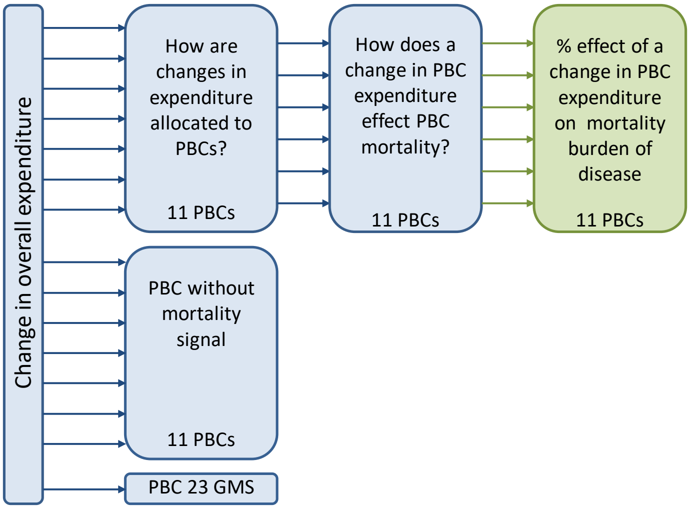

Surrogacy. First 11 PBCs.

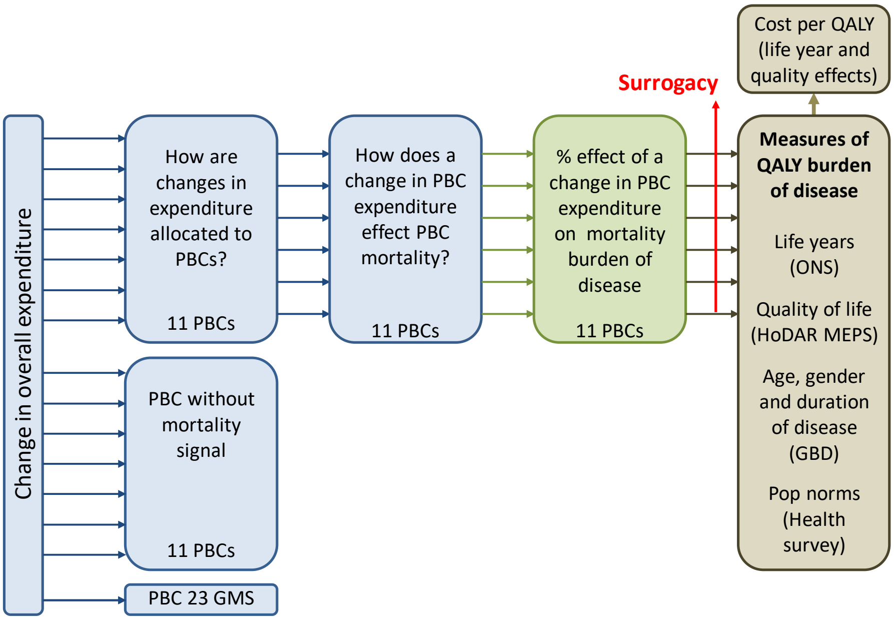

Extrapolation. Other 11 PBCs.

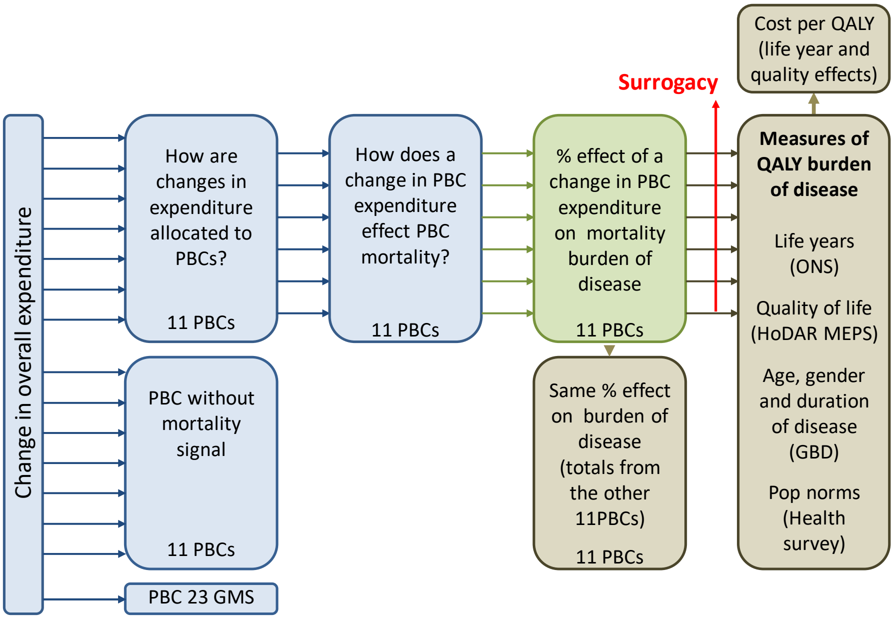

Extrapolation. Other 11 PBCs.

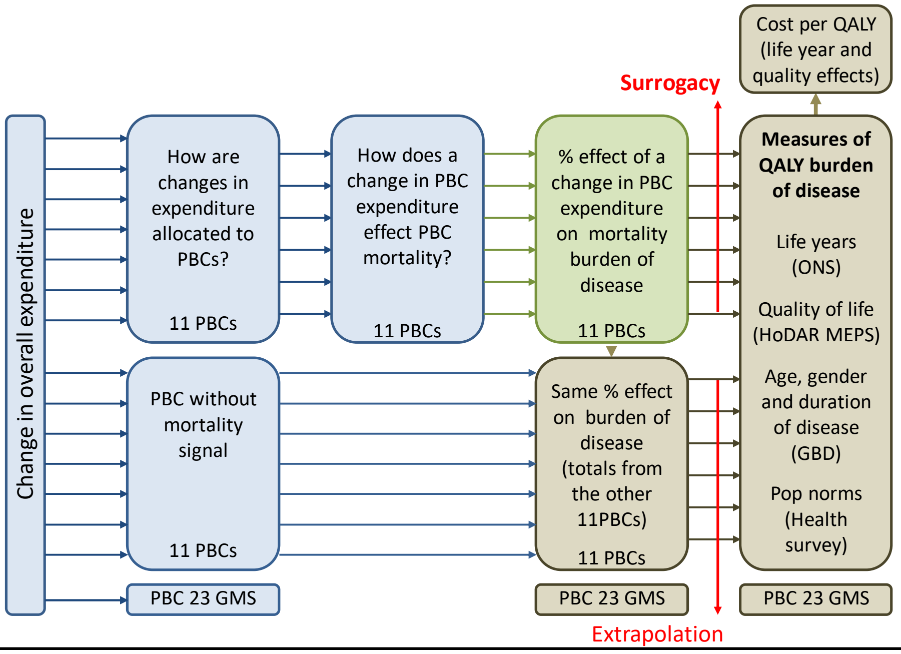

Estimates of health opportunity costs

	Cost per death averted	Cost per life year	Cost per QALY (mortality effects)	Cost per QALY
Qol associated with LYs	*-*	*1*	*Norms*	*Based on burden*
Qol during disease	*-*	*0*	*0*	*Based on burden*
YLL per death averted	*-*	*4.5 YLL*	*4.5 YLL*	*4.5 YLL*
QALYs per death averted	*-*	*4.5 YLL*	*3.8 QALY*	*12.7 QALY*
11 PBCs (with mortality)	£105,872	£23,360	£28,045	£8,308
All 23 PBCs				**£12,936**

The expected health opportunity costs of £10m?

	Change in spend	Additional deaths	LY lost	Total QALY lost	Due to premature death	Quality of life effects
**Totals**	**10 (£m)**	**51**	**233**	**773**	**150**	**623**
Cancer	0.45	3.74	**37.5**	26.3	24.4	1.9
Circulatory	0.76	22.78	**116.0**	107.8	73.7	34.1
Respiratory	0.46	13.37	**16.1**	229.4	10.1	**219.3**
Gastro-intestinal	0.32	2.62	**24.7**	43.9	16.2	27.7
Infectious diseases	0.33	0.72	5.3	15.7	3.6	12.1
Endocrine	0.19	0.67	5.0	60.6	3.2	57.3
Neurological	0.60	1.21	6.5	109.1	4.3	**104.8**
Genito-urinary	0.46	2.25	3.3	10.6	2.1	8.5
Trauma & injuries*	0.77	0.00	0.0	0.0	0.0	0.0
Maternity & neonates*	0.68	0.01	0.4	0.2	0.2	0.1
Disorders of Blood	0.21	0.36	1.7	21.8	1.1	20.7
Mental Health	1.79	2.83	12.8	95.3	8.3	**87.0**
Learning Disability	0.10	0.04	0.2	0.7	0.1	0.6
Problems of Vision	0.19	0.05	0.2	4.2	0.2	4.1
Problems of Hearing	0.09	0.03	0.1	14.0	0.1	13.9
Dental problems	0.29	0.00	0.0	6.8	0.0	6.8
Skin	0.20	0.24	1.1	1.9	0.7	1.2
Musculo skeletal	0.36	0.39	1.8	23.2	1.2	22.1
Poisoning and AE	0.09	0.04	0.2	0.8	0.1	0.7
Healthy Individuals	0.35	0.03	0.2	0.7	0.1	0.6
Social Care Needs	0.30	0.00	0.0	0.0	0.0	0.0
Other (GMS)	1.01	0.00	0.0	0.0	0.0	0.0

Critical assumptions to focus on for the elicitation:

Duration of effects:

changes in expenditure may have an effect beyond the year of expenditure

Effects restricted to the year of expenditure change in Claxton 2015

Surrogacy

how the effects of changes in expenditure on mortality relate to effects on a broader measure of health that incorporates both duration and quality of life impacts

Assumed to be proportionate in Claxton 2015

15

Critical assumptions to focus on for the elicitation:

Extrapolation

how changes in expenditure affect health in disease areas for which previous work could not estimate a mortality effect

Assumed similar proportionate effect on burden in Claxton 2015

Life years associated with reductions in mortality

the additional life-years associated with any reduction in the rate of mortality

No assumption needed for the cost per QALY reported in Claxton 2015

Costs per life year reported in Claxton et al 2015 assumed that avoiding mortality (with a minimum survival benefits of 2 years on average) returns patients to the mortality risk of the general population matched for age and gender

16

Elicitation. Aims

- Elicitation: your beliefs expressed in a numerical form.
- may be things that you already have opinions on but others will require some deep thinking.
- It does **not** mean that you are expected to know the answer to all questions.

  If you are unsure about (or don't know the answer to) a question you should still answer it. Just express how uncertain you are about it in your response
- You will be asked to give your opinion **individually** (not in groups), so please try and interact the least possible with colleagues.
- The information you provide, including any personal details, will be kept anonymous and confidential, stored securely and only accessed by those carrying out the study.

17

Elicitation. Experts

We elicited from individuals with substantial expertise in particular clinical areas – clinical experts.

Disease areas: those where the uncertainties have more influence on overall estimates of health opportunity costs.

**Clinical areas from which clinical experts are drawn**
Circulatory
Respiratory
Gastrointestinal
Neurological
Mental health
Endocrinology
Musculoskeletal
Primary care

18

Elicitation. Experts

In this workshop, policy experts are asked to elicit the same information. You will be able to consult the answers given by clinical experts (anonymously, by clinical area of expertise)

- policy experts: drawn from organisations that develop or implement policy, or that have a major interest in policy in this area.

**Organisations from which policy experts are drawn**
Department of Health
NHSE
PHE
NICE
JCVI
NHSCC
ABPI
Patients' organisations

19

Expert elicitation. Heuristics and biases

- Structured elicitation: aimed at minimising the use of heuristics and at avoiding biases.
- Heuristics:
  - Mental 'rules of thumb' used to avoid complex tasks by making simpler judgmental operations Kahneman and Tversky
  - Heuristics often cause bias
  - Be aware of heuristics, and try to avoid them!

20

Expert elicitation. Heuristics and biases

- Anchoring heuristic
  - When experts make estimates by starting from an initial value (or scenario) and adjusting it to get the final answer, the adjustment is usually insufficient
- Availability heuristic:
  - Judging the probability of an event based on what is easier to recall
- Representativeness heuristic
  - Where experts identify a familiar object or event and use it to represent the current issue.
- Be aware of overconfidence

21

Eliciting uncertainty. What do we mean?

- What is the average age of people that go to the theatre?

I believe the average age is 55 years but it could be as low as 45 or as high as 65. This *is* uncertainty!

It should not consider differences between individuals e.g. one individual may actually be 17, and another 90 years old. This *is not* uncertainty, but variability!

We may also be tempted to think of one group e.g. audience of pantomime, which may have a very different average age. This *is not* uncertainty, but reflects heterogeneity

22

How are you going to express your answers

For every 1,000 women aged 15-44 resident in England and Wales how many had an abortion in 2015?

My best guess for the value of this quantity is:	I am very certain (90% certain) that the true value for this quantity is … … higher than:	… lower than:
16	2	100

How are you going to express your answers

For every 1,000 women aged 15-44 resident in England and Wales how many had an abortion in 2015?

My best guess for the value of this quantity is:	I am very certain (90% certain) that the true value for this quantity is … … higher than:	… lower than:
16	10	25

In 2015, the age-standardised abortion rate was 16.0 per 1,000 resident women aged 15-44, https://www.gov.uk/government/uploads/system/uploads/attachment_data/file/570040/Updated_Abortion_Statistics_2015.pdf

How are you going to express your answers

Try it yourself!

In 2015, for every 1,000 women aged 15-44 resident in England and Wales, how many had an abortion and were aged under 16 years old?

My best guess for the value of this quantity is:	I am very certain (90% certain) that the true value for this quantity is … … higher than:	… lower than:
_______	_______	_______

How are you going to express your answers

Try it yourself!

The previous question asked about the rate of abortions in under 16s (number per 1000 women) for 2015. How much higher (or lower) was this rate when compared to 2005?

My best guess for the value of this quantity is:	I am very certain (90% certain) that the true value for this quantity is … … higher than:	… lower than:
_______	_______	_______

Quantities to elicit:

Four sections:

A. duration of effects

B. surrogacy

C. extrapolation

D. life years associated with reductions in mortality

Context for the quantities we aim to elicit

The questionnaire focusses on the effects to population health of changes to NHS expenditure in a particular year (all else remaining unchanged).

These may be increases or decreases in expenditure but, for clarity, throughout the questionnaire we will refer to increases in expenditure.

We would like you to think of changes in expenditure that are significant, but still represent a small proportion of NHS' expenditure.

Context for the quantities we aim to elicit

We will ask you to consider disease areas such as ‘problems of the respiratory system’ or ‘problems of the musculoskeletal system’. Each disease area is broad and in Appendix 1 you will find the ICD codes that define them.

The answers we need should consider the ICDs within a disease area where a change in expenditure is more likely to fall.

Training on A.

A. Duration of mortality effects

If, in a particular year, NHS expenditure is increased for a specific disease area we expect a lower disease-related mortality rates in that same year.

Increasing expenditure in a particular year may, however, also affect the disease-specific mortality rates in subsequent years (of those same individuals or other individuals being cared for in the future).

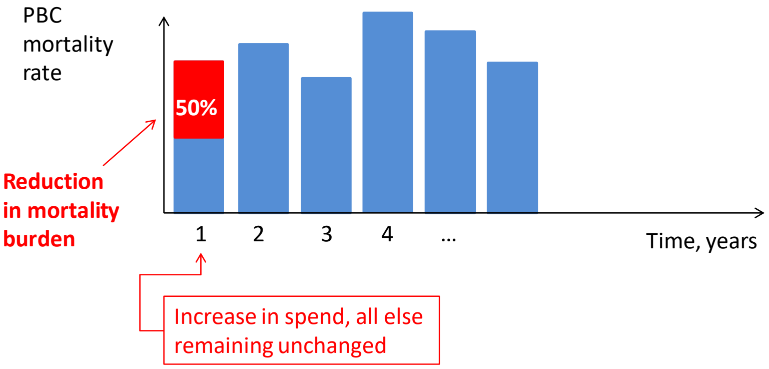

31

A. Duration of mortality effects. Elicitation

A1. On average, for how many more years (beyond the year of increased expenditure) would you expect the disease-specific mortality rate to be reduced?

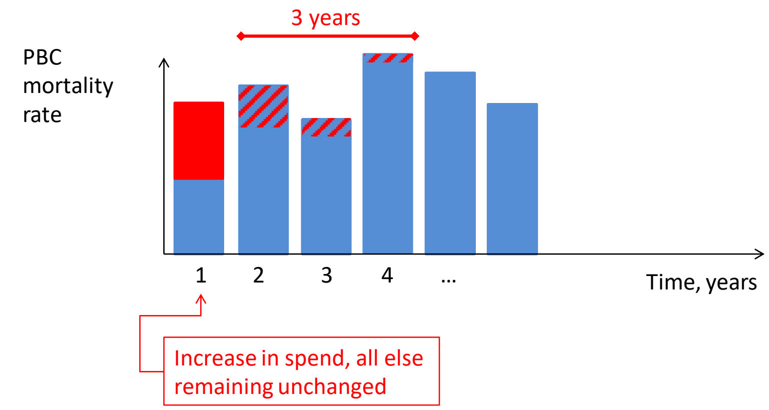

32

A. Duration of mortality effects. Elicitation

A2. From an increase in expenditure in a particular year, how do reductions in mortality rates in subsequent years compare to the reduction observed in the first year.

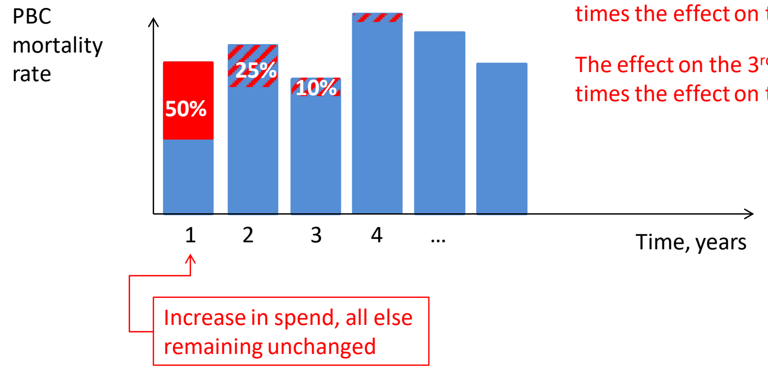

The effect on the 2nd year is 0.5 times the effect on the 1st year

The effect on the 3rd year is 0.2 times the effect on the 1st year

33

A. Duration of mortality effects. Elicitation

A3. Are you confident the answers you gave to questions A1 and A2 reflect your views and uncertainties?

YES NOT SURE NO (circle your response).

If you responded NOT SURE or NO, please give us some more detail as to why:

____

____

____

34

A. Duration of mortality effects. Clinical experts responses

	Experts from the particular clinical area*					Distribution across all experts		
	#1	#2	#3	#4	#5	Mode	Lower bound	Upper bound
Circulatory	3 (2,6)	5 (3,10)	10 (5,25)	3 (2,10)	15 (5,20)			
Endocrinology	2 (0,3)	1 (0,4)	5 (3,12)					
Others w mortality								

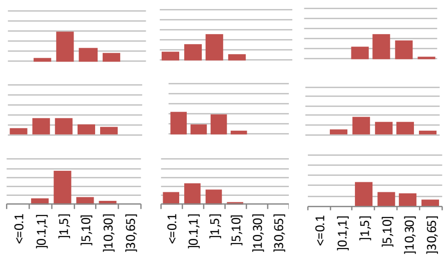

Please proceed to elicit quantity A.

Training on B.

B. Surrogacy. Health burden profile of disease

Broader *health burden* of disease that considers its impact on both the rate of mortality (and any life years lost as a consequence) and on the level of health-related quality of life of individuals.

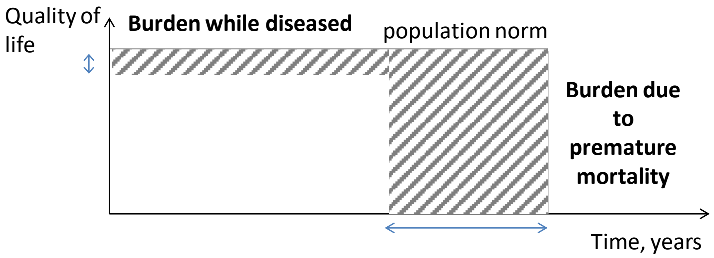

B. Surrogacy. Health burden profile of disease

Broader *health burden* of disease that considers its impact on both the rate of mortality (and any life years lost as a consequence) and on the level of health-related quality of life of individuals.

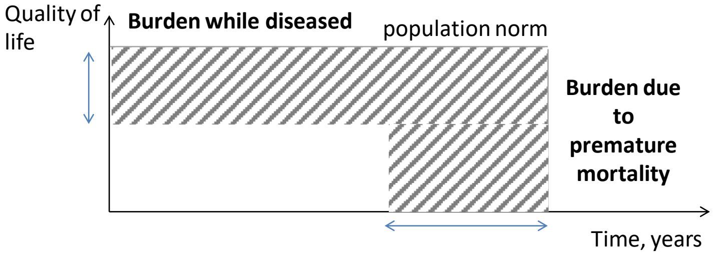

39

B. Surrogacy. Health burden profile of disease, 1st year

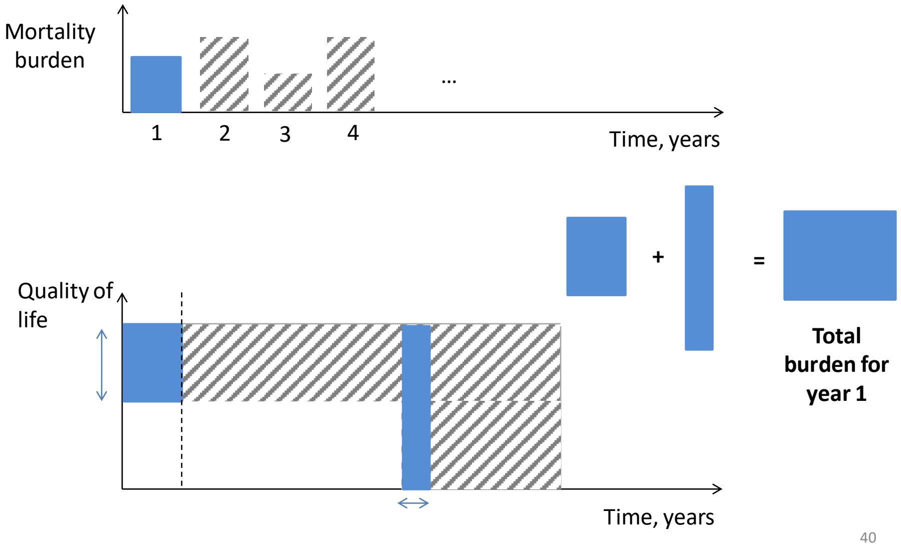

40

B. Surrogacy. Health burden profile of disease, 2nd year

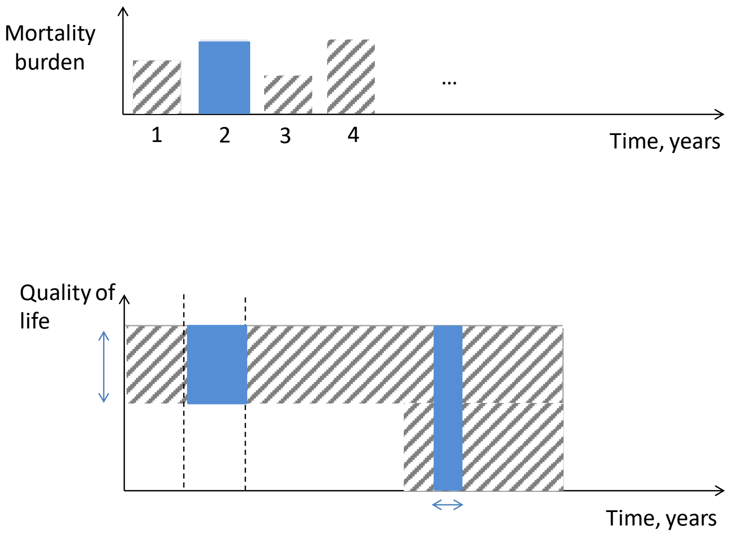

B. Surrogacy. Health burden profile of disease, 3rd year

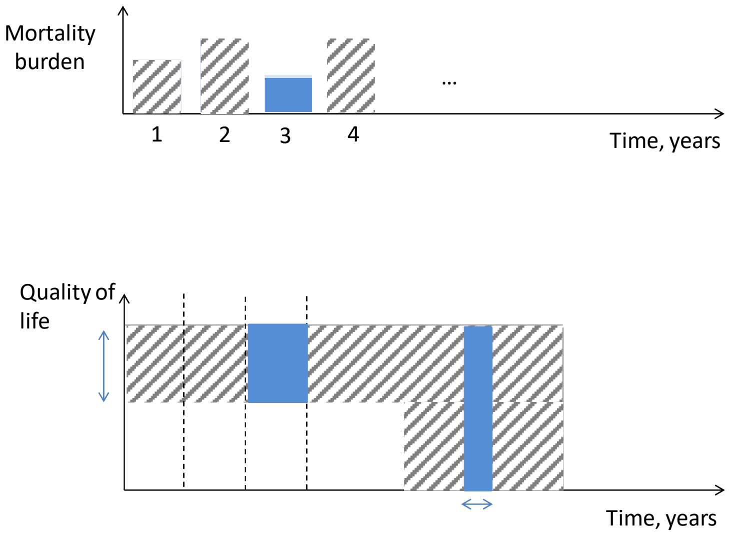

B. Surrogacy. Effects of expenditure, 1st year

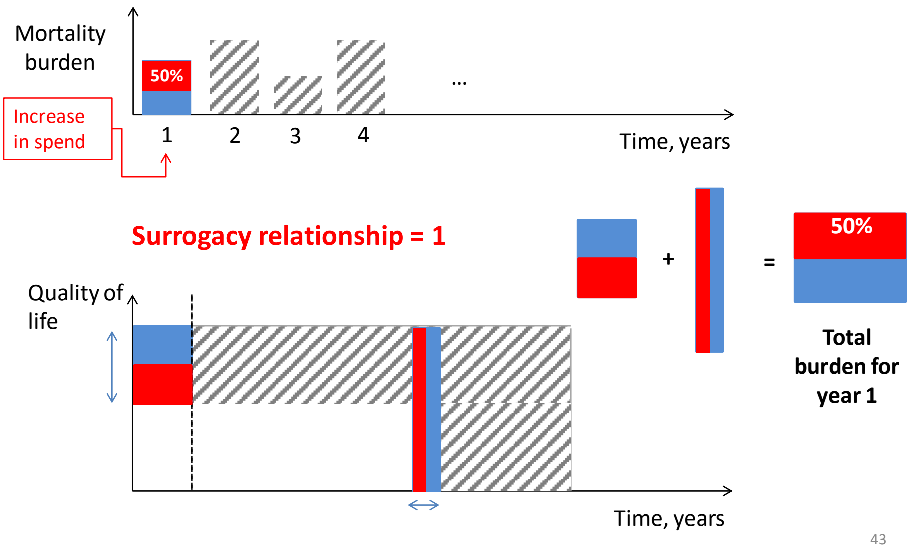

43

B. Surrogacy. Effects of expenditure, 2nd year

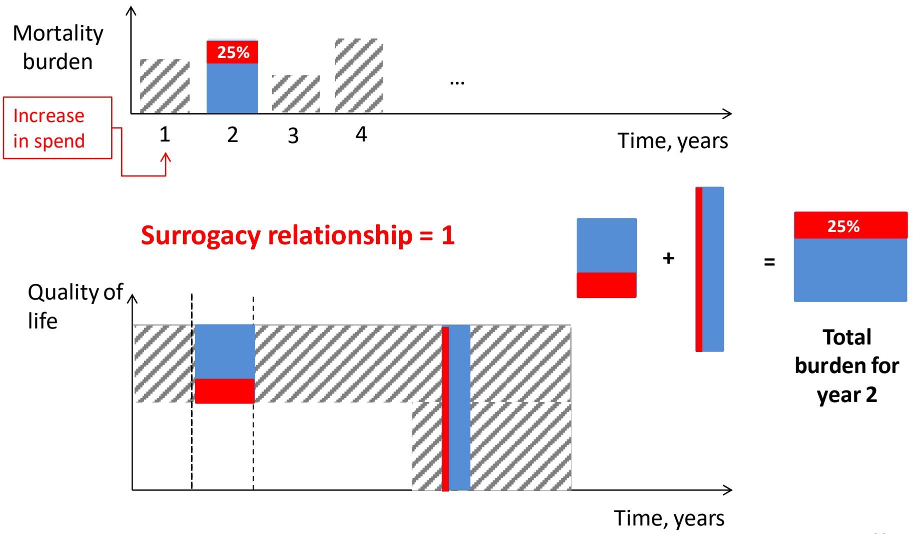

44

B. Surrogacy. Effects of expenditure, 3rd year

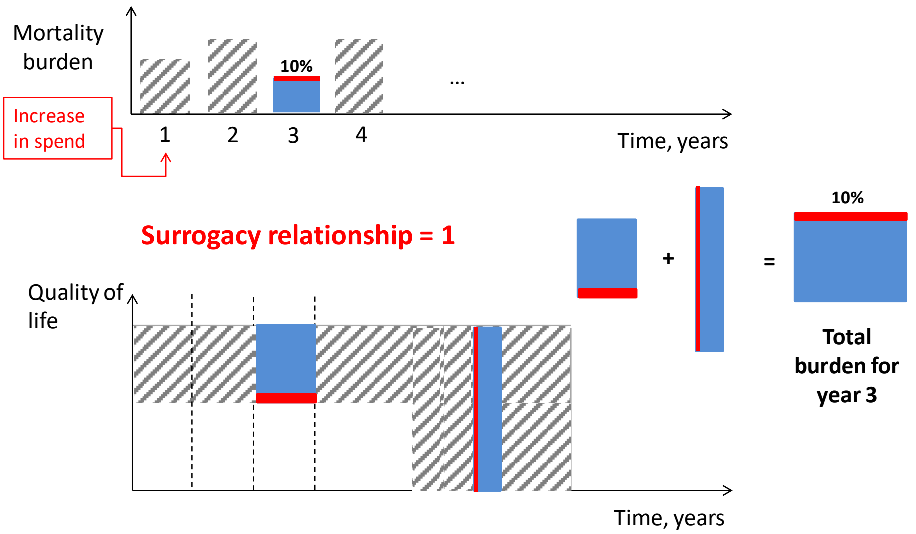

45

B. Surrogacy. Clinical experts responses

	Experts from the particular clinical area*					Distribution across all experts		
	#1	#2	#3	#4	#5	Mode	Lower bound	Upper bound
Respiratory (1 yr)	0.7 (0.5,1.3)	1.5 (0.8,2)						
Respiratory (2 yr)	0.8 (0.5,1.5)	1 (0.5,1.5)						
Respiratory (3 yr)	0.9 (0.6,2)	0.5 (0.3,1.5)						
Respiratory (4 yr)	1 (0.6,2.5)	0.2 (0.1,1.5)						

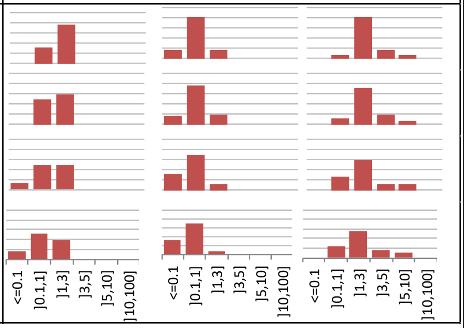

46

Please proceed to elicit quantity **B.**

Training on **C.**

C. Extrapolation. Effects of expenditure, 1st year

Reductions in health burden in the 1st year from increased expenditure can now be determined across all disease areas with measurable mortality effects – for illustration, assume this is **50%.**

How do reductions in health burden from an increase in NHS expenditure in 'mental health', for example, compare to the above?

Extrapolation relationship = 1

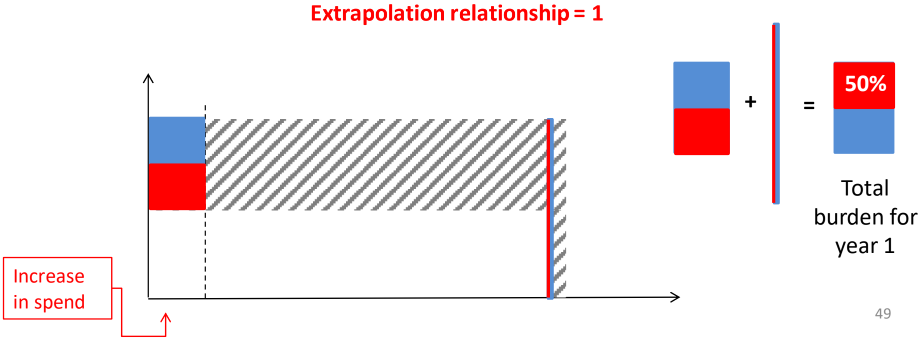

49

C. Extrapolation. Effects of expenditure, 2nd year

For illustration, assume the effect of spend across all disease areas with a measurable effect of spend on mortality on the 2nd year is **25%**

How do reductions in health burden from an increase in NHS expenditure in 'mental health', for example, compare to the above?

Extrapolation relationship = 1

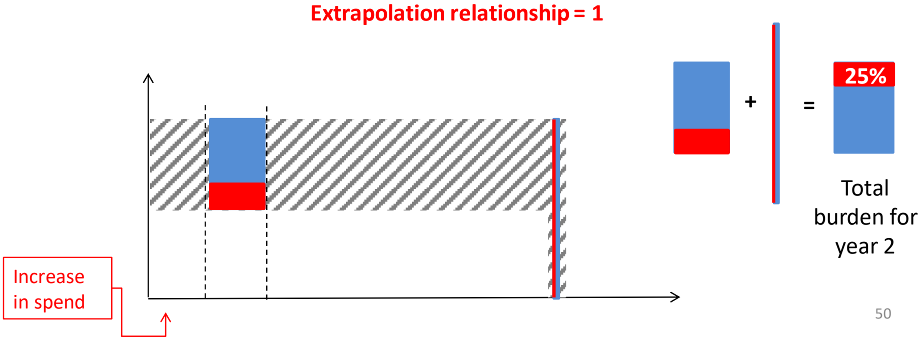

50

C. Extrapolation. Effects of expenditure, 3rd year

For illustration, assume the effect of spend across all disease areas with a measurable effect of spend on mortality on the 3rd year is **10%**

How do reductions in health burden from an increase in NHS expenditure in 'mental health', for example, compare to the above?

Extrapolation relationship = 1

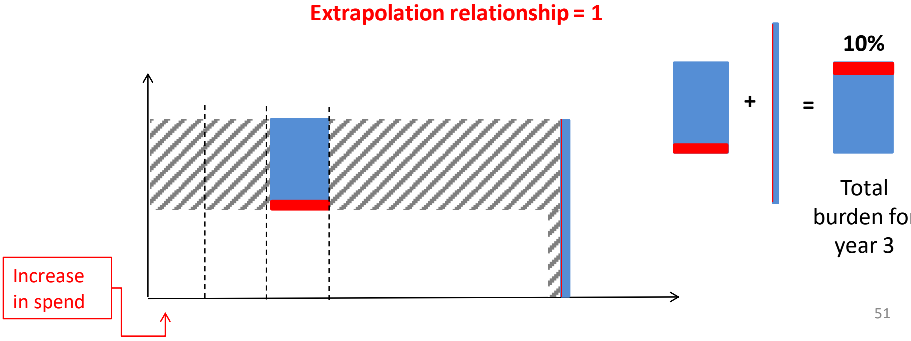

C. Extrapolation. Clinical experts responses

	Experts from the particular clinical area*					Distribution across all experts		
	#1	#2	#3	#4	#5	Mode	Lower bound	Upper bound
Musculoskeletal (1 yr)	4 (2,6)							
Musculoskeletal (2 yr)	3 (1.5,4.5)							
Musculoskeletal (3 yr)	2.5 (1.5,3.5)							
Musculoskeletal (4 yr)	2 (1.25,2.75)							

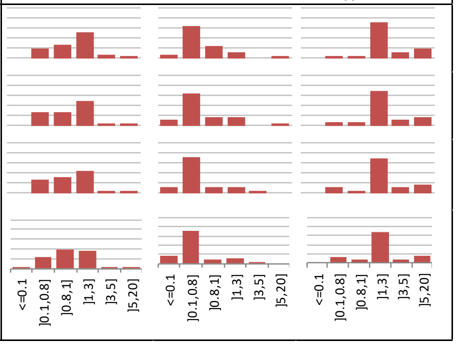

Please proceed to elicit quantity C.

Training on D.

D. Life years associated with reductions in mortality.

What are the implications to LYs gained at life expectancy?

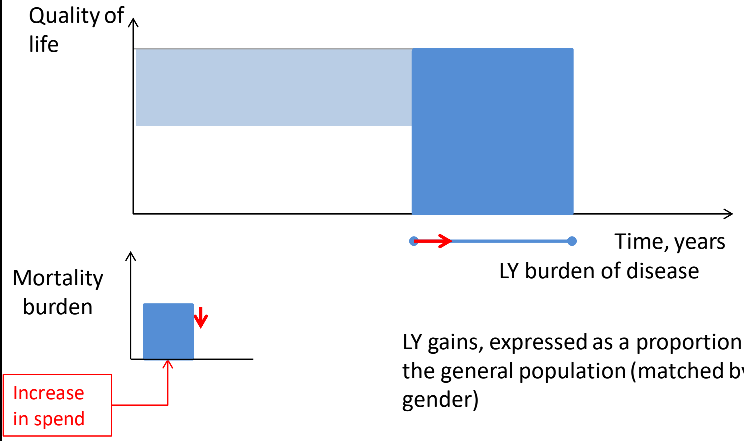

LY gains, expressed as a proportion of the LE of the general population (matched by age and gender)

55

D. Life years associated with reductions in mortality.

There is more information we can draw from the existing data…

Let's have another look at the mortality data analysed in Claxton et al 2015:

At PCT level, PBC mortality for 3 consecutive years was averaged

PBC spend in a particular year was then regressed against mortality

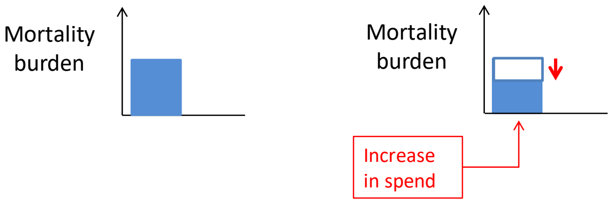

56

D. Life years associated with reductions in mortality.

There is more information we can draw from the existing data….

Let’s have another look at the mortality data analysed in Claxton et al 2015:

At PCT level, PBC mortality for 3 consecutive years was averaged

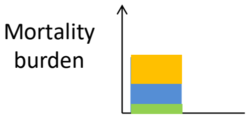

Some deaths occured in the 3rd year

Others in the 2nd year

Others in the 1st year

57

D. Life years associated with reductions in mortality.

There is more information we can draw from the existing data….

Let’s have another look at the mortality data analysed in Claxton et al 2015:

At PCT level, PBC mortality for 3 consecutive years was averaged

PBC spend in a particular year was then regressed against mortality

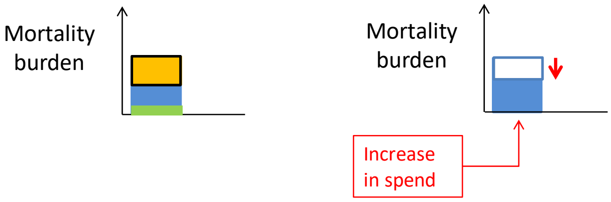

Deaths in the 3rd year could only be counted as averted if they didn’t occur within that same year – averted for at least 1 year

58

D. Life years associated with reductions in mortality.

There is more information we can draw from the existing data….

Let's have another look at the mortality data analysed in Claxton et al 2015:

At PCT level, PBC mortality for 3 consecutive years was averaged

PBC spend in a particular year was then regressed against mortality

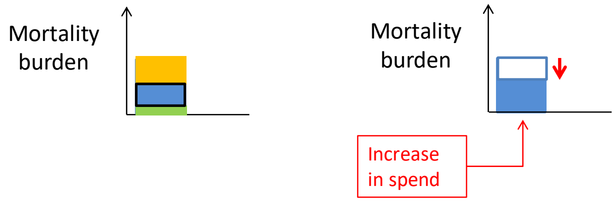

Deaths in the 2nd year could only be counted as averted if they didn't occur within that same year or the year after – averted for a minimum of 2 years

59

D. Life years associated with reductions in mortality.

There is more information we can draw from the existing data….

Let's have another look at the mortality data analysed in Claxton et al 2015:

At PCT level, PBC mortality for 3 consecutive years was averaged

PBC spend in a particular year was then regressed against mortality

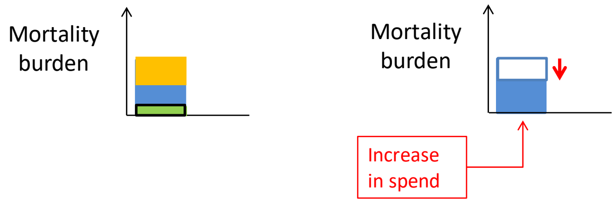

Deaths in the 1st year could only be counted as averted if they didn't occur within that same year, the year after, or the following – averted for a minimum of 3 years

60

D. Life years associated with reductions in mortality. Elicitation

D1. Of those patients that have seen their deaths averted by at least 3 years, 2 years and 1 year, what proportion is likely to return to (or exceed) the life expectancy of the general population of the same age and gender?

Please specify values between 0 and 1		My best guess for the value of this quantity is:	I am very certain (90% certain) that the true value for this quantity is … … higher than:	… lower than:
Circulatory				
For patients that had death averted for at least:	3 years	________	________	________
	2 years	________	________	________
	1 year	________	________	________
Respiratory				
For patients that had death averted for at least:	3 years	________	________	________
	2 years	________	________	________
	1 year	________	________	________

…

61

D. Life years associated with reductions in mortality. Elicitation

D2. Now consider only those patients who have <u>not</u> returned to, or exceeded, normal life expectancy. Please report your beliefs on their life expectancy as a proportion of the life expectancy in the general population of the same age and gender.

Please specify values between 0 and 1		My best guess for the value of this quantity is:	I am very certain (90% certain) that the true value for this quantity is … … higher than:	… lower than:
Circulatory				
For patients that had death averted for at least:	3 years	________	________	________
	2 years	________	________	________
	1 year	________	________	________
Respiratory				
For patients that had death averted for at least:	3 years	________	________	________
	2 years	________	________	________
	1 year	________	________	________

…

62

D. Life years associated with reductions in mortality. Clinical experts

	Experts from the particular clinical area*				
	#1	#2	#3	#4	#5
Gastrointestinal (1 yr)	1 (0.8,1)	0.1 (0.01,0.8)			
Gastrointestinal (2 yr)	0.9 (0.7,1)	0.05 (0.01,0.9)			
Gastrointestinal (3 yr)	0.8 (0.6,1)	0.025 (0.01,0.9)			

Distribution across all experts: Mode, Lower bound, Upper bound

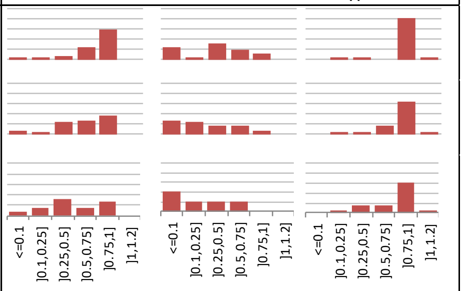

63

Please proceed to elicit quantity **D.**

64

Thank you so much for participating

marta.soares@york.ac.uk
karl.claxton@york.ac.uk
mark.sculpher@york.ac.uk

65
