# Supplementary material for: Health Opportunity Costs: Assessing the Implications of Uncertainty Using Elicitation Methods with Experts
Source: Med Decis Making. 2020 May 22;40(4):448–59. doi: 10.1177/0272989X20916450 (PMC7509606; doi:10.1177/0272989X20916450)
Supplement: Manuscript_expert_elicitation_HOC_MDM_4_Appendix3_online_supp – Supplemental material for Health Opportunity Costs: Assessing the Implications of Uncertainty Using Elicitation Methods with Experts [file Manuscript_expert_elicitation_HOC_MDM_4_Appendix3_online_supp.pdf]

Summary of the responses of clinical experts

Document date 01.06.2017

N = 28

Option(s) that best describe the clinical area(s) in which you are specialised:

		Confident that answers given expressed views and uncertainties			
**ID**	**Clinical area of specialisation**	**Section A**	**B**	**C**	**D**
1	Circulatory	Yes, Not sure	Yes, Not sure	Yes, Not sure	Yes, Not sure
7	Circulatory	Yes	Not sure	Not sure	Yes
8	Circulatory	Not sure	Not sure	Yes	Not sure
23	Circulatory	Yes	Yes	Yes	
9	Circulatory, neurological, musculoskeletal, other: rehabilitation	Yes	Yes	Yes	Yes
16	Respiratory	Yes	Yes	Yes	Yes
19	Respiratory, primary care	Not sure	Not sure	Yes	Not sure
14	GI	Not sure	Not sure	Yes	Not sure
20	GI	Not sure	Yes	Yes	missing
24	GI	Not sure	Not sure	missing	missing
26	Neurology	Yes	Not sure	missing	Not sure
10	Endocrinology	Not sure	Not sure	Not sure	Not sure
18	Endocrinology	Yes	Not sure	Yes	Yes
4	Endocrinology, other: general medicine	Not sure	Not sure	Not sure	Not sure
12	Mental health	Yes	Not sure	Not sure	Not sure, No
27	Mental health	Not sure	Not sure	Not sure	Not sure
28	Mental health	Yes	No	Yes	Yes
6	Primary care	Yes	Not sure	Yes	Yes
17	Primary care	No	No	missing	missing
15	Primary care, other: pharmacist	Yes	Yes	Yes	Yes
2	Other: anaesthetics	Yes	Not sure	Yes	Yes
3	Other: public health, CCG gov body member	Yes	Not sure	Not sure	Yes
11	Other: public health and geriatric medicine	Yes	Yes	missing	Not sure
21	Other: ophtalmology	Not sure	Not sure	Not sure	Not sure
25	Other: radiology	Not sure	Not sure	Not sure	missing
5	No clinical expertise	Yes	Not sure	Yes	Yes
13	No clinical expertise	Yes	Not sure	Yes	Yes
22	No clinical expertise	Not sure	No	Not sure	Not sure

Section A

A1. On average, for how many more years (beyond the year of increased expenditure) would you expect disease-specific mortality rates to be reduced in each of the specific disease areas listed below?

	**Experts from the particular clinical area***					**Distribution across all experts**		
	**#1**	**#2**	**#3**	**#4**	**#5**	**Mode**	**Lower bound**	**Upper bound**
Circulatory	3 (2,6)	5 (3,10)	10 (5,25)	3 (2,10)	15 (5,20)			
Respiratory	1 (0,3)	3 (2,6)						
Gastrointestinal	20 (5,40)	1 (0.5,3)	3.5 (0.5,5)					
Neurological	15 (10,45)	3 (1,10)						
Endocrinology	2 (0,3)	1 (0,4)	5 (3,12)					
Others w mortality								

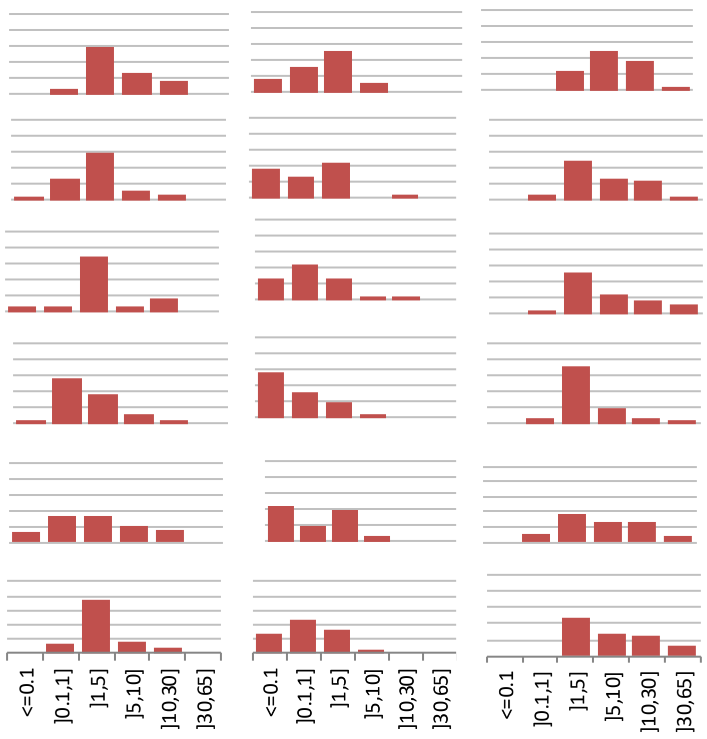

*ID of experts from the specific clinical areas – circulatory 1, 7, 8, 9, 23; respiratory 16,19; Gastrointestinal 14, 20, 24; neurological 9, 26; endocrinology; 4, 10, 18.

A2. From an increase in expenditure in a particular year, how do reductions in mortality rates in subsequent years compare to the reduction observed in the first year?

	Experts from the particular clinical area*					Distribution across all experts		
	#1	#2	#3	#4	#5	Mode	Lower bound	Upper bound
Circulatory (2 yr)	1.5 (1,2)	0.75 (0.5,0.8)	0.5 (0.2,1)	0.5 (0.25,0.75)	1.5 (1.1,1.7)			
Circulatory (3 yr)	1 (0.5,1.5)	0.5 (0.3,0.7)	0.3 (0.1,0.5)	0.25 (0.2,0.3)	1.4 (1.1,1.7)			
Circulatory (4 yr)	1 (0.5,1.5)	0.4 (0.2,0.5)	0.2 (0.05,0.5)	0.1 (0.05,0.2)	1.4 (1.1,1.7)			
Respiratory (2 yr)	0.6 (0.1,1)	1 (-0.2,2)						
Respiratory (3 yr)	0.3 (0.1,0.6)	0.8 (-0.2,1.5)						
Respiratory (4 yr)	0.1 (0,0.5)	0.2 (0.5,1.5)						
Gastrointestinal (2 yr)	1.2 (0.4,1.5)	0.1 (0.05,0.3)	0.75 (0.1,1)					
Gastrointestinal (3 yr)	1.4 (0.3,1.5)	0 (-0.1,0.3)	0.5 (0.05,1)					
Gastrointestinal (4 yr)	1.6 (0.2,1.5)	0 (-0.1,0.3)	0.2 (0.001,0.5)					

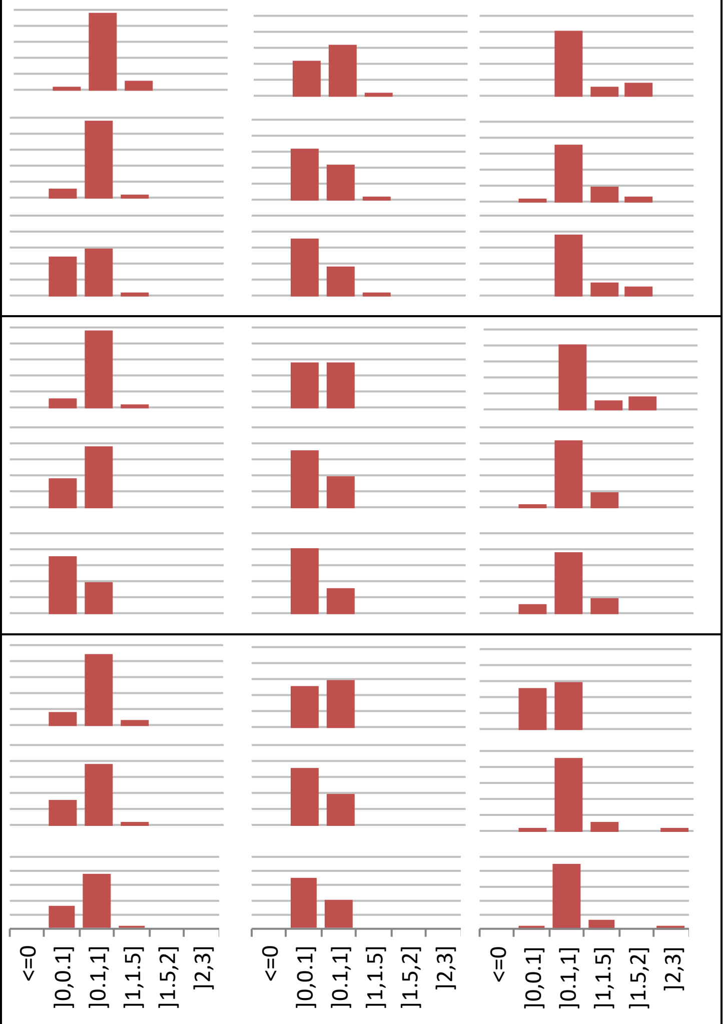

	Experts from the particular clinical area*					Distribution across all experts		
	#1	#2	#3	#4	#5	Mode	Lower bound	Upper bound
Neurological (2 yr)	0.98 (0.97,0.99)	0.5 (0.1,0.8)						
Neurological (3 yr)	0.95 (0.9,0.98)	0.2 (0.05,0.6)						
Neurological (4 yr)	0.92 (0.9,0.95)	0.1 (0.01,0.3)						
Endocrinology (2 yr)	0.9 (0.4,1)	0.5 (0.2,1)	1 (0.5,2)					
Endocrinology (3 yr)	0.95 (0.4,1)	0.05 (0,0.5)	1.5 (0.5,3)					
Endocrinology (4 yr)	0 (0,0)	0.01 (0,0.2)	1 (0.5,1.5)					
Others w mortality (2 yr)								
Others w mortality (3 yr)								
Others w mortality (4 yr)								

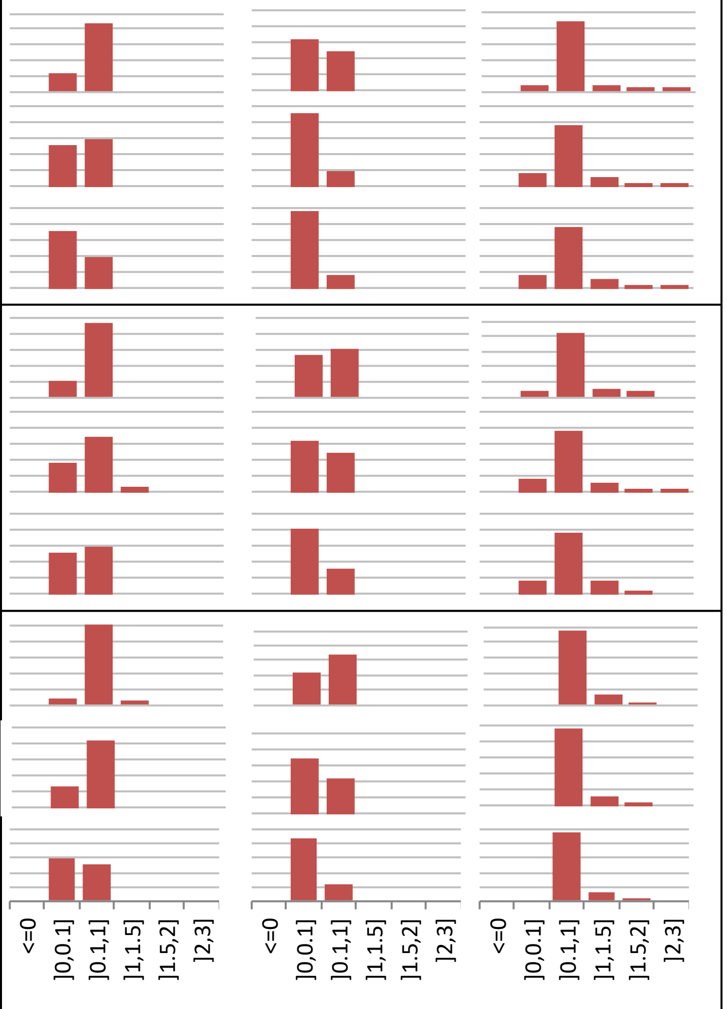

*ID of experts from the specific clinical areas – circulatory 1, 7, 8, 9, 23; respiratory 16,19; Gastrointestinal 14, 20, 24; neurological 9, 26; endocrinology; 4, 10, 18.

Section B

B1. How do the effects of increased expenditure on health burden compare with its effects on mortality burden?

	Experts from the particular clinical area*					Distribution across all experts		
	#1	#2	#3	#4	#5	Mode	Lower bound	Upper bound
Circulatory (1 yr)	0.8 (0.2,1.2)	2 (1,2.5)	1 (0.2,2)	1 (0.7,1.3)	1.4 (1,1.5)			
Circulatory (2 yr)	0.9 (0.2,1.2)	1.7 (1,2)	1 (0.2,2)	1.2 (0.9,1.5)	1.3 (1,1.5)			
Circulatory (3 yr)	0.8 (0.2,1.1)	1.3 (0.8,1.8)	1 (0.2,2)	1.5 (1.3,1.7)	1.3 (1,1.5)			
Circulatory (4 yr)	0.6 (0.1,0.9)	1 (0.5,1.5)	1 (0.2,2)	1.7 (1.5,2)	1.2 (1,1.5)			
Respiratory (1 yr)	0.7 (0.5,1.3)	1.5 (0.8,2)						
Respiratory (2 yr)	0.8 (0.5,1.5)	1 (0.5,1.5)						
Respiratory (3 yr)	0.9 (0.6,2)	0.5 (0.3,1.5)						
Respiratory (4 yr)	1 (0.6,2.5)	0.2 (0.1,1.5)						

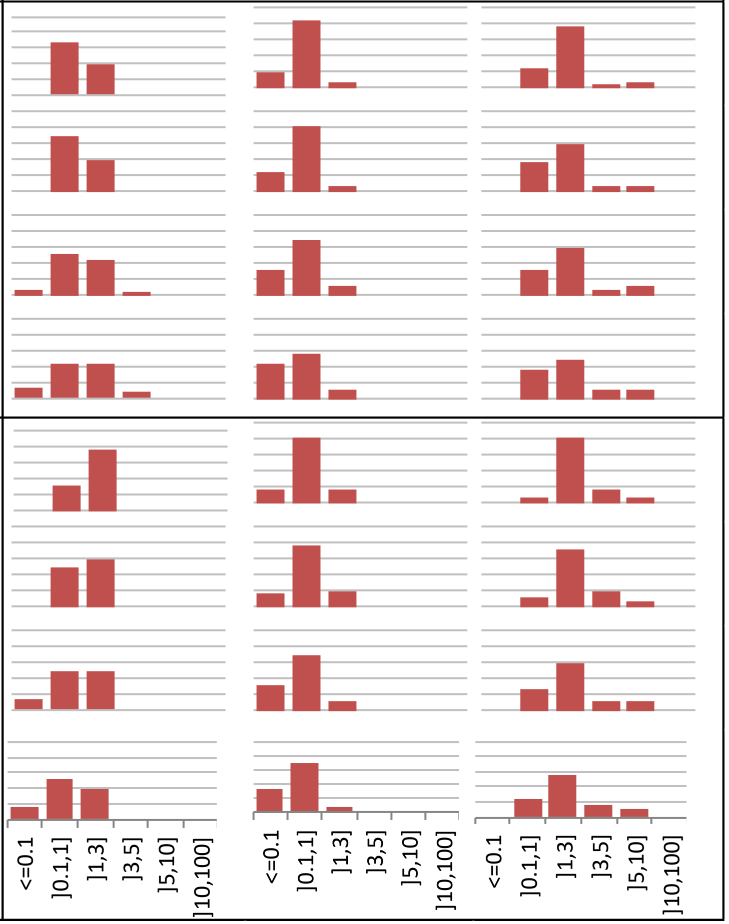

*ID of experts from the specific clinical areas – circulatory 1, 7, 8, 9, 23; respiratory 16,19; Gastrointestinal 14, 20, 24; neurological 9, 26; endocrinology; 4, 10, 18.

	Experts from the particular clinical area*					Distribution across all experts		
	#1	#2	#3	#4	#5	Mode	Lower bound	Upper bound
Gastrointestinal (1 yr)	1.5 (1,2)	0.5 (0.1,2)	0.5 (0.01,0.8)			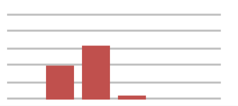	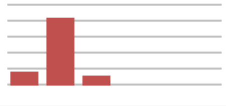	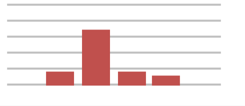
Gastrointestinal (2 yr)	1.4 (1,3)	1 (0.05,5)	0.4 (0.01,0.7)			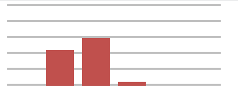	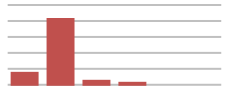	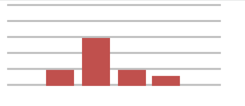
Gastrointestinal (3 yr)	1.3 (1,4)	1 (0.05,5)	0.2 (0.01,0.5)			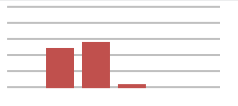	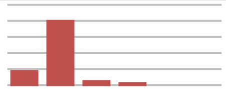	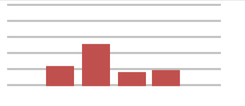
Gastrointestinal (4 yr)	1 (1,5)	1 (0.05,5)	0.1 (0.01,0.2)			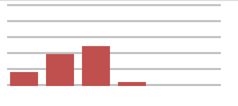	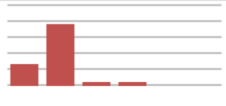	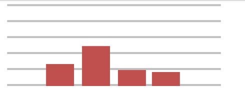
Neurological (1 yr)	3 (2.5,5)	0.4 (0.1,0.8)				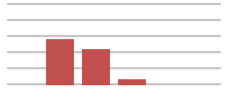	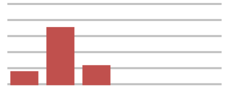	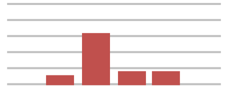
Neurological (2 yr)	2 (1,4)	0.2 (0.01,0.6)				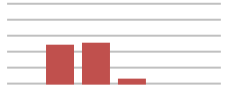	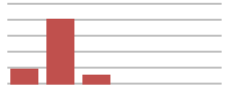	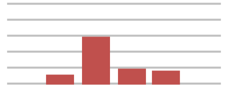
Neurological (3 yr)	1.5 (1,3)	0.05 (0.01,0.3)				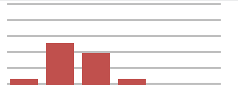	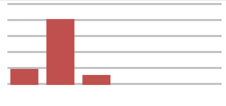	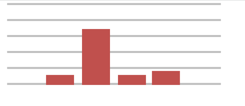
Neurological (4 yr)	1.25 (1,2)	0 (0,0.2)				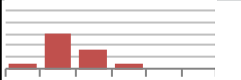	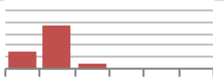	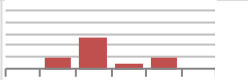

*ID of experts from the specific clinical areas – circulatory 1, 7, 8, 9, 23; respiratory 16,19; Gastrointestinal 14, 20, 24; neurological 9, 26; endocrinology; 4, 10, 18.

	Experts from the particular clinical area*					Distribution across all experts		
	#1	#2	#3	#4	#5	Mode	Lower bound	Upper bound
Endocrinology (1 yr)	0.1 (0,0.3)	2 (1,6)	1 (0.5,1.5)					
Endocrinology (2 yr)	0.1 (0,0.3)	2.5 (1,7)	1 (0.5,1)					
Endocrinology (3 yr)	0.1 (0,0.3)	3 (1,7)	1 (0.5,1)					
Endocrinology (4 yr)	0 (0,0)	4 (1.5,8)	1 (0.5,1)					
Others w mortality (1 yr)								
Others w mortality (2 yr)								
Others w mortality (3 yr)								
Others w mortality (4 yr)								

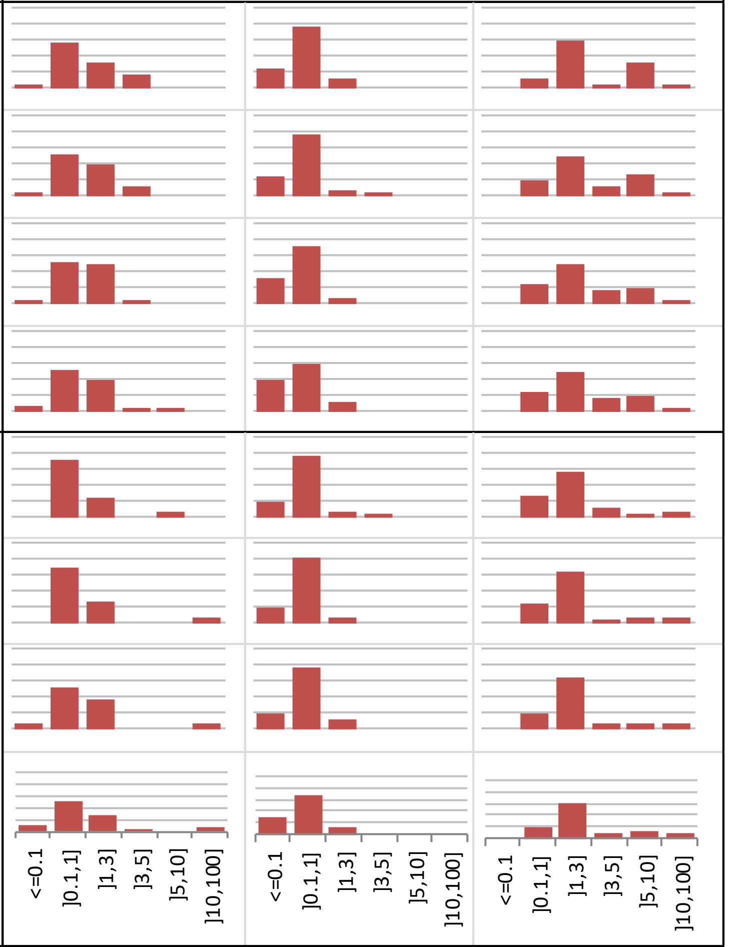

*ID of experts from the specific clinical areas – circulatory 1, 7, 8, 9, 23; respiratory 16,19; Gastrointestinal 14, 20, 24; neurological 9, 26; endocrinology; 4, 10, 18.

Section C

C1. How do reductions in health burden (quality-adjusted life-years lost due to disease) from an increase in NHS expenditure in the following disease areas compare to reductions in health burden from increased expenditure across all disease areas with measurable mortality effects?

	Experts from the particular clinical area*					Distribution across all experts		
	#1	#2	#3	#4	#5	Mode	Lower bound	Upper bound
Mental Health (1 yr)	3 (1,5)	0.8 (0.5,1.3)	1.5 (0.5,3)					
Mental Health (2 yr)	2 (1,4)	0.7 (0.4,1)	1.3 (0.5,2)					
Mental Health (3 yr)	1.5 (1,3)	0.5 (0.3,1)	1.2 (0.5,2)					
Mental Health (4 yr)	1 (0.75,2.5)	0.3 (0.1,0.7)	1.1 (0.5,2)					
Musculoskeletal (1 yr)	4 (2,6)							
Musculoskeletal (2 yr)	3 (1.5,4.5)							
Musculoskeletal (3 yr)	2.5 (1.5,3.5)							
Musculoskeletal (4 yr)	2 (1.25,2.75)							

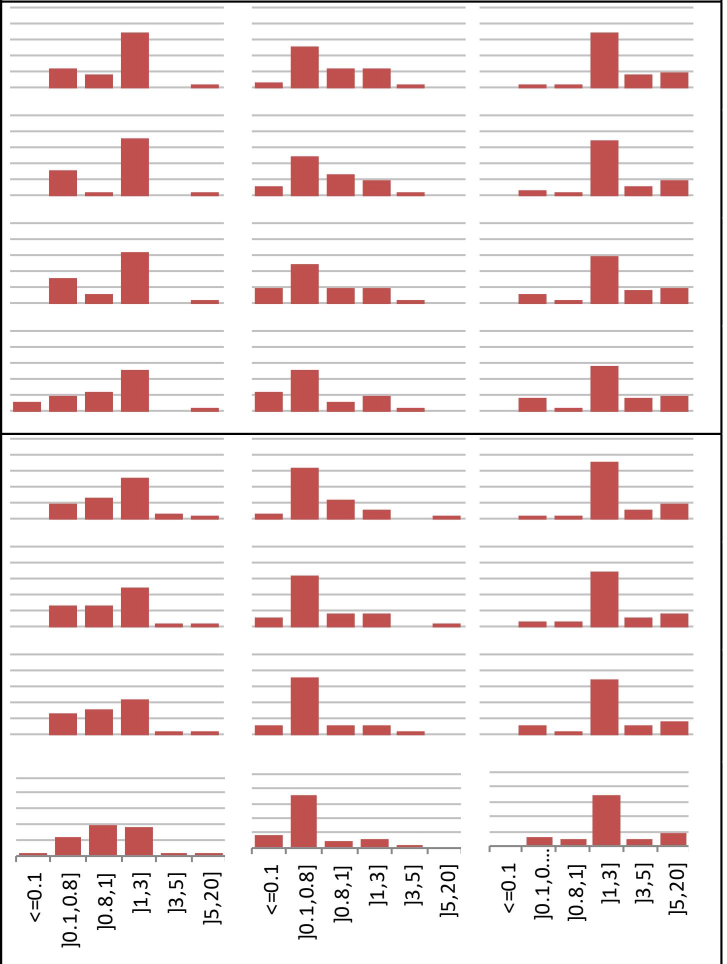

*ID of experts from the specific clinical areas – mental health 12, 27, 28; musculoskeletal 9.

	Experts from the particular clinical area*					Distribution across all experts		
	#1	#2	#3	#4	#5	Mode	Lower bound	Upper bound
Others (1 yr)								
Others (2 yr)								
Others (3 yr)								
Others (4 yr)								

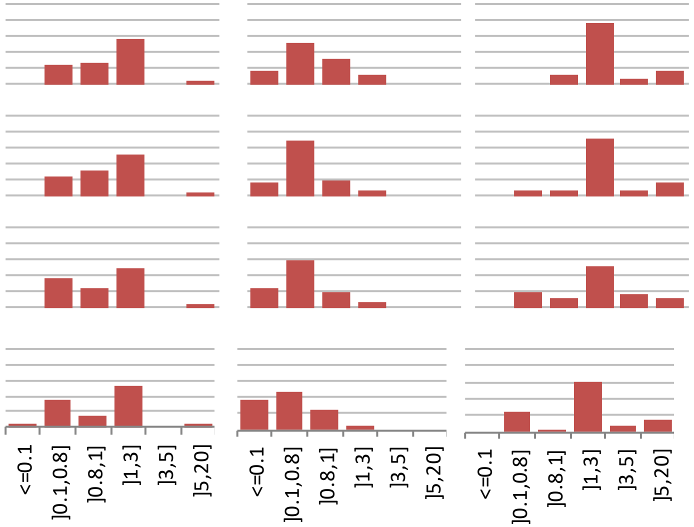

*ID of experts from the specific clinical areas – mental health 12, 27, 28; musculoskeletal 9.

__Section D__

D1. Of those patients that have seen their deaths averted by at least 3 years, 2 years and 1 year, what proportion are likely to return to (or exceed) the life expectancy of the general population of the same age and gender?

	Experts from the particular clinical area*				
	#1	#2	#3	#4	#5
Circulatory (3 yr)	0.5 (0,0.8)	0.5 (0.25,0.75)	0.3 (0,0.8)	0.05 (0.01,0.1)	
Circulatory (2 yr)	0.4 (0,0.9)	0.35 (0.25,0.5)	0.2 (0,0.6)	0.02 (0.01,0.05)	
Circulatory (1 yr)	0.3 (0,0.6)	0.2 (0.1,0.25)	0.1 (0,0.5)	0 (0,0.01)	
Respiratory (3 yr)	0.8 (0.5,0.9)	0.05 (0,0.1)			
Respiratory (2 yr)	0.6 (0.3,0.9)	0.05 (0,0.1)			
Respiratory (1 yr)	0.2 (0,0.7)	0.05 (0,0.1)			
Gastrointestinal (3 yr)	1 (0.8,1)	0.1 (0.01,0.8)			
Gastrointestinal (2 yr)	0.9 (0.7,1)	0.05 (0.01,0.9)			
Gastrointestinal (1 yr)	0.8 (0.6,1)	0.025 (0.01,0.9)			

Distribution across all experts

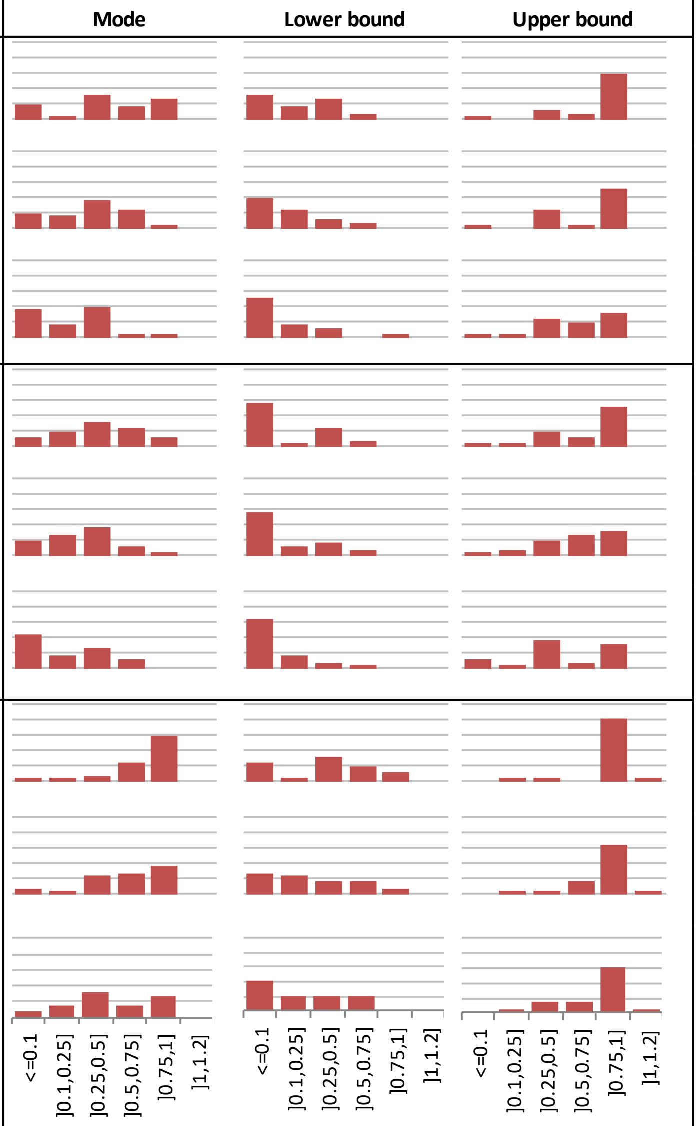

	Experts from the particular clinical area*					Distribution across all experts		
	#1	#2	#3	#4	#5	Mode	Lower bound	Upper bound
Neurological (3 yr)	1 (0.8,1)	0.5 (0.2,0.8)						
Neurological (2 yr)	0.9 (0.8,1)	0.4 (0.1,0.6)						
Neurological (1 yr)	0.8 (0.7,0.9)	0.3 (0.1,0.5)						
Endocrinology (3 yr)	0.1 (0,0.2)	0.9 (0.3,1)	0.4 (0.1,0.6)					
Endocrinology (2 yr)	0.08 (0,0.18)	0.6 (0.2,1)	0.2 (0.1,0.6)					
Endocrinology (1 yr)	0.06 (0,0.16)	0.3 (0.1,0.8)	0.1 (0.1,0.6)					
Others w mortality (3 yr)								
Others w mortality (2 yr)								
Others w mortality (1 yr)								

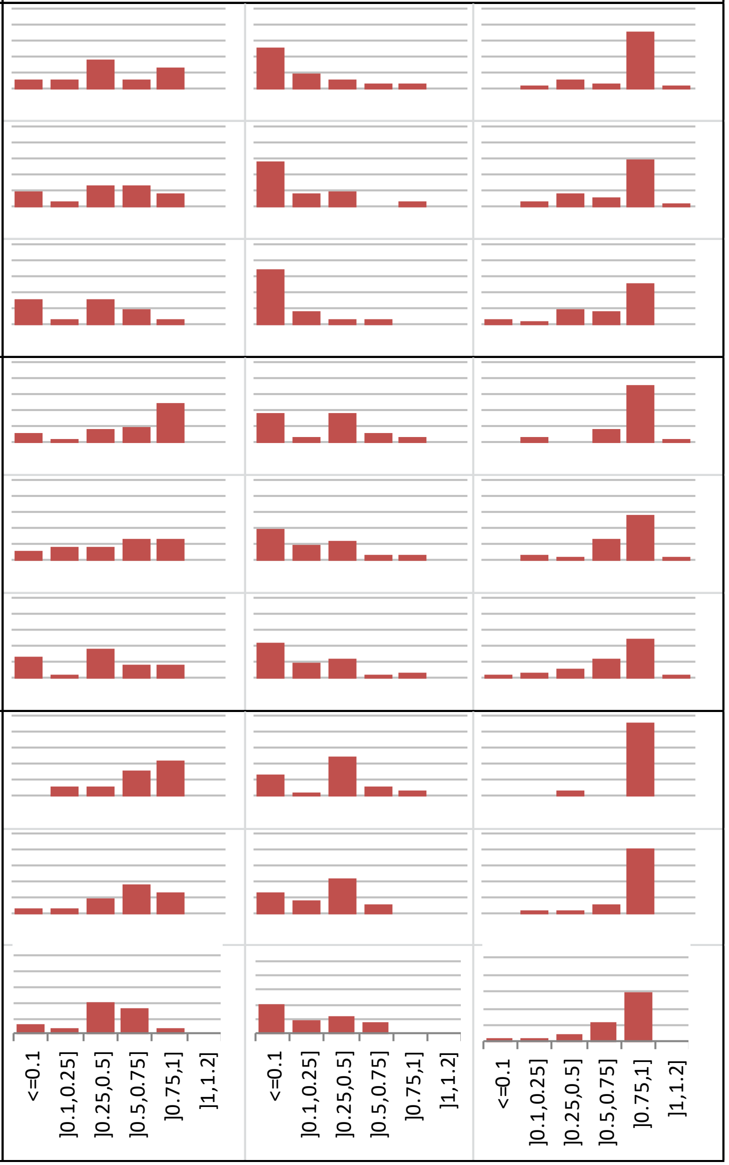

*ID of experts from the specific clinical areas – circulatory 1, 7, 8, 9, 23; respiratory 16,19; Gastrointestinal 14, 20, 24; neurological 9, 26; endocrinology; 4, 10, 18.

D2. Now consider only those patients who have not returned to, or exceeded, normal life expectancy. Please report your beliefs on their life expectancy as a proportion of the life expectancy in the general population of the same age and gender:

	Experts from the particular clinical area*				
	#1	#2	#3	#4	#5
Circulatory (3 yr)	0.6 (0.1,0.9)	0.75 (0.5,0.9)	0.7 (0.5,0.9)	0.8 (0.6,0.9)	
Circulatory (2 yr)	0.7 (0.1,1)	0.6 (0.25,0.75)	0.7 (0.5,0.9)	0.7 (0.5,0.9)	
Circulatory (1 yr)	0.8 (0.1,1.1)	0.4 (0.3,0.5)	0.7 (0.5,0.9)	0.5 (0.4,0.6)	
Respiratory (3 yr)	0.4 (0,0.9)	0.1 (0.05,0.2)			
Respiratory (2 yr)	0.3 (0,0.9)	0.1 (0.05,0.2)			
Respiratory (1 yr)	0.2 (0,0.9)	0.1 (0.05,0.2)			
Gastrointestinal (3 yr)	0.9 (0.6,0.95)	0.9 (0.7,0.95)			
Gastrointestinal (2 yr)	0.8 (0.6,0.8)	0.8 (0.6,0.95)			
Gastrointestinal (1 yr)	0.7 (0.6,0.6)	0.7 (0.3,0.95)			

Distribution across all experts

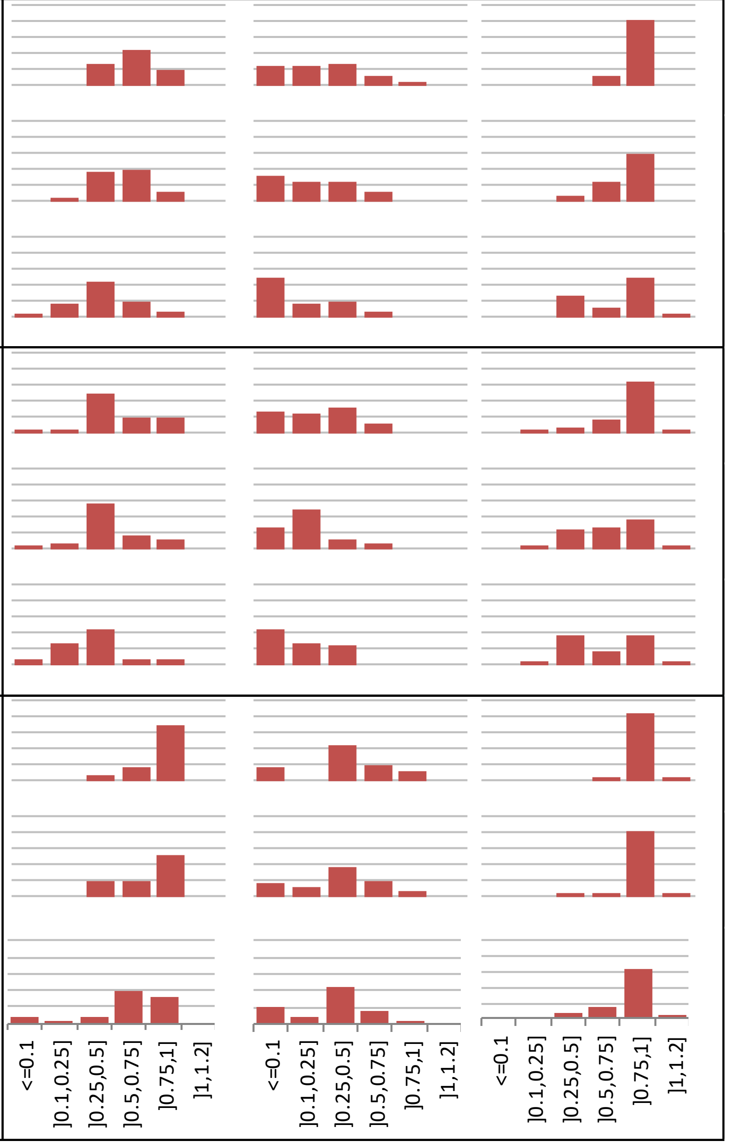

	Experts from the particular clinical area*					Distribution across all experts		
	#1	#2	#3	#4	#5	Mode	Lower bound	Upper bound
Neurological (3 yr)	0.9 (0.8,1)	0.6 (0.3,0.9)						
Neurological (2 yr)	0.85 (0.8,0.9)	0.5 (0.2,0.8)						
Neurological (1 yr)	0.8 (0.7,0.9)	0.4 (0.1,0.6)						
Endocrinology (3 yr)	0.8 (0.6,0.9)	0.7 (0.4,0.95)	0.5 (0.05,1)					
Endocrinology (2 yr)	0.75 (0.6,0.9)	0.4 (0.2,0.6)	0.5 (0.05,1)					
Endocrinology (1 yr)	0.7 (0.6,0.9)	0.2 (0.05,0.4)	0.5 (0.05,1)					
Others w mortality (3 yr)								
Others w mortality (2 yr)								
Others w mortality (1 yr)								

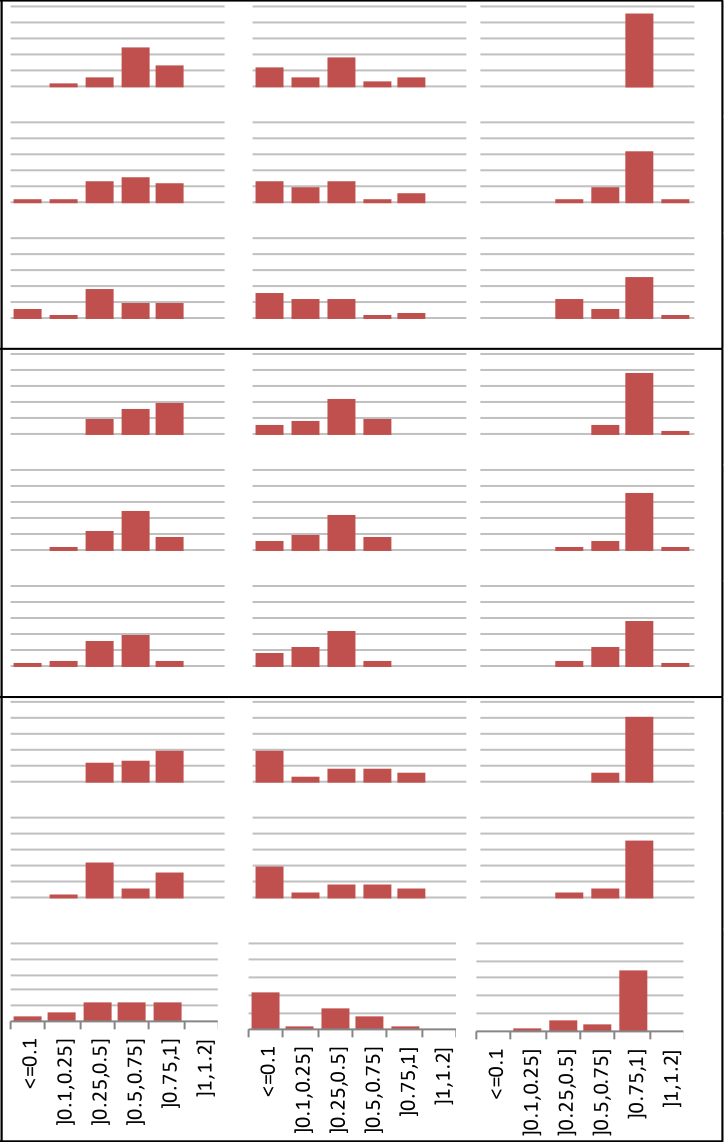

*ID of experts from the specific clinical areas – circulatory 1, 7, 8, 9, 23; respiratory 16,19; Gastrointestinal 14, 20, 24; neurological 9, 26; endocrinology; 4, 10, 18.

Data from individual experts

A1

	**1**	**7**	**8**	**23**	**9**	**16**	**19**	**14**	**20**	**24**	**26**	**10**	**18**	**4**
A1.1	3<br>(2,6)	5<br>(3,10)	10<br>(5,25)	15<br>(5,20)	3<br>(2,10)	2<br>(1,5)	15<br>(10,30)	20<br>(10,40)	1<br>(0.5,3)	2<br>(0.1,2.5)	3<br>(1,10)	3<br>(0.01,10)	5<br>(3,10)	6<br>(1,12)
A1.2	4<br>(2,6)	3<br>(2,5)	1<br>(0.01,10)	10<br>(4,12)	2<br>(1.5,7)	1<br>(0.01,3)	3<br>(2,6)	30<br>(15,40)	1<br>(0.5,3)	3<br>(0.5,3.8)	5<br>(1,10)	5<br>(0.01,15)	4<br>(2,15)	1<br>(0.01,3)
A1.3	5<br>(3,10)	2<br>(1,5)	2<br>(0.01,10)	5<br>(2,7)	20<br>(15,35)	2<br>(0.01,4)	6<br>(2,20)	20<br>(5,40)	1<br>(0.5,3)	3.5<br>(0.5,5)	2<br>(1,10)	4<br>(0.01,15)	4<br>(2,10)	2<br>(0.01,4)
A1.4	4<br>(3,10)	1<br>(0.01,2)	1<br>(0.01,5)	2<br>(1,4)	15<br>(10,45)	0.5<br>(0.01,3)	10<br>(5,20)	5<br>(2,7)	1<br>(0.5,3)	0.5<br>(0.1,0.8)	3<br>(1,10)	1<br>(0.01,4)	2<br>(1,5)	1<br>(0.01,3)
A1.5	6<br>(4,10)	5<br>(3,10)	0.1<br>(0.01,2)	15<br>(5,20)	12<br>(2,20)	1<br>(0.01,2.5)	12<br>(6,24)	10<br>(5,40)	1<br>(0.5,3)	0.5<br>(0.1,0.8)	6<br>(1,12)	1<br>(0.01,4)	5<br>(3,12)	2<br>(0.01,3)
A1.6	2<br>(1,3)	3<br>(2,5)	5<br>(0.01,20)	5<br>(2,8)	15<br>(1,65)	2.5<br>(1,5)	6<br>(3,12)	10<br>(5,30)	3<br>(1,10)	6<br>(3,12)	2<br>(0.01,10)	1<br>(0.01,3)	1<br>(0.5,5)	2<br>(0.01,3)

	**12**	**27**	**28**	**6**	**17**	**15**	**2**	**3**	**11**	**21**	**25**	**5**	**13**	**22**
A1.1	3<br>(0.5,5)	10<br>(3,20)	2<br>(0.1,4)	10<br>(2,12)	10<br>(1,20)	5<br>(2,10)	6<br>(2,10)	5<br>(2,10)	1<br>(0.01,2)	5<br>(1,10)	5<br>(2,10)	7<br>(3,12)	3<br>(1,10)	15<br>(10,25)
A1.2	2<br>(0.25,5)	10<br>(3,20)	1<br>(0.1,3)	2<br>(1,3)	3<br>(0.5,5)	10<br>(2,20)	2<br>(1,5)	5<br>(2,10)	0.1<br>(0.01,1)	2<br>(0.01,5)	0.5<br>(0.1,1)	5<br>(4,8)	1<br>(0.01,5)	15<br>(5,30)
A1.3	3<br>(0.25,5)	2<br>(1,5)	1<br>(0.1,3)	2<br>(1,5)		20<br>(1,25)	2<br>(1,4)	10<br>(5,20)	0.1<br>(0.01,1)	5<br>(1,10)	2<br>(1,3)	3<br>(2,5)	0.1<br>(0.01,5)	20<br>(10,35)
A1.4	1<br>(0.1,3)	3<br>(1,5)	1<br>(0.1,3)	2<br>(1,3)	1<br>(0.01,5)	2<br>(1,5)	1<br>(0.01,8)	1<br>(0.01,4)	2<br>(1,3)	1<br>(0.01,5)	0.3<br>(0.01,0.5)	6<br>(2,8)	0.1<br>(0.01,3)	10<br>(5,20)
A1.5	1<br>(0.1,3)	10<br>(3,20)	1<br>(0.1,3)	5<br>(2,6)	5<br>(1,12)	5<br>(2,10)	3<br>(1,6)	3<br>(1,7)	1<br>(0.01,1)	1<br>(0.01,5)	0.1<br>(0.01,0.5)	8<br>(4,10)	0.1<br>(0.01,3)	25<br>(10,40)
A1.6	2<br>(0.5,5)	5<br>(2,20)	2<br>(0.1,4)	5<br>(2,10)	5<br>(0.01,10)	3<br>(1,20)	10<br>(4,40)	2<br>(1,4)	5<br>(1,10)	5<br>(1,10)	3<br>(1,5)	2<br>(1,4)	1<br>(0.01,5)	25<br>(10,50)

A2

	1	7	8	23	9	16	19	14	20	24	26	10	18	4
A2.1.2	1.5 (1,2)	0.75 (0.5,0.8)	0.5 (0.2,1)	1.5 (1.1,1.7)	0.5 (0.25,0.75)	0.8 (0.5,1)	0.5 (0.1,1.5)	1 (0.8,1.4)	0.1 (0.05,0.3)		0.3 (0.1,0.8)	0.5 (0.2,1)	1.25 (0.5,2)	0.85 (0.5,1)
A2.1.3	1 (0.5,1.5)	0.5 (0.3,0.7)	0.3 (0.1,0.5)	1.4 (1.1,1.7)	0.25 (0.2,0.3)	0.4 (0.01,0.8)	0.4 (0.1,1.5)	1 (0.7,1.5)	0.1 (0.01,0.3)		0.1 (0.001,0.5)	0.25 (0.1,0.5)	1 (0.5,1.5)	
A2.1.4	1 (0.5,1.5)	0.4 (0.2,0.5)	0.2 (0.05,0.5)	1.4 (1.1,1.7)	0.1 (0.05,0.2)	0.2 (0.01,0.5)	0.4 (0.01,1.5)	1 (0.6,1.6)	0.1 (0.01,0.3)		0.05 (0.001,0.2)	0.1 (0.01,0.3)	0.9 (0.25,1.25)	
A2.2.2	1.5 (0.5,2)	0.4 (0.25,0.6)	0.3 (0.01,0.5)	1 (0.9,1.4)	0.4 (0.1,0.5)	0.6 (0.1,1)	1 (0.01,2)	0.8 (0.6,1.2)	0.1 (0.05,0.3)		0.5 (0.2,0.9)	0.8 (0.4,1)	0.5 (0.25,2)	0.7 (0.4,0.8)
A2.2.3	1 (0.5,1.5)	0.3 (0.15,0.5)	0.1 (0.01,0.2)	0.8 (0.5,0.9)	0.1 (0.05,0.15)	0.3 (0.1,0.6)	0.8 (0.01,1.5)	0.7 (0.5,1.3)	0.1 (0.01,0.3)		0.3 (0.05,0.6)	0.5 (0.2,0.8)	0.2 (0.1,1)	0.1 (0.01,0.2)
A2.2.4	1 (0.5,1.5)	0.2 (0.01,0.4)	0.1 (0.01,0.2)	0.7 (0.4,0.9)	0.05 (0.01,0.1)	0.1 (0.01,0.5)	0.2 (0.5,1.5)	0.6 (0.4,1.3)	0.1 (0.01,0.3)		0.1 (0.001,0.4)	0.2 (0.1,0.4)	0.1 (0.05,0.9)	0.1 (0.01,0.2)
A2.3.2	0.5 (0.5,1.5)	0.35 (0.2,0.5)	0.5 (0.01,0.9)	0.9 (0.7,1)	0.9 (0.85,0.95)	0.8 (0.5,1)	0.5 (0.1,0.7)	1.2 (0.4,1.5)	0.1 (0.05,0.3)	0.75 (0.1,1)	0.5 (0.1,0.8)	0.5 (0.2,1)	1.5 (0.5,3)	0.5 (0.2,0.7)
A2.3.3	1 (0.01,1.5)	0.15 (0.1,0.25)	0.1 (0.01,0.5)	0.8 (0.5,0.9)	0.85 (0.8,0.9)	0.4 (0.01,0.8)	0.3 (0.1,0.5)	1.4 (0.3,1.5)	0.1 (0.01,0.3)	0.5 (0.05,1)	0.1 (0.05,0.5)	0.25 (0.1,0.6)	1 (0.5,3)	0.5 (0.2,0.7)
A2.3.4	0.5 (0.01,1.5)	0.1 (0.01,0.25)	0.1 (0.01,0.2)	0.5 (0.2,0.7)	0.8 (0.75,0.85)	0.2 (0.01,0.5)	0.3 (0.1,0.5)	1.6 (0.2,1.5)	0.1 (0.01,0.3)	0.2 (0.001,0.5)	0.1 (0.01,0.2)	0.15 (0.05,0.3)	1 (0.5,3)	0.1 (0.01,0.2)
A2.4.2	1 (0.01,2)	0.5 (0.2,0.7)	0.1 (0.01,0.9)	0.5 (0.3,0.7)	0.98 (0.97,0.99)	0.5 (0.1,0.8)	0.5 (0.1,0.7)	0.7 (0.4,1.5)	0.1 (0.05,3)	0.1 (0.01,0.01)	0.5 (0.1,0.8)	0.4 (0.2,1)	0.5 (0.1,1)	0.5 (0.2,0.7)
A2.4.3	1 (0.01,2)	0.1 (0.01,0.25)	0.1 (0.01,0.1)	0.2 (0.1,0.3)	0.95 (0.9,0.98)	0.25 (0.01,0.5)	0.3 (0.1,0.5)	0.5 (0.3,1.5)	0.1 (0.01,3)	0.1 (0.01,0.01)	0.2 (0.05,0.6)	0.1 (0.01,0.5)	0.2 (0.1,1)	0.1 (0.01,0.2)
A2.4.4	0.5 (0.01,2)	0.1 (0.01,0.25)	0.1 (0.01,0.1)	0.1 (0.01,0.2)	0.92 (0.9,0.95)	0.1 (0.01,0.2)	0.3 (0.1,0.5)	0.2 (0.2,1.5)	0.1 (0.01,3)	0.1 (0.01,0.01)	0.1 (0.01,0.3)	0.01 (0.01,0.3)	0.15 (0.05,0.5)	0.1 (0.01,0.2)
A2.5.2	1 (0.5,2)	0.75 (0.5,0.8)	0.1 (0.01,0.1)	0.8 (0.6,1)	0.8 (0.75,0.85)	0.6 (0.2,1)	0.5 (0.1,0.7)	0.9 (0.2,1.2)	0.1 (0.05,0.3)	0.1 (0.01,0.01)	0.5 (0.1,0.8)	0.5 (0.2,1)	1 (0.5,2)	0.9 (0.4,1)
A2.5.3	1.5 (0.01,2)	0.5 (0.3,0.7)	0.1 (0.01,0.1)	0.7 (0.5,0.8)	0.7 (0.65,0.75)	0.4 (0.1,0.9)	0.3 (0.1,0.5)	0.7 (0.2,1.2)	0.05 (0.01,0.3)	0.1 (0.01,0.01)	0.3 (0.1,0.6)	0.05 (0.01,0.5)	1.5 (0.5,3)	0.95 (0.4,1)
A2.5.4	1 (0.01,2)	0.3 (0.15,0.5)	0.1 (0.01,0.1)	0.6 (0.3,0.7)	0.6 (0.5,0.7)	0.2 (0.01,0.5)	0.3 (0.1,0.5)	0.3 (0.1,1.2)	0.025 (0.01,0.3)	0.1 (0.01,0.01)	0.2 (0.05,0.4)	0.01 (0.01,0.2)	1 (0.5,1.5)	0.1 (0.01,0.2)
A2.6.2	1.5 (0.5,2)	0.4 (0.2,0.7)	0.1 (0.01,0.3)	0.8 (0.7,0.9)	0.99 (0.98,1)	0.8 (0.5,1.2)	0.5 (0.1,0.7)	0.5 (0.1,0.7)	0.1 (0.05,0.3)	1 (0.5,1.5)	0.5 (0.1,0.8)	0.5 (0.2,1)	0.5 (0.25,1)	0.7 (0.4,1)
A2.6.3	1 (0.01,2)	0.3 (0.15,0.6)	0.1 (0.01,0.3)	0.4 (0.1,0.6)	0.99 (0.97,1)	0.8 (0.5,1)	0.3 (0.1,0.5)	0.3 (0.1,0.7)	0.05 (0.01,0.2)	1 (0.3,1.5)	0.1 (0.05,0.6)	0.05 (0.01,0.4)	0.25 (0.15,1)	0.7 (0.4,1)
A2.6.4	0.5 (0.01,2)	0.2 (0.1,0.5)	0.1 (0.01,0.3)	0.1 (0.01,0.2)	0.98 (0.95,1)	0.6 (0.3,1)	0.3 (0.1,0.5)	0.1 (0.1,0.7)	0.025 (0.001,0.2)	1 (0.3,1.2)	0.1 (0.01,0.2)	0.01 (0.01,0.2)	0.25 (0.15,1)	0.1 (0.01,0.2)

	12	27	28	6	17	15	2	3	11	21	25	5	13	22
A2.1.2	0.5 (0.1,0.9)	0.8 (0.4,1.1)	0.5 (0.1,0.7)	0.9 (0.7,1)	0.9 (0.01,2)	0.3 (0.1,0.9)	0.7 (0.2,0.9)	0.5 (0.25,0.75)	0.25 (0.1,0.5)	0.4 (0.1,0.8)	0.5 (0.25,0.65)	0.8 (0.4,0.9)	0.5 (0.01,1)	0.6 (0.4,1)
A2.1.3	0.25 (0.1,0.75)	0.6 (0.4,1.3)	0.2 (0.01,0.5)	0.8 (0.6,0.9)	0.8 (0.01,2)	0.2 (0.1,0.7)	0.4 (0.01,0.8)	0.5 (0.25,0.75)	0.1 (0.01,0.1)	0.2 (0.1,0.4)	0.2 (0.1,0.3)	0.6 (0.4,0.8)	0.25 (0.01,1)	0.5 (0.3,1)
A2.1.4	0.1 (0.1,0.5)	0.2 (0.1,1.1)	0.1 (0.01,0.4)	0.7 (0.4,0.8)	0.7 (0.01,2)	0.1 (0.01,0.5)	0.2 (0.01,0.8)	0.5 (0.25,0.75)	0.1 (0.01,0.2)	0.1 (0.05,0.2)	0.1 (0.02,0.2)	0.4 (0.2,0.5)	0.1 (0.01,1)	0.4 (0.3,0.9)
A2.2.2	0.4 (0.1,0.8)	0.8 (0.5,1.2)	0.4 (0.1,0.6)	0.7 (0.6,0.8)	0.5 (0.1,1)	0.8 (0.4,1)	0.5 (0.1,0.8)	0.4 (0.25,0.6)	0.1 (0.01,0.2)	0.4 (0.1,0.8)	0.1 (0.05,0.2)	0.5 (0.4,0.8)	0.25 (0.01,1)	1 (0.5,2)
A2.2.3	0.2 (0.1,0.75)	0.7 (0.5,1.4)	0.2 (0.01,0.5)	0.3 (0.01,0.5)	0.3 (0.1,1)	0.5 (0.2,0.8)	0.1 (0.01,1)	0.3 (0.15,0.4)	0.1 (0.01,0.2)	0.1 (0.05,0.3)	0.05 (0.01,0.07)	0.3 (0.4,0.6)	0.1 (0.01,1)	0.8 (0.4,1.5)
A2.2.4	0.1 (0.1,0.5)	0.3 (0.3,1.5)	0.1 (0.01,0.5)	0.1 (0.01,0.2)	0.1 (0.01,1)	0.4 (0.1,0.5)	0.01 (0.01,1)	0.3 (0.15,0.4)	0.1 (0.01,0.2)	0.05 (0.005,0.1)	0.03 (0.01,0.05)	0.1 (0.3,0.4)	0.1 (0.01,1)	0.7 (0.4,1.5)
A2.3.2	0.5 (0.1,0.9)	0.8 (0.5,1.1)	0.4 (0.1,0.6)	0.5 (0.4,0.8)	0.8 (0.2,1)	0.2 (0.01,0.5)	0.3 (0.01,0.5)	0.75 (0.5,0.9)	0.1 (0.01,0.2)	0.4 (0.1,0.8)	0.1 (0.05,0.2)	0.8 (0.6,0.9)	0.1 (0.01,0.5)	0.5 (0.2,0.9)
A2.3.3	0.25 (0.1,0.75)	0.5 (0.3,1.1)	0.2 (0.01,0.5)	0.25 (0.1,0.5)	0.6 (0.2,1)	0.1 (0.01,0.5)	0.1 (0.01,1)	0.75 (0.5,0.9)	0.1 (0.01,0.2)	0.2 (0.05,0.6)	0.05 (0.01,0.07)	0.7 (0.5,0.8)	0.1 (0.01,0.25)	0.4 (0.2,0.8)
A2.3.4	0.1 (0.1,0.5)	0.3 (0.1,1.2)	0.1 (0.01,0.5)	0.1 (0.01,0.2)	0.4 (0.2,1)	0.1 (0.01,0.5)	0.01 (0.01,1)	0.75 (0.5,0.9)	0.1 (0.01,0.2)	0.1 (0.05,0.3)	0.03 (0.01,0.05)	0.6 (0.2,0.7)	0.1 (0.01,0.25)	0.3 (0.1,0.7)
A2.4.2	0.25 (0.1,0.75)	0.6 (0.3,0.9)	0.4 (0.1,0.6)	0.8 (0.5,0.9)	0.6 (0.01,1)	0.2 (0.01,0.5)	0.4 (0.2,0.8)	0.05 (0.01,0.1)	0.4 (0.2,0.6)	0.2 (0.1,0.4)	0.1 (0.05,0.2)	0.9 (0.7,0.95)	0.1 (0.01,0.5)	0.9 (0.5,1.3)
A2.4.3	0.1 (0.1,0.5)	0.5 (0.1,1.1)	0.2 (0.01,0.5)	0.5 (0.3,0.6)	0.4 (0.01,1)	0.1 (0.01,0.5)	0.1 (0.01,0.5)	0.05 (0.01,0.1)	0.2 (0.1,0.5)	0.1 (0.05,0.3)	0.05 (0.01,0.07)	0.8 (0.4,0.9)	0.1 (0.01,0.25)	0.8 (0.4,1.2)
A2.4.4	0.1 (0.1,0.5)	0.3 (0.1,1.4)	0.1 (0.01,0.5)	0.25 (0.01,0.3)	0.2 (0.01,1)	0.1 (0.01,0.5)	0.01 (0.01,0.5)	0.05 (0.01,0.1)	0.1 (0.1,0.2)	0.05 (0.005,0.2)	0.01 (0.01,0.02)	0.7 (0.3,0.8)	0.1 (0.01,0.25)	0.7 (0.3,1.1)
A2.5.2	0.25 (0.1,0.75)	0.8 (0.5,1.1)	0.4 (0.1,0.6)	0.8 (0.5,0.9)	0.8 (0.2,1)	0.3 (0.1,0.8)	0.5 (0.01,1)	0.7 (0.5,0.8)	0.25 (0.1,0.3)	0.2 (0.05,0.5)	0.1 (0.05,0.15)	0.5 (0.45,0.7)	0.1 (0.01,0.5)	0.9 (0.5,1.3)
A2.5.3	0.1 (0.1,0.5)	0.6 (0.4,1.2)	0.2 (0.01,0.01)	0.5 (0.2,0.6)	0.6 (0.2,1)	0.2 (0.1,0.5)	0.2 (0.01,1)	0.5 (0.3,0.6)	0.1 (0.1,0.2)	0.1 (0.001,0.3)	0.02 (0.01,0.04)	0.6 (0.4,0.7)	0.1 (0.01,0.25)	0.8 (0.4,1.2)
A2.5.4	0.1 (0.1,0.5)	0.4 (0.2,1.3)	0.1 (0.01,0.5)	0.4 (0.01,0.5)	0.4 (0.2,1)	0.1 (0.01,0.5)	0.1 (0.01,1)	0.3 (0.1,0.5)	0.1 (0.01,0.2)	0.05 (0.0001,0.1)	0.01 (0.01,0.02)	0.6 (0.3,0.7)	0.1 (0.01,0.25)	0.7 (0.3,1.1)
A2.6.2	0.4 (0.1,0.8)	0.8 (0.4,1)	0.5 (0.1,0.7)	0.5 (0.4,0.6)	0.8 (0.01,1)	0.2 (0.01,0.8)	0.7 (0.5,1)	0.8 (0.6,0.9)	0.4 (0.1,0.5)	0.6 (0.2,0.9)	0.5 (0.25,0.65)	0.5 (0.4,0.7)	0.5 (0.01,1)	0.8 (0.4,1.2)
A2.6.3	0.2 (0.1,0.75)	0.6 (0.3,1.1)	0.2 (0.01,0.5)	0.25 (0.2,0.3)	0.6 (0.01,1)	0.1 (0.01,0.8)	0.6 (0.4,1)	0.6 (0.4,0.6)	0.2 (0.1,0.4)	0.3 (0.1,0.7)	0.2 (0.1,0.3)	0.1 (0.01,0.2)	0.1 (0.01,0.5)	0.75 (0.3,1.1)
A2.6.4	0.1 (0.1,0.5)	0.2 (0.1,1.2)	0.1 (0.01,0.4)	0.1 (0.01,0.2)	0.4 (0.01,1)	0.1 (0.01,0.8)	0.5 (0.1,1)	0.4 (0.2,0.4)	0.1 (0.01,0.2)	0.15 (0.05,0.4)	0.05 (0.01,0.15)	0.1 (0.01,0.2)	0.1 (0.01,0.5)	0.7 (0.3,1.1)

B1

	**1**	**7**	**8**	**23**	**9**	**16**	**19**	**14**	**20**	**24**	**26**	**10**	**18**	**4**
B1.1.1	0.8 (0.2,1.2)	2 (1,2.5)	1 (0.2,2)	1.4 (1,1.5)	1 (0.7,1.3)	1 (0.1,2.5)	0.8 (0.3,1.5)	0.2 (0.05,0.3)	0.5 (0.1,2)	0.75 (0.1,1)	0.6 (0.3,1.5)	1.5 (0.8,2.5)	1.5 (0.5,3)	0.5 (0.3,0.8)
B1.1.2	0.9 (0.2,1.2)	1.7 (1,2)	1 (0.2,2)	1.3 (1,1.5)	1.2 (0.9,1.5)	1 (0.1,2.5)	1 (0.5,1.5)	0.3 (0.05,0.5)	1 (0.05,5)	0.5 (0.1,0.75)	0.3 (0.01,1)	1.3 (1,3)	1.5 (0.5,2)	0.45 (0.2,0.75)
B1.1.3	0.8 (0.2,1.1)	1.3 (0.8,1.8)	1 (0.2,2)	1.3 (1,1.5)	1.5 (1.3,1.7)	1 (0.1,2.5)	1.5 (0.5,2)	0.4 (0.05,0.7)	1 (0.05,5)	0.3 (0.01,0.5)	0.1 (0.01,0.6)	2 (1.1,6)	1.5 (0.5,2)	0.4 (0.15,0.7)
B1.1.4	0.6 (0.1,0.9)	1 (0.5,1.5)	1 (0.2,2)	1.2 (1,1.5)	1.7 (1.5,2)	1 (0.1,2.5)	2 (0.5,4)	0.5 (0.05,0.9)	1 (0.05,5)	0.1 (0.01,0.5)	0.1 (0.01,0.3)	2.5 (1.1,8)	1.5 (0.5,2)	0.35 (0.1,0.65)
B1.2.1	0.9 (0.5,1.5)	2.5 (1.5,3.5)	1.2 (0.2,2)	1.6 (1,1.2)	2 (1.9,2.1)	0.7 (0.5,1.3)	1.5 (0.8,2)	0.5 (0.01,1.5)	0.5 (0.1,2)	1.5 (0.5,2)	0.4 (0.1,1)	2 (1,4)	1.5 (0.5,2)	1.2 (0.6,1.5)
B1.2.2	1.1 (0.6,1.8)	1.8 (1.3,2.5)	1.2 (0.2,2)	1.4 (1.1,1.5)	2.5 (2.3,2.7)	0.8 (0.5,1.5)	1 (0.5,1.5)	0.5 (0.01,1.4)	1 (0.05,5)	1.2 (0.5,1.6)	0.2 (0.05,0.6)	2.5 (1,5)	1 (0.5,1.5)	1 (0.5,1.5)
B1.2.3	1 (0.5,1.5)	1.5 (1,2)	1.2 (0.2,2)	1.3 (1.1,1.5)	2.7 (2.5,2.9)	0.9 (0.6,2)	0.5 (0.3,1.5)	0.5 (0.01,1.3)	1 (0.05,5)	0.75 (0.3,1)	0.1 (0.01,0.3)	2.5 (1,6)	0.5 (0.25,1)	0.1 (0.01,0.2)
B1.2.4	0.9 (0.4,1.3)	1 (0.5,1.5)	1.2 (0.2,2)	1.2 (1.1,1.6)	3 (2.75,3.25)	1 (0.6,2.5)	0.2 (0.1,1.5)	0.5 (0.01,1.2)	1 (0.05,5)	0.6 (0.2,0.75)	0.05 (0.01,0.2)	2.5 (1,8)	0.5 (0.25,1)	0.1 (0.01,0.2)
B1.3.1	0.7 (0.2,0.9)	2 (1,3)	0.8 (0.2,2)	1.2 (1,1.2)	4 (3,5)	1.7 (1,3)	2 (0.5,4)	1.5 (1,2)	0.5 (0.1,2)	0.5 (0.01,0.8)	0.6 (0.1,1.5)	1.8 (1,3)	2 (1,4)	0.6 (0.4,0.8)
B1.3.2	0.8 (0.4,1)	1.5 (1,2.5)	0.8 (0.2,2)	1.2 (1,1.4)	4.5 (4,5)	1.5 (0.7,3)	2 (0.5,4)	1.4 (1,3)	1 (0.05,5)	0.4 (0.01,0.7)	0.4 (0.1,0.8)	2.5 (1,5)	1 (0.5,2)	0.4 (0.2,0.6)
B1.3.3	0.6 (0.3,1)	1 (0.75,1.5)	0.8 (0.2,2)	1.2 (1,1.4)	4.75 (4,6)	1.3 (0.5,2.5)	2 (0.5,4)	1.3 (1,4)	1 (0.05,5)	0.2 (0.01,0.5)	0.2 (0.1,0.6)	2.5 (1,6)	0.5 (0.25,1.5)	0.2 (0.01,0.6)
B1.3.4	0.6 (0.3,1)	1 (0.5,1.5)	0.8 (0.2,2)	1.2 (1,1.4)	5 (4,6)	1.3 (0.5,2.5)	2 (0.5,4)	1 (1,5)	1 (0.05,5)	0.1 (0.01,0.2)	0.1 (0.1,0.3)	2.5 (1,8)	0.5 (0.25,1.5)	0.1 (0.01,0.2)
B1.4.1	1 (0.5,1.5)	3 (1,5)	1 (0.2,2)	2 (1,3)	3 (2.5,5)	1.5 (0.7,2.5)	3 (1,6)	1 (0.8,1.2)	0.5 (0.1,2)	1 (0.1,2)	0.4 (0.1,0.8)	1.5 (1,2.5)	2.5 (1,5)	0.4 (0.2,0.6)
B1.4.2	1.2 (0.5,1.6)	3 (1,5)	1 (0.2,2)	1.5 (1,2)	2 (1,4)	1.4 (0.7,2.5)	3 (1,6)	1 (0.6,1.4)	1 (0.05,5)	1 (0.1,2)	0.2 (0.01,0.6)	2 (1,4)	1.5 (1,3)	0.2 (0.1,0.4)
B1.4.3	1.1 (0.5,1.6)	2 (1,3)	1 (0.2,2)	1.3 (1,1.6)	1.5 (1,3)	1.3 (0.6,2.5)	3 (1,6)	1 (0.4,1.6)	1 (0.05,5)	1 (0.1,2)	0.05 (0.01,0.3)	2.5 (1,5)	1 (0.5,2)	0.1 (0.01,0.2)
B1.4.4	1 (0.5,1.5)	1.5 (1,2)	1 (0.2,2)	1 (1,1.2)	1.25 (1,2)	1.3 (0.5,2.5)	3 (1,6)	1 (0.2,1.8)	1 (0.05,5)	1 (0.1,2)	0.1 (0.01,0.2)	2.5 (1,6)	0.5 (0.1,0.9)	0.1 (0.01,0.2)
B1.5.1	1 (0.5,1.5)	1 (0.5,2)	1 (0.2,2)	1.5 (1,1.6)	4 (2,6)	1.5 (0.7,2.5)	3 (1,6)	0.8 (0.3,1.5)	0.5 (0.1,2)	1 (0.1,2)	0.5 (0.1,1.5)	2 (1,6)	1 (0.5,1.5)	0.1 (0.01,0.3)
B1.5.2	0.9 (0.4,1.4)	1.5 (1,2)	1 (0.2,2)	1.3 (1,1.5)	4.5 (4,6)	1.4 (0.7,2.5)	3 (1,6)	0.7 (0.2,1.6)	1 (0.05,5)	1 (0.1,2)	0.4 (0.1,0.9)	2.5 (1,7)	1 (0.5,1)	0.1 (0.01,0.3)
B1.5.3	0.9 (0.4,1.4)	1.5 (1,2.5)	1 (0.2,2)	1.2 (1,1.4)	5 (3,7)	1.3 (0.6,2.5)	3 (1,6)	0.6 (0.1,1.7)	1 (0.05,5)	1 (0.1,2)	0.3 (0.05,0.5)	3 (1,7)	1 (0.5,1)	0.1 (0.01,0.3)
B1.5.4	0.8 (0.3,1.3)	2 (1,3)	1 (0.2,2)	1.1 (1,1.3)	6 (2,8)	1.3 (0.5,2.5)	3 (1,6)	0.5 (0.1,1.7)	1 (0.05,5)	1 (0.1,2)	0.1 (0.01,0.4)	4 (1.5,8)	1 (0.5,1)	0.1 (0.01,0.2)

B1.6.1	0.7 (0.2,1.2)	3 (1,5)	1 (0.2,2)	1.2 (1,2)	10 (5,20)	2 (1,3.5)	0.7 (0.3,3)	2 (1,2.5)	0.5 (0.05,5)	0.5 (0.01,0.75)	0.4 (0.1,1)	0.6 (0.4,1.2)	2 (0.5,3)	0.5 (0.3,0.8)
B1.6.2	0.8 (0.3,1.3)	2 (1,3)	1 (0.2,2)	1.1 (1,1.8)	15 (2,30)	1.7 (0.8,3)	0.7 (0.3,3)	2 (1,2.5)	1 (0.01,10)	0.3 (0.01,0.5)	0.2 (0.05,0.6)	0.8 (0.3,1.4)	1 (0.5,2)	0.45 (0.2,0.75)
B1.6.3	0.8 (0.3,1.3)	2 (1,3)	1 (0.2,2)	1.1 (1,1.3)	20 (2,35)	1.6 (0.8,3)	0.7 (0.3,3)	2 (1,2.5)	1 (0.01,10)	0.1 (0.01,0.2)	0.1 (0.01,0.4)	1 (0.5,2)	1 (0.5,2)	0.4 (0.15,0.7)
B1.6.4	0.7 (0.2,1.2)	1.5 (0.75,3)	1 (0.2,2)	1 (1,1.3)	20 (2,35)	1.5 (0.7,2.5)	0.7 (0.3,3)	2 (1,2.5)	1 (0.01,10)	0.1 (0.01,0.2)	0.1 (0.01,0.3)	2 (1,5)	1 (0.5,2)	0.1 (0.01,0.2)

	**12**	**27**	**28**	**6**	**17**	**15**	**2**	**3**	**11**	**21**	**25**	**5**	**13**	**22**
B1.1.1	1 (0.5,2)	1 (0.5,1.5)		1 (0.8,1.2)	1.5 (1,3)	0.5 (0.3,0.9)	2 (1.5,10)	0.5 (0.3,0.8)	0.5 (0.4,0.6)	2 (0.25,4)	1.5 (1.2,3)	0.6 (0.9,1.1)	1.1 (1,1.6)	1.5 (0.8,2.5)
B1.1.2	1 (0.5,2)	1 (0.5,1.5)		0.8 (0.6,1)	2 (1,4)	0.7 (0.3,1)	2.5 (1.5,10)	0.4 (0.2,0.7)	0.75 (0.6,0.9)	1 (0.5,2)	1.5 (1.2,3)	0.6 (0.8,1)	1.2 (1,1.7)	1.4 (0.7,2.5)
B1.1.3	1.5 (0.5,2.5)	1 (0.5,1.5)		0.6 (0.4,0.6)	3 (1,5)	1 (0.4,1.5)	3.5 (1,10)	0.3 (0.1,0.5)	0.1 (0.1,0.3)	1 (0.5,2)	1.5 (1.2,3)	0.2 (0.2,0.8)	1.3 (1,1.8)	1.3 (0.7,2)
B1.1.4	2 (0.5,4)	1.4 (0.5,2)		0.4 (0.2,0.5)	4 (1,6)	1.2 (0.5,1.5)		0.2 (0.05,0.3)	0.1 (0.1,0.3)	1 (0.5,2)	1.5 (1.2,3)	0.2 (0.15,0.4)	1.4 (1,2)	1.2 (0.6,2)
B1.2.1	0.75 (0.25,2)	2.5 (1.8,4)		0.7 (0.5,0.9)	1.5 (1,3)	2 (0.5,2.5)	2 (1,10)	1.5 (1.2,1.7)	2 (1,3)	2 (0.25,4)	1 (0.5,3)	1.1 (0.7,1.4)	1.1 (0.75,1.2)	2.5 (0.5,3)
B1.2.2	0.75 (0.25,2)	2.5 (1.8,4)		0.5 (0.4,0.6)	2 (1,4)	2 (0.5,2.5)	2.4 (1,10)	1.3 (1,1.4)	3 (2,5)	1 (0.5,2)	1 (0.5,3)	0.9 (0.4,1)	1.1 (0.75,1.2)	2 (0.4,2.5)
B1.2.3	0.5 (0.1,2)	2.5 (1.8,4)		0.3 (0.1,0.4)	3 (1,5)	2 (0.5,2.5)	2.8 (1,10)	1.2 (0.8,1.4)	0.1 (0.1,0.3)	1 (0.5,2)	1 (0.5,3)	0.7 (0.3,1)	1.1 (0.75,1.2)	1.5 (0.3,2)
B1.2.4	0.25 (0.1,2)	2 (1,4)			2.3 (1,5)	2 (0.5,2.5)	3 (1,10)	1 (0.6,1.2)	0.1 (0.1,0.3)	1 (0.5,2)	1 (0.5,3)	0.5 (0.2,0.7)	1.1 (0.75,1.2)	1.2 (0.3,2)
B1.3.1	1 (0.5,2)	3 (2,5)		0.8 (0.5,0.9)	3 (1,6)	2 (0.5,2.5)	1 (0.4,1.6)	2 (1.5,2.2)	3 (1,6)	1 (0.5,2)	1.2 (1,3)	1.3 (0.8,1.5)	1.2 (0.5,1.3)	1.5 (0.8,2.5)
B1.3.2	1 (0.5,2)	3 (2,5)		0.6 (0.4,0.8)	3 (1,6)	2 (0.5,2.5)	1 (0.4,1.6)	1.7 (1.3,2)	3 (1,6)	1 (0.5,2)	1.2 (1,3)	1.2 (0.7,1.4)	1.2 (0.5,1.3)	1.5 (0.8,2.5)
B1.3.3	1.5 (0.5,2.5)	3 (2,5)		0.5 (0.2,0.7)	3 (1,6)	2 (0.5,2.5)	1 (0.4,1.6)	1.4 (1.1,1.7)	3 (1,6)	1 (0.5,2)	1.2 (1,3)	0.9 (0.5,1)	1.2 (0.5,1.3)	1.5 (0.8,2.5)
B1.3.4	2 (0.5,4)	3 (2,5)		0.3 (0.1,0.4)	3 (1,6)	2 (0.5,2.5)	1 (0.4,1.6)	1.2 (1,1.4)	0.1 (0.01,0.2)	1 (0.5,2)	1.2 (1,3)	0.8 (0.3,0.9)	1.2 (0.5,1.3)	1.5 (0.8,2.5)
B1.4.1	1 (0.5,2)	0.8 (0.2,2)		0.9 (0.8,1)	4 (2,10)		3 (1.5,5)	1.5 (1.2,1.8)	5 (3,8)	1 (0.5,2)	1 (0.5,3)	1.5 (1.2,1.6)	1 (0.5,1.5)	0.8 (0.5,2)
B1.4.2	1 (0.5,2)	1.5 (0.8,2.5)		0.7 (0.4,0.8)	5 (2,10)		2.8 (2,4)	1.2 (1,1.4)	5 (3,8)	1 (0.5,2)	1 (0.5,3)	1.2 (0.9,1.4)	1 (0.5,1.5)	0.7 (0.4,1.8)
B1.4.3	1 (0.5,2)	1.5 (0.8,2.5)		0.5 (0.2,0.6)	5 (2,10)		2.2 (1.1,5)	1 (0.8,1.2)	5 (3,8)	1 (0.5,2)	1 (0.5,3)	0.8 (0.6,1.1)	1 (0.5,1.5)	0.6 (0.4,1.7)
B1.4.4	1	2			5		1.5	0.7	5		1	0.7	1	0.6

	(0.5,2)	(0.9,3.5)			(2,10)		(0.8,2)	(0.5,0.9)	(3,8)		(0.5,3)	(0.3,1)	(0.5,1.5)	(0.3,1.5)
B1.5.1	0.75<br>(0.25,2)	4<br>(1.5,6)		0.8<br>(0.6,0.9)	3<br>(1,6)	2.5<br>(1,3)	4<br>(0.01,100)	0.6<br>(0.4,0.8)	4<br>(2,6)	2<br>(0.5,8)	1<br>(0.5,3)	1<br>(0.7,1.3)	1<br>(0.5,1.5)	2.5<br>(1,4)
B1.5.2	0.75<br>(0.25,2)	4<br>(1.5,6)		0.7<br>(0.5,0.8)	3<br>(1,6)	2.5<br>(1,3)	3<br>(0.01,100)	0.5<br>(0.3,0.8)	4<br>(2,6)	1.5<br>(0.5,4)	1<br>(0.5,3)	0.8<br>(0.6,1.1)	1<br>(0.5,1.5)	3<br>(1,5)
B1.5.3	0.5<br>(0.1,2)	3<br>(1,5)		0.5<br>(0.3,0.6)	3<br>(1,6)	2.5<br>(1,3)	2<br>(0.01,100)	0.4<br>(0.2,0.6)	3<br>(2,5)	1.25<br>(0.5,2)	1<br>(0.5,3)	0.7<br>(0.4,0.9)	1<br>(0.5,1.5)	3<br>(1,5)
B1.5.4	0.25<br>(0.1,2)	2<br>(0.8,4)		0.3<br>(0.1,0.5)	3<br>(1,6)	2.5<br>(1,3)	1.5<br>(0.01,100)	0.3<br>(0.1,0.4)	3<br>(2,5)	1<br>(0.5,2)	1<br>(0.5,3)	0.6<br>(0.3,0.8)	1<br>(0.5,1.5)	3<br>(1,5)
B1.6.1	0.8<br>(0.25,2)	0.6<br>(0.2,1.5)		0.8<br>(0.5,0.9)		0.4<br>(0.2,1)	10<br>(2,100)	1.9<br>(1.4,2)	0.5<br>(0.3,0.6)	0.5<br>(0.1,2)	0.8<br>(0.4,1.5)	0.7<br>(0.5,0.8)	1<br>(0.5,1.5)	1<br>(0.8,3)
B1.6.2	0.75<br>(0.25,2)	0.8<br>(0.1,2)		0.7<br>(0.6,0.8)		0.7<br>(0.2,1)	20<br>(1,100)	1.7<br>(1.1,1.9)	1<br>(0.8,1.2)	1<br>(0.5,2)	0.8<br>(0.4,1.5)	0.5<br>(0.4,0.6)	1.2<br>(0.5,1.5)	1.2<br>(1,3.5)
B1.6.3	0.5<br>(0.1,2)	1.5<br>(0.3,2.5)		0.6<br>(0.5,0.7)		0.7<br>(0.2,2)	50<br>(1,100)	1.5<br>(0.7,1.7)	3<br>(2,4)	1<br>(0.5,2)	0.8<br>(0.4,1.5)	0.4<br>(0.2,0.4)	1.2<br>(0.5,1.5)	1.5<br>(1.1,3.5)
B1.6.4	0.25<br>(0.1,2)	1.5<br>(0.5,3)		0.6<br>(0.5,0.7)		1<br>(0.2,2)	100<br>(1,100)	1<br>(0.5,1.2)	5<br>(3,8)	1<br>(0.5,2)	0.8<br>(0.4,1.5)	0.2<br>(0.1,0.2)	1.2<br>(0.5,1.5)	2<br>(1.5,4)

C1

	1	7	8	23	9	16	19	14	20	24	26	10	18	4
C1.1.1	0.9 (0.4,1.2)	1 (0.5,2)	2 (1,10)	1.2 (0.8,1.6)	2 (1,3)	1.8 (0.9,3)	3 (1.5,6)	0.5 (0.3,1.2)	1.5 (0.1,5)	1.5 (0.75,2)	1.4 (0.8,2)	1 (0.2,2.5)	0.5 (0.2,0.8)	1.8 (1.2,2)
C1.1.2	0.8 (0.3,1.1)	0.5 (0.25,1.5)	2 (1,10)	1.1 (0.5,1.6)	2.5 (1.5,3)	1.8 (0.9,3)	3 (1.5,6)	0.5 (0.3,1.2)	1.5 (0.1,5)	1.4 (0.5,2)	1.2 (0.7,1.6)	1.1 (0.3,2.5)	0.2 (0.1,0.5)	1.4 (1,1.8)
C1.1.3	0.8 (0.3,1.1)	0.3 (0.1,0.5)	2 (1,10)	1 (0.5,1.6)	2.75 (1.75,3.25)	1.8 (0.9,3)	3 (1.5,6)	0.5 (0.3,1.2)	1.5 (0.1,5)	1.3 (0.5,2)	1 (0.5,1.5)	1.3 (0.5,3)	0.2 (0.1,0.5)	1.2 (0.8,1.6)
C1.1.4	0.7 (0.2,1)	0.1 (0.01,0.2)	2 (1,10)	1 (0.5,1.6)	3 (1.5,4)	1.8 (0.9,3)	3 (1.5,6)	0.5 (0.3,1.2)	1.5 (0.1,5)	1.2 (0.5,2)	1 (0.5,1.5)	1.5 (0.5,3)	0.1 (0.01,0.2)	1 (0.6,1.4)
C1.2.1	1.1 (0.5,1.6)	2 (1,4)	4 (1,10)	1 (0.8,1.5)	4 (2,6)	2 (1,3.5)	3 (1.5,6)	0.8 (0.6,1.2)	1.5 (0.1,5)	1.5 (0.75,2)	0.7 (0.4,1.5)	1.3 (0.8,2.5)	1.2 (0.5,2)	0.4 (0.2,0.8)
C1.2.2	1 (0.5,1.5)	1.5 (1,4)	4 (1,10)	0.9 (0.5,1)	3 (1.5,4.5)	1.8 (0.9,3)	3 (1.5,6)	0.8 (0.6,1.2)	1.5 (0.1,5)	1.5 (0.5,2)	0.8 (0.3,1.5)	1.3 (0.8,2.5)	1.1 (0.5,1.6)	0.3 (0.1,0.7)
C1.2.3	1 (0.5,1.5)	1.2 (1,2)	4 (1,10)	0.8 (0.5,1)	2.5 (1.5,3.5)	1.7 (0.8,3)	3 (1.5,6)	0.8 (0.6,1.2)	1.5 (0.1,5)	1.5 (0.5,2)	1 (0.5,1.5)	1.3 (0.8,2.5)	1 (0.5,1.5)	0.2 (0.05,0.7)
C1.2.4	0.9 (0.4,1.4)	1 (0.8,2)	4 (1,10)	0.6 (0.4,1)	2 (1.25,2.75)	1.5 (0.8,3)	3 (1.5,6)	0.8 (0.6,1.2)	1.5 (0.1,5)	1.2 (0.5,2)	1 (0.5,1.5)	1.3 (0.8,2.5)	1 (0.5,1.5)	0.1 (0.01,0.6)
C1.3.1	1 (0.5,1.5)	1 (0.5,2)	2 (1,10)	0.8 (0.4,1.2)	0.7 (0.5,0.9)	1.5 (0.8,3)	1.5 (1,2)	0.8 (0.6,1.2)	1.5 (0.1,5)	1.5 (0.75,2)	0.8 (0.5,1.6)	1.5 (0.3,4)	1.5 (1,2)	0.7 (0.3,0.9)
C1.3.2	1 (0.5,1.5)	0.5 (0.25,1.5)	2 (1,10)	0.7 (0.3,1.2)	0.6 (0.4,0.8)	1.5 (0.8,3)	1.5 (1,2)	0.8 (0.6,1.2)	1.5 (0.1,5)	1.6 (0.5,2.1)	0.9 (0.5,1.5)	1.5 (0.3,4)	1.2 (0.5,1.5)	0.6 (0.2,0.8)
C1.3.3	0.9 (0.4,1.4)	0.3 (0.1,0.5)	2 (1,10)	0.6 (0.3,1)	0.5 (0.3,0.7)	1.5 (0.8,3)	1.5 (1,2)	0.8 (0.6,1.2)	1.5 (0.1,5)	1.7 (0.5,2.2)	1 (0.5,1.5)	1.5 (0.3,4)	1.2 (0.5,1.5)	0.5 (0.1,0.7)
C1.3.4	0.8 (0.3,1.3)	0.1 (0.01,0.2)	2 (1,10)	0.4 (0.1,1.2)	0.3 (0.1,0.5)	1.5 (0.8,3)	1.5 (1,2)	0.8 (0.6,1.2)	1.5 (0.1,5)	1.8 (0.5,2.4)	1 (0.5,1.5)	1.5 (0.3,4)	1.2 (0.5,1.5)	0.4 (0.01,0.6)

	12	27	28	6	17	15	2	3	11	21	25	5	13	22
C1.1.1	3 (1,5)	0.8 (0.5,1.3)	1.5 (0.5,3)	0.8 (0.5,1)	2 (1,10)	0.7 (0.2,1.5)	1.5 (1.1,5)	3 (2,4)	10 (5,15)	1 (0.1,10)	0.5 (0.3,2)	1.8 (1.5,2.3)	2 (1.5,3)	2 (1,3)
C1.1.2	2 (1,4)	0.7 (0.4,1)	1.3 (0.5,2)	0.6 (0.4,0.7)	2 (1,10)	0.7 (0.2,1.5)	1.5 (1.1,5)	2.5 (1,3)	10 (5,15)	1 (0.1,10)	0.5 (0.3,2)	1.5 (1.3,1.8)	2 (1.5,3)	2 (1,3)
C1.1.3	1.5 (1,3)	0.5 (0.3,1)	1.2 (0.5,2)	0.2 (0.1,0.4)	2 (1,10)	0.7 (0.2,1.5)	1.5 (1.1,5)	2 (0.5,2.5)	10 (5,15)	1 (0.1,10)	0.5 (0.3,2)	1.2 (1,1.8)	2 (1.5,3)	3 (1.5,4)
C1.1.4	1 (0.75,2.5)	0.3 (0.1,0.7)	1.1 (0.5,2)	0.1 (0.01,0.2)	2 (1,10)	0.7 (0.2,1.5)	1.5 (1.1,5)	1.5 (0.5,2)	10 (5,15)	1 (0.1,10)	0.5 (0.3,2)	1 (0.6,1.1)	2 (1.5,3)	3 (1.5,4)
C1.2.1	1.5 (1,2.5)	0.8 (0.5,1.4)	1 (0.1,3)	1 (0.8,1.2)	2 (1,10)	1 (0.5,1.5)	1.2 (1,2)	2.5 (2,3)	15 (10,20)	1 (0.5,2)	1.2 (0.8,2.5)	0.9 (0.7,1)	1 (0.75,1.25)	0.8 (0.5,1.8)
C1.2.2	1 (0.75,2)	0.7 (0.4,1.2)	1 (0.1,2)	0.8 (0.6,1)	2 (1,10)	1 (0.5,1.5)	1.4 (1.1,2)	2 (1.5,2.5)	15 (10,20)	1 (0.5,2)	1.3 (0.6,3)	0.7 (0.5,0.8)	1 (0.75,1.25)	0.8 (0.5,1.8)
C1.2.3	1 (0.75,2)	0.5 (0.3,1.1)	1 (0.1,2)	0.6 (0.4,0.8)	2 (1,10)	1 (0.5,1.5)	1.5 (1.1,2.2)	1.5 (0.5,2)	10 (5,15)	1 (0.5,2)	1.4 (0.4,3.5)	0.5 (0.2,0.6)	1 (0.75,1.25)	0.8 (0.5,1.8)
C1.2.4	1 (0.75,2)	0.5 (0.2,1)	1 (0.1,2)	0.4 (0.2,0.6)	2 (1,10)	1 (0.5,1.5)	1.6 (1.1,3)	1 (0.25,1.5)	10 (5,15)	1 (0.5,2)	1.5 (0.4,3.5)	0.3 (0.1,0.5)	1 (0.75,1.25)	0.8 (0.5,1.8)
C1.3.1	1 (0.01,2)	1 (0.5,2)	1 (0.1,3)	1 (0.9,1.2)	2 (1,10)	0.5 (0.1,1)	2 (1.5,2.5)	1.5 (1,2)	6 (3,9)	2 (1,8)	1 (0.2,2.5)	1.4 (1.1,1.6)	1.1 (0.5,1.5)	1.5 (1,3)
C1.3.2	1 (0.01,2)	1 (0.5,2)	1 (0.1,2)	0.9 (0.8,1)	2 (1,10)	0.5 (0.1,1)	2.2 (1.8,2.6)	1 (0.5,1.5)	6 (3,9)	1.75 (1,6)	1 (0.2,2.5)	1.2 (0.8,1.4)	1.1 (0.5,1.5)	1.5 (1,3)
C1.3.3	1 (0.01,2)	1 (0.5,2)	1 (0.1,2)	0.6 (0.4,0.8)	2 (1,10)	0.5 (0.1,1)	2.5 (1.2,5)	0.5 (0.2,0.7)	6 (3,9)	1.5 (1,4)	1 (0.2,2.5)	0.8 (0.4,0.9)	1.1 (0.5,1.5)	1.5 (1,3)
C1.3.4	1 (0.01,2)	1.5 (0.8,2.5)	1 (0.1,2)	0.4 (0.2,0.5)	2 (1,10)	0.5 (0.1,1)	3 (1,10)	0.25 (0.1,0.6)	6 (3,9)	1.25 (1,2)	1 (0.2,2.5)	0.7 (0.3,0.8)	1.1 (0.5,1.5)	1.5 (1,3)

D1

	1	7	8	23	9	16	19	14	20	24	26	10	18	4
D1.1.3	0.5 (0.01,0.8)	0.5 (0.25,0.75)	0.3 (0.01,0.8)		0.05 (0.01,0.1)	0.6 (0.3,0.9)	0.2 (0.1,0.5)	0.7 (0.2,1)	0.1 (0.01,0.8)		0.75 (0.5,1)	0.8 (0.3,1)	0.5 (0.2,1)	0.04 (0.01,0.1)
D1.1.2	0.4 (0.01,0.9)	0.35 (0.25,0.5)	0.2 (0.01,0.6)		0.02 (0.01,0.05)	0.4 (0.01,0.9)	0.2 (0.1,0.5)	0.6 (0.5,1)	0.05 (0.01,0.9)		0.6 (0.3,0.8)	0.5 (0.2,0.9)	0.5 (0.2,1)	0.02 (0.01,0.08)
D1.1.1	0.3 (0.01,0.6)	0.2 (0.1,0.25)	0.1 (0.01,0.5)		0.1 (0.01,0.01)	0.2 (0.01,0.7)	0.2 (0.1,0.5)	0.4 (0.3,0.9)	0.025 (0.01,0.9)		0.4 (0.2,0.7)	0.3 (0.1,0.8)	0.5 (0.2,1)	0.01 (0.01,0.08)
D1.2.3	0.6 (0.1,0.9)	0.15 (0.1,0.4)	0.3 (0.01,0.8)		0.5 (0.3,0.7)	0.8 (0.5,0.9)	0.05 (0.01,0.1)	0.3 (0.1,0.5)	0.1 (0.01,0.8)		0.6 (0.3,0.9)	0.7 (0.5,1)	0.25 (0.1,0.5)	0.04 (0.01,0.1)
D1.2.2	0.5 (0.01,0.8)	0.1 (0.05,0.3)	0.2 (0.01,0.6)		0.3 (0.2,0.6)	0.6 (0.3,0.9)	0.05 (0.01,0.1)	0.25 (0.1,0.4)	0.05 (0.01,0.9)		0.4 (0.2,0.7)	0.4 (0.2,0.6)	0.15 (0.05,0.5)	0.02 (0.01,0.1)
D1.2.1	0.5 (0.01,0.8)	0.05 (0.02,0.1)	0.1 (0.01,0.5)		0.25 (0.15,0.3)	0.2 (0.01,0.7)	0.05 (0.01,0.1)	0.2 (0.1,0.3)	0.025 (0.01,0.9)		0.3 (0.1,0.5)	0.1 (0.01,0.5)	0.1 (0.05,0.5)	0.01 (0.01,0.1)
D1.3.3	0.9 (0.3,1.2)	0.8 (0.5,0.9)	0.8 (0.4,1)		1 (0.8,1)	0.7 (0.3,0.9)	0.6 (0.2,0.8)	1 (0.8,1)	0.1 (0.01,0.8)		0.8 (0.5,1)	0.8 (0.6,1)	0.3 (0.1,0.5)	0.15 (0.01,0.2)
D1.3.2	0.8 (0.2,1.1)	0.5 (0.25,0.75)	0.6 (0.3,0.8)		0.9 (0.8,1)	0.5 (0.1,0.9)	0.6 (0.2,0.8)	0.9 (0.7,1)	0.05 (0.01,0.9)		0.7 (0.5,1)	0.6 (0.2,0.9)	0.25 (0.05,0.5)	0.1 (0.01,0.2)
D1.3.1	0.8 (0.2,1.1)	0.4 (0.15,0.6)	0.4 (0.2,0.8)		0.8 (0.7,0.9)	0.3 (0.1,0.7)	0.6 (0.2,0.8)	0.8 (0.6,1)	0.025 (0.01,0.9)		0.6 (0.3,0.9)	0.3 (0.05,0.6)	0.2 (0.05,0.5)	0.05 (0.01,0.2)
D1.4.3	0.8 (0.2,1.1)	0.15 (0.1,0.4)	0.3 (0.01,0.8)		1 (0.8,1)	0.5 (0.1,0.9)	0.4 (0.2,0.8)	0.4 (0.1,0.6)	0.1 (0.01,0.8)		0.5 (0.2,0.8)	0.75 (0.5,1)	0.25 (0.1,0.5)	0.1 (0.01,0.2)
D1.4.2	0.8 (0.2,1.1)	0.1 (0.05,0.3)	0.2 (0.01,0.6)		0.9 (0.8,1)	0.4 (0.1,0.8)	0.4 (0.2,0.8)	0.3 (0.1,0.5)	0.05 (0.01,0.9)		0.4 (0.1,0.6)	0.6 (0.2,0.9)	0.15 (0.05,0.3)	0.1 (0.01,0.2)
D1.4.1	0.7 (0.1,1)	0.05 (0.02,0.1)	0.1 (0.01,0.5)		0.8 (0.7,0.9)	0.3 (0.1,0.8)	0.4 (0.2,0.8)	0.2 (0.1,0.3)	0.025 (0.01,0.9)		0.3 (0.1,0.5)	0.3 (0.05,0.6)	0.1 (0.05,0.3)	0.1 (0.01,0.2)
D1.5.3	0.9 (0.3,1.2)	0.1 (0.05,0.2)	0.3 (0.01,0.8)		1 (0.95,1)	0.6 (0.1,0.9)	0.3 (0.1,0.8)	0.9 (0.5,0.95)	0.1 (0.01,0.8)		0.8 (0.5,1)	0.9 (0.3,1)	0.4 (0.1,0.6)	0.1 (0.01,0.2)
D1.5.2	0.8 (0.2,1.1)	0.1 (0.05,0.2)	0.2 (0.01,0.6)		0.95 (0.9,1)	0.3 (0.01,0.6)	0.3 (0.1,0.8)	0.85 (0.95,0.9)	0.05 (0.01,0.9)		0.7 (0.3,0.9)	0.6 (0.2,1)	0.2 (0.1,0.6)	0.08 (0.01,0.18)
D1.5.1	0.8 (0.2,1.1)	0.05 (0.02,0.1)	0.1 (0.01,0.5)		0.9 (0.8,1)	0.1 (0.01,0.5)	0.3 (0.1,0.8)	0.8 (0.4,0.9)	0.025 (0.01,0.9)		0.5 (0.3,0.9)	0.3 (0.1,0.8)	0.1 (0.1,0.6)	0.06 (0.01,0.16)
D1.6.3	0.6 (0.01,0.9)	0.8 (0.5,0.9)	0.8 (0.4,1)		0.75 (0.5,0.9)	0.4 (0.1,0.9)	0.6 (0.3,0.8)	0.8 (0.5,1)	0.2 (0.05,0.8)		0.7 (0.5,1)	0.7 (0.6,1)	0.7 (0.25,1)	0.25 (0.01,0.5)
D1.6.2	0.5 (0.01,0.8)	0.5 (0.25,0.75)	0.6 (0.3,0.8)		0.6 (0.4,0.8)	0.2 (0.1,0.9)	0.6 (0.3,0.8)	0.8 (0.5,1)	0.1 (0.01,0.9)		0.6 (0.3,0.8)	0.4 (0.2,0.8)	0.7 (0.15,1)	0.15 (0.01,0.3)
D1.6.1	0.4 (0.01,0.7)	0.4 (0.15,0.6)	0.4 (0.2,0.8)		0.5 (0.4,0.6)	0.1 (0.1,0.9)	0.6 (0.3,0.8)	0.7 (0.5,1)	0.5 (0.01,0.9)		0.5 (0.1,0.7)	0.2 (0.01,0.5)	0.6 (0.15,1)	0.05 (0.01,0.15)

	12	27	28	6	17	15	2	3	11	21	25	5	13	22
D1.1.3	0.4<br>(0.01,0.6)	0.4<br>(0.2,0.8)	0.8<br>(0.1,0.9)	0.8<br>(0.5,0.9)	0.5<br>(0.01,1)	0.1<br>(0.01,0.5)	0.8<br>(0.4,1)	0.8<br>(0.6,1)	0.8<br>(0.6,0.9)	0.1<br>(0.01,0.5)		0.5<br>(0.3,0.7)	0.9<br>(0.2,1)	0.6<br>(0.3,0.8)
D1.1.2	0.2<br>(0.01,0.4)	0.2<br>(0.1,0.4)	0.7<br>(0.1,0.9)	0.7<br>(0.5,0.8)	0.3<br>(0.01,1)	0.1<br>(0.01,0.5)	0.5<br>(0.2,0.8)	0.9<br>(0.7,1)	0.75<br>(0.55,0.85)	0.1<br>(0.01,0.5)		0.3<br>(0.2,0.5)	0.7<br>(0.2,1)	0.5<br>(0.25,0.7)
D1.1.1	0.1<br>(0.01,0.2)	0.1<br>(0.01,0.3)	0.5<br>(0.1,0.9)	0.5<br>(0.3,0.7)	0.1<br>(0.01,1)	0.1<br>(0.01,0.5)	0.3<br>(0.1,0.5)	0.95<br>(0.8,1)	0.7<br>(0.5,0.6)	0.1<br>(0.01,0.5)		0.2<br>(0.1,0.4)	0.5<br>(0.2,1)	0.4<br>(0.2,0.6)
D1.2.3	0.2<br>(0.01,0.4)	0.3<br>(0.1,0.6)	0.9<br>(0.1,0.9)	0.7<br>(0.5,0.8)	0.3<br>(0.01,1)	0.3<br>(0.3,1)	0.8<br>(0.6,1)	0.6<br>(0.5,1)	0.7<br>(0.6,0.75)	0.5<br>(0.1,0.8)		0.2<br>(0.1,0.25)	0.2<br>(0.01,0.5)	0.5<br>(0.2,0.8)
D1.2.2	0.1<br>(0.01,0.2)	0.2<br>(0.01,0.4)	0.8<br>(0.1,0.9)	0.5<br>(0.3,0.6)	0.2<br>(0.01,1)	0.3<br>(0.3,1)	0.5<br>(0.4,0.9)	0.7<br>(0.6,1)	0.65<br>(0.6,0.7)	0.4<br>(0.1,0.8)		0.2<br>(0.1,0.2)	0.2<br>(0.01,0.5)	0.4<br>(0.3,0.7)
D1.2.1	0.05<br>(0.01,0.1)	0.1<br>(0.01,0.3)	0.7<br>(0.1,0.9)	0.4<br>(0.2,0.5)	0.1<br>(0.01,1)	0.3<br>(0.3,1)	0.3<br>(0.2,0.8)	0.75<br>(0.6,1)	0.6<br>(0.5,0.65)	0.3<br>(0.1,0.8)		0.1<br>(0.05,0.15)	0.2<br>(0.01,0.5)	0.3<br>(0.2,0.6)
D1.3.3	0.6<br>(0.01,0.8)	0.8<br>(0.3,0.9)	0.9<br>(0.1,0.9)	0.9<br>(0.7,1)	0.6<br>(0.01,1)	0.3<br>(0.1,1)	0.7<br>(0.6,0.9)	0.7<br>(0.5,1)	0.9<br>(0.8,0.95)	0.8<br>(0.5,1)		0.9<br>(0.6,1)	0.95<br>(0.8,1)	0.9<br>(0.7,1)
D1.3.2	0.4<br>(0.01,0.6)	0.6<br>(0.2,0.6)	0.7<br>(0.1,0.9)	0.8<br>(0.6,1)	0.4<br>(0.01,1)	0.3<br>(0.1,1)	0.5<br>(0.2,0.7)	0.8<br>(0.6,1)	0.85<br>(0.8,0.9)	0.8<br>(0.5,1)		0.7<br>(0.5,0.8)	0.9<br>(0.7,1)	0.8<br>(0.6,1)
D1.3.1	0.2<br>(0.01,0.4)	0.4<br>(0.1,0.5)	0.5<br>(0.1,0.9)	0.7<br>(0.5,0.8)	0.2<br>(0.01,1)	0.3<br>(0.1,1)	0.2<br>(0.01,0.4)	0.9<br>(0.7,1)	0.8<br>(0.7,0.85)	0.8<br>(0.5,1)		0.4<br>(0.3,0.7)	0.8<br>(0.6,1)	0.7<br>(0.5,0.9)
D1.4.3	0.2<br>(0.01,0.4)	0.8<br>(0.5,0.9)	0.9<br>(0.1,0.9)	0.8<br>(0.6,0.9)	0.5<br>(0.01,1)	0.3<br>(0.1,1)	0.4<br>(0.2,0.7)	0.4<br>(0.2,1)	0.9<br>(0.8,0.95)	0.1<br>(0.01,0.8)		0.8<br>(0.6,1)	0.6<br>(0.1,0.8)	0.7<br>(0.4,0.9)
D1.4.2	0.1<br>(0.01,0.2)	0.7<br>(0.3,0.8)	0.8<br>(0.1,0.9)	0.6<br>(0.4,0.7)	0.3<br>(0.01,0.8)	0.3<br>(0.1,1)	0.3<br>(0.01,0.5)	0.6<br>(0.4,1)	0.85<br>(0.8,0.9)	0.1<br>(0.01,0.8)		0.7<br>(0.4,0.9)	0.6<br>(0.1,0.8)	0.6<br>(0.3,0.8)
D1.4.1	0.05<br>(0.01,0.1)	0.3<br>(0.1,0.6)	0.7<br>(0.1,0.9)	0.5<br>(0.2,0.6)	0.1<br>(0.01,0.8)	0.3<br>(0.1,1)	0.2<br>(0.01,0.5)	0.7<br>(0.5,1)	0.8<br>(0.7,0.85)	0.1<br>(0.01,0.8)		0.6<br>(0.3,0.8)	0.6<br>(0.1,0.8)	0.5<br>(0.2,0.7)
D1.5.3	0.4<br>(0.01,0.6)	0.7<br>(0.3,0.8)	0.8<br>(0.1,0.9)	0.8<br>(0.6,0.9)	0.7<br>(0.1,1)	0.2<br>(0.1,0.7)	0.8<br>(0.3,0.9)	0.8<br>(0.6,1)	0.6<br>(0.55,0.7)	0.9<br>(0.5,1)		0.7<br>(0.5,0.9)	0.9<br>(0.5,1)	0.9<br>(0.8,1)
D1.5.2	0.2<br>(0.01,0.4)	0.6<br>(0.2,0.7)	0.7<br>(0.1,0.9)	0.6<br>(0.4,0.7)	0.5<br>(0.1,1)	0.2<br>(0.1,0.7)	0.7<br>(0.1,0.8)	0.85<br>(0.7,1)	0.55<br>(0.5,0.6)	0.8<br>(0.5,1)		0.5<br>(0.3,0.8)	0.8<br>(0.5,1)	0.8<br>(0.7,1)
D1.5.1	0.1<br>(0.01,0.2)	0.3<br>(0.1,0.5)	0.6<br>(0.1,0.9)	0.5<br>(0.2,0.6)	0.3<br>(0.1,1)	0.2<br>(0.1,0.7)	0.3<br>(0.01,0.7)	0.95<br>(0.8,1)	0.5<br>(0.4,0.55)	0.7<br>(0.5,1)		0.4<br>(0.3,0.7)	0.7<br>(0.5,1)	0.75<br>(0.6,1)
D1.6.3	0.2<br>(0.01,0.4)	0.6<br>(0.3,0.9)	0.9<br>(0.1,0.9)	0.8<br>(0.5,0.9)	0.9<br>(0.5,1)	0.3<br>(0.1,1)	0.9<br>(0.7,1)	0.5<br>(0.4,1)	0.9<br>(0.8,0.95)	0.8<br>(0.5,1)		1<br>(0.7,1)	0.7<br>(0.3,0.8)	0.95<br>(0.8,1)
D1.6.2	0.1<br>(0.01,0.2)	0.5<br>(0.2,0.7)	0.8<br>(0.1,0.9)	0.6<br>(0.4,0.7)	0.8<br>(0.5,1)	0.3<br>(0.1,1)	0.8<br>(0.6,1)	0.6<br>(0.5,1)	0.85<br>(0.75,0.9)	0.6<br>(0.3,0.8)		0.8<br>(0.5,1)	0.6<br>(0.3,0.8)	0.9<br>(0.7,1)
D1.6.1	0.05<br>(0.01,0.1)	0.2<br>(0.1,0.5)	0.7<br>(0.1,0.9)	0.5<br>(0.2,0.6)	0.7<br>(0.5,1)	0.3<br>(0.1,1)	0.8<br>(0.6,1)	0.7<br>(0.6,1)	0.8<br>(0.7,0.85)	0.5<br>(0.2,0.7)		0.7<br>(0.3,0.9)	0.5<br>(0.3,0.8)	0.7<br>(0.6,1)

D2

	1	7	8	23	9	16	19	14	20	24	26	10	18	4
D2.1.3	0.6 (0.1,0.9)	0.75 (0.5,0.9)	0.7 (0.5,0.9)		0.8 (0.6,0.9)	0.5 (0.01,0.9)		0.5 (0.1,0.7)	0.9 (0.7,0.95)		0.8 (0.5,1)	0.5 (0.2,0.9)	0.5 (0.25,0.7)	0.85 (0.7,0.95)
D2.1.2	0.7 (0.1,1)	0.6 (0.25,0.75)	0.7 (0.5,0.9)		0.7 (0.5,0.9)	0.4 (0.01,0.9)		0.4 (0.1,0.6)	0.8 (0.6,0.95)		0.7 (0.3,0.9)	0.4 (0.2,0.6)	0.25 (0.15,0.5)	0.8 (0.65,0.93)
D2.1.1	0.8 (0.1,1.1)	0.4 (0.3,0.5)	0.7 (0.5,0.9)		0.5 (0.4,0.6)	0.3 (0.01,0.9)		0.3 (0.1,0.5)	0.7 (0.3,0.95)		0.5 (0.2,0.8)	0.1 (0.05,0.4)	0.15 (0.01,0.5)	0.75 (0.6,0.91)
D2.2.3	0.8 (0.1,1.1)	0.6 (0.4,0.8)	0.5 (0.2,0.8)		0.7 (0.6,0.8)	0.4 (0.01,0.9)	0.1 (0.05,0.2)	0.5 (0.1,0.8)	0.9 (0.7,0.95)		0.7 (0.3,1)	0.5 (0.2,0.9)	0.25 (0.25,0.7)	0.8 (0.6,0.9)
D2.2.2	0.9 (0.2,1.1)	0.4 (0.15,0.6)	0.5 (0.2,0.8)		0.6 (0.5,0.7)	0.3 (0.01,0.9)	0.1 (0.05,0.2)	0.4 (0.1,0.7)	0.8 (0.6,0.95)		0.6 (0.1,0.9)	0.4 (0.2,0.6)	0.2 (0.15,0.5)	0.75 (0.6,0.9)
D2.2.1	0.9 (0.2,1.1)	0.3 (0.15,0.5)	0.5 (0.2,0.8)		0.5 (0.4,0.6)	0.2 (0.01,0.9)	0.1 (0.05,0.2)	0.2 (0.1,0.6)	0.7 (0.3,0.95)		0.4 (0.1,0.8)	0.1 (0.05,0.4)	0.15 (0.15,0.5)	0.7 (0.5,0.9)
D2.3.3	0.8 (0.1,1.1)	0.9 (0.5,0.95)	0.9 (0.7,1)		0.95 (0.85,1)	0.6 (0.01,0.9)		0.9 (0.6,0.95)	0.9 (0.7,0.95)		0.9 (0.5,1)	0.6 (0.3,0.9)	0.6 (0.3,1)	0.9 (0.5,1)
D2.3.2	0.9 (0.2,1.1)	0.8 (0.5,0.9)	0.9 (0.7,1)		0.9 (0.8,0.95)	0.4 (0.01,0.9)		0.8 (0.6,0.8)	0.8 (0.6,0.95)		0.8 (0.4,1)	0.4 (0.2,0.6)	0.5 (0.2,1)	0.88 (0.5,1)
D2.3.1	0.9 (0.2,1.1)	0.75 (0.4,0.85)	0.9 (0.7,1)		0.85 (0.7,0.9)	0.2 (0.01,0.9)		0.7 (0.6,0.6)	0.7 (0.3,0.95)		0.7 (0.3,1)	0.1 (0.05,0.4)	0.4 (0.2,1)	0.85 (0.5,1)
D2.4.3	0.7 (0.1,1)	0.6 (0.4,0.8)	0.7 (0.5,0.9)		0.9 (0.8,1)	0.2 (0.01,0.9)		0.7 (0.1,0.8)	0.9 (0.7,0.95)		0.6 (0.3,0.9)	0.6 (0.3,0.9)	0.3 (0.15,0.8)	0.99 (0.9,1)
D2.4.2	0.8 (0.2,1.1)	0.4 (0.15,0.6)	0.7 (0.5,0.9)		0.85 (0.8,0.9)	0.1 (0.01,0.9)		0.5 (0.1,0.8)	0.8 (0.6,0.95)		0.5 (0.2,0.8)	0.4 (0.2,0.6)	0.15 (0.05,0.8)	0.98 (0.9,1)
D2.4.1	0.8 (0.2,1.1)	0.3 (0.15,0.5)	0.7 (0.5,0.9)		0.8 (0.7,0.9)	0.1 (0.01,0.9)		0.3 (0.1,0.8)	0.7 (0.3,0.95)		0.4 (0.1,0.6)	0.1 (0.05,0.4)	0.1 (0.05,0.5)	0.97 (0.9,1)
D2.5.3	0.8 (0.5,1.1)	0.5 (0.35,0.7)	0.7 (0.5,0.9)		0.8 (0.5,0.95)	0.4 (0.01,0.9)		0.8 (0.3,0.9)	0.9 (0.7,0.95)		0.8 (0.5,1)	0.7 (0.4,0.95)	0.5 (0.05,1)	0.8 (0.6,0.9)
D2.5.2	0.9 (0.4,1.2)	0.45 (0.3,0.6)	0.7 (0.5,0.9)		0.7 (0.5,0.9)	0.2 (0.01,0.9)		0.7 (0.4,0.8)	0.8 (0.6,0.95)		0.75 (0.4,1)	0.4 (0.2,0.6)	0.5 (0.05,1)	0.75 (0.6,0.9)
D2.5.1	0.9 (0.4,1.2)	0.4 (0.25,0.6)	0.7 (0.5,0.9)		0.5 (0.4,0.6)	0.1 (0.01,0.9)		0.6 (0.5,0.7)	0.7 (0.3,0.95)		0.7 (0.3,0.95)	0.2 (0.05,0.4)	0.5 (0.05,1)	0.7 (0.6,0.9)
D2.6.3	0.6 (0.1,0.9)	0.95 (0.75,0.98)	0.5 (0.2,0.8)		0.95 (0.9,1)	0.6 (0.01,0.9)		0.6 (0.1,0.8)	0.9 (0.7,0.95)		0.9 (0.5,1)	0.4 (0.2,0.6)	0.5 (0.1,0.9)	0.85 (0.5,1)
D2.6.2	0.7 (0.1,1)	0.9 (0.7,0.95)	0.5 (0.2,0.8)		0.9 (0.8,0.95)	0.4 (0.01,0.9)		0.5 (0.1,0.8)	0.8 (0.6,0.95)		0.85 (0.5,1)	0.2 (0.1,0.5)	0.4 (0.1,0.9)	0.83 (0.5,1)
D2.6.1	0.7 (0.1,1)	0.85 (0.6,0.9)	0.5 (0.2,0.8)		0.85 (0.7,0.95)	0.2 (0.01,0.9)		0.4 (0.1,0.8)	0.7 (0.3,0.95)		0.7 (0.5,1)	0.1 (0.05,0.2)	0.5 (0.1,0.9)	0.81 (0.5,1)

	12	27	28	6	17	15	2	3	11	21	25	5	13	22
D2.1.3	0.7 (0.05,0.9)	0.6 (0.2,0.8)	0.7 (0.1,0.9)	0.7 (0.3,0.8)		0.7 (0.5,1)	0.6 (0.5,1)	0.4 (0.2,0.8)	0.9 (0.8,0.95)	0.5 (0.1,0.8)		0.7 (0.3,0.8)	0.6 (0.01,1)	0.4 (0.2,0.7)
D2.1.2	0.5 (0.05,0.8)	0.3 (0.1,0.4)	0.6 (0.1,0.9)	0.6 (0.3,0.7)		0.6 (0.4,1)	0.5 (0.2,0.7)	0.6 (0.4,1)	0.85 (0.7,0.9)	0.5 (0.1,0.8)		0.5 (0.2,0.6)	0.6 (0.01,1)	0.3 (0.1,0.8)
D2.1.1	0.3 (0.05,0.7)	0.2 (0.1,0.5)	0.5 (0.1,0.9)	0.4 (0.2,0.5)		0.5 (0.2,1)	0.3 (0.1,0.6)	0.7 (0.5,1)	0.8 (0.7,0.85)	0.5 (0.1,0.8)		0.2 (0.1,0.4)	0.6 (0.01,1)	0.2 (0.1,0.8)
D2.2.3	0.8 (0.05,0.95)	0.5 (0.3,0.7)	0.6 (0.1,0.9)	0.5 (0.3,0.7)		0.8 (0.4,1)	0.5 (0.3,0.8)	0.3 (0.1,0.5)	0.4 (0.3,0.5)	0.5 (0.1,0.8)		0.6 (0.4,0.8)	0.5 (0.01,0.9)	0.3 (0.2,0.6)
D2.2.2	0.6 (0.05,0.75)	0.3 (0.2,0.5)	0.5 (0.1,0.9)	0.3 (0.2,0.5)		0.8 (0.4,1)	0.3 (0.2,0.6)	0.4 (0.2,0.5)	0.35 (0.3,0.4)	0.5 (0.1,0.8)		0.3 (0.2,0.6)	0.5 (0.01,0.9)	0.2 (0.1,0.5)
D2.2.1	0.4 (0.05,0.6)	0.2 (0.1,0.4)	0.4 (0.1,0.9)	0.3 (0.2,0.5)		0.8 (0.4,1)	0.2 (0.1,0.4)	0.5 (0.3,0.7)	0.35 (0.3,0.4)	0.5 (0.1,0.8)		0.2 (0.1,0.5)	0.5 (0.01,0.9)	0.15 (0.1,0.5)
D2.3.3	0.7 (0.03,0.9)	0.5 (0.3,0.7)	0.8 (0.1,0.9)	0.8 (0.5,0.9)		0.9 (0.5,1)	0.9 (0.8,1)	0.5 (0.3,0.8)	0.95 (0.9,0.95)	0.9 (0.7,1)		0.9 (0.6,0.95)	0.8 (0.5,1)	0.8 (0.5,0.9)
D2.3.2	0.5 (0.05,0.8)	0.3 (0.1,0.5)	0.7 (0.1,0.9)	0.7 (0.4,0.8)		0.9 (0.5,1)	0.8 (0.6,1)	0.6 (0.5,0.8)	0.9 (0.85,0.9)	0.8 (0.6,1)		0.7 (0.5,0.9)	0.8 (0.5,1)	0.7 (0.4,0.8)
D2.3.1	0.3 (0.05,0.7)	0.1 (0.1,0.3)	0.6 (0.1,0.9)	0.6 (0.3,0.7)		0.9 (0.5,1)	0.8 (0.6,1)	0.7 (0.5,0.9)	0.9 (0.8,0.9)	0.7 (0.5,1)		0.6 (0.3,0.9)	0.8 (0.5,1)	0.6 (0.3,0.7)
D2.4.3	0.8 (0.05,0.95)	0.6 (0.2,0.8)	0.8 (0.1,0.9)	0.7 (0.3,0.8)		0.8 (0.5,1)	0.7 (0.6,1)	0.5 (0.3,0.8)	0.9 (0.8,0.95)	0.5 (0.1,0.8)		0.7 (0.5,0.9)	0.7 (0.2,0.9)	0.7 (0.5,0.9)
D2.4.2	0.6 (0.05,0.75)	0.3 (0.1,0.5)	0.7 (0.1,0.9)	0.6 (0.3,0.7)		0.8 (0.5,1)	0.6 (0.5,0.8)	0.6 (0.5,0.8)	0.9 (0.8,0.95)	0.5 (0.1,0.8)		0.4 (0.4,0.7)	0.7 (0.2,0.9)	0.6 (0.4,0.8)
D2.4.1	0.4 (0.05,0.6)	0.2 (0.1,0.3)	0.6 (0.1,0.9)	0.4 (0.2,0.5)		0.8 (0.5,1)	0.5 (0.4,0.8)	0.7 (0.5,0.9)	0.9 (0.8,0.95)	0.5 (0.1,0.8)		0.3 (0.2,0.5)	0.7 (0.2,0.9)	0.5 (0.3,0.7)
D2.5.3	0.7 (0.05,0.9)	0.5 (0.2,0.6)	0.8 (0.1,0.9)	0.8 (0.6,0.9)		0.7 (0.5,1)	0.6 (0.4,0.8)	0.7 (0.5,0.8)	0.6 (0.5,0.65)	0.5 (0.2,0.8)		0.8 (0.6,0.95)	0.7 (0.2,1)	0.9 (0.7,1)
D2.5.2	0.5 (0.05,0.8)	0.4 (0.2,0.5)	0.7 (0.1,0.9)	0.6 (0.4,0.8)		0.7 (0.5,1)	0.6 (0.4,0.8)	0.8 (0.6,0.9)	0.6 (0.5,0.65)	0.5 (0.2,0.8)		0.7 (0.3,0.9)	0.7 (0.2,1)	0.8 (0.6,1)
D2.5.1	0.3 (0.05,0.7)	0.2 (0.1,0.4)	0.6 (0.1,0.9)	0.5 (0.4,0.7)		0.7 (0.5,1)	0.6 (0.4,0.8)	0.9 (0.7,1)	0.5 (0.4,0.55)	0.5 (0.2,0.8)		0.5 (0.2,0.9)	0.7 (0.2,1)	0.7 (0.5,1)
D2.6.3	0.6 (0.05,0.8)	0.4 (0.1,0.6)	0.6 (0.1,0.9)	0.9 (0.7,1)		0.9 (0.7,1)	0.9 (0.8,1)	0.4 (0.1,0.6)	0.9 (0.8,0.95)	0.6 (0.1,0.9)		0.6 (0.5,0.8)	0.5 (0.1,0.8)	0.8 (0.5,0.9)
D2.6.2	0.4 (0.05,0.6)	0.3 (0.1,0.3)	0.5 (0.1,0.9)	0.7 (0.6,0.8)		0.9 (0.7,1)	0.9 (0.8,1)	0.5 (0.2,0.7)	0.9 (0.8,0.95)	0.5 (0.1,0.8)		0.5 (0.3,0.7)	0.5 (0.1,0.8)	0.7 (0.4,0.8)
D2.6.1	0.2 (0.05,0.4)	0.05 (0.1,0.4)	0.4 (0.1,0.9)	0.6 (0.4,0.8)		0.9 (0.7,1)	0.9 (0.8,1)	0.6 (0.3,0.8)	0.85 (0.75,0.9)	0.4 (0.1,0.7)		0.2 (0.1,0.4)	0.5 (0.1,0.8)	0.6 (0.3,0.7)
