# Supplementary material for: Health Opportunity Costs: Assessing the Implications of Uncertainty Using Elicitation Methods with Experts
Source: Med Decis Making. 2020 May 22;40(4):448–59. doi: 10.1177/0272989X20916450 (PMC7509606; doi:10.1177/0272989X20916450)
Supplement: Manuscript_expert_elicitation_HOC_MDM_4_Appendix4_online_supp – Supplemental material for Health Opportunity Costs: Assessing the Implications of Uncertainty Using Elicitation Methods with Experts [file Manuscript_expert_elicitation_HOC_MDM_4_Appendix4_online_supp.docx]

# Appendix 1: Fitting of distributions to experts’ stated beliefs

In the methods section, we specified that the first step undertaken to describe the elicited beliefs was to fit a distribution to each quantity elicited from each individual expert. Quantities range between 0 and +infinity, and the Lognormal distribution was pre-specified in the protocol. Given three summaries were elicited from experts, more than one (two-parameter) distribution reasonably reflects the judgements from experts. We protocoled that to reflect this uncertainty, two alternative distributions would be fitted: one using the lower bound (LB) of the credible interval and the mode, and another using the upper bound (UB) and the mode. A unique distribution for each quantity elicited by each expert was then derived by linear pooling the two distributions equally, i.e. pooling means and variances. A post-hoc sensitivity analyses explored sensitivity of pooled descriptions to the choice of distribution, and used of the Gamma distribution as an alternative to the LogNormal. Further details how the fitting was undertaken are described below.

Using a Lognormal distribution

A Lognormal distribution is a continuous probability distribution describing a random variable whose logarithm is normally distributed. A lognormally distributed random variable X is specified using two parameters, μ and σ, that are, respectively, the mean and standard deviation of the variable’s natural logarithm. The mean of X is exp(μ + σ^2^/2), the median exp(μ) and the mode exp(μ - σ^2^). Percentiles of the CDF can be generated by exp(μ -Φ(percentile)* σ).

The steps undertaken for the fitting were as follows:

1. The mode (M) and the upper bound of the 80% credible interval (UB) were used to evaluate the two parameters of the lognormal distribution. The σ_UB_ was {-Φ(0.9) +SQRT[Φ(0.9)^2^-4*(ln(M)-ln(UB))]}/2. The μ_UB_ was ln(M)+ σ^2^. When using the upper bound and the mode, a closed form solution was always reached.
2. When using the mode (M) and the lower bound of the 80% credible interval (LB), a closed form solution was not always available.
   1. If σ_LB_ could be evaluated, i.e. if {Φ(0.9) +SQRT[Φ(0.9)^2^-4*(ln(M)-ln(LB))]}/2 returned a solution, this value was assumed and μ_LB_ was calculated as in point 1 above.
   2. If σ_LB_ could not be evaluated using the expression in 2.a, then minimum square errors was used to find the distribution parameters that provided the closest solution. This was done using the Solver Add-in of Excel, a simulation based procedure.
3. Once the two solutions were obtained, these were pooled using linear pooling, assuming the two distributions as two groups of the same population. The two distributions were equally weighted, and hence sample size or precision does not appear as weighting. The following procedure was used:
   1. The mean (EXP(μ +σ^2^)) and standard deviation on the natural scale (SQRT((EXP(σ^2^)-1)*EXP(2*μ+σ^2^))) were obtained for each pair.
   2. The pooled distribution was obtained using the following expressions

$${mean}_{pooled}={{(mean}_{LB}+{mean}_{UB})}/2$$

$${{SD}^{2}}_{pooled}={({SD}_{LB}^{2}+{mean}_{LB}^{2}+{SD}_{UB}^{2}+{mean}_{UB}^{2})}/{2-}{mean}_{pooled}^{2}$$

To illustrate the fitting process three examples are shown below.

Table 1.1: Illustration of the fitting of LogNormal distributions to individual experts’ responses. A1 (duration of effects) in circulatory.

|  | Response to elicitation | Fitted to mode and lower bound, A | Fitted to mode and upper bound, B | Distributions A and B pooled |
| --- | --- | --- | --- | --- |
|  | Mode (lower, upper bounds of the 80% credible interval), natural scale | | | |
| Expert 1 | 3 (2,6) | 3.0 (2.0,12.4) | 3.0 (2.1,6.0) | 3.4 (2.3,11.5) |
| Expert 2 | 6 (2,10) | 5.1 (3.4, 17.4) | 6.0 (4.4,10) | 6.2 (4.1,16.3) |
| Expert 3 | 5 (2,10) | 4.4 (2.9, 15.1) | 5.0 (3.5,10.0) | 5.6 (3.8,14.5) |

Quantities A to C. Gamma

A Gamma distribution is a continuous probability distribution that describes continuous random variables with support between 0 and + infinity. A Gamma distributed random variable can be specified using two parameters, α >0 and β>0, that represent, respectively, a shape and a rate parameter. The mean is α*β, the mode is (α -1)* β when α ≥1 and zero otherwise, and the variance is β^2^*α. The CDF is not defined in closed form.

The steps undertaken for the fitting were as follows:

1. The two parameters of the Gamma distribution were initialised assuming a value of 2, and were used to calculate a mode and upper bound of the 80% credible interval. The mode was set to 0 when α <1, otherwise the mode was evaluated at (α -1)* β. The bound of the credible interval was evaluated using the gamma.inv function in Excel. The solver procedure was then run to find the solution to α or β that minimised squared errors. Given the introduction of a discontinuation in the constraints to the solver (i.e. mode is zero when a<1), the Evolutionary Solving method was used within solver.
2. The same procedure was ran but now using the mode (M) and the lower bound of the 80% credible interval (UB).
3. Once the two solutions were obtained, these were pooled using linear pooling. The following procedure was used:
   1. The mean and standard deviation of the distributions were obtained from each set of parameters.
   2. The mean and standard deviation of the pooled distribution was obtained as explained above. The parameters of the Gamma were then evaluated from the mean and standard deviation using: of α =mean^2/sd^2 and β = sd^2/mean.

To illustrate the fitting process three examples are shown below.

Table 1.2: Illustration of the fitting of Gamma distributions to individual experts’ responses. A1 (duration of effects) in circulatory.

|  | Response to elicitation | Fitted to mode and lower bound | Fitted to mode and upper bound | Previous two distributions pooled |
| --- | --- | --- | --- | --- |
|  | Mode (lower, upper bounds of the 80% credible interval), natural scale | | | |
| Expert 1 | 3 (2,6) | 3 [2,5] | 3 [1.8,6] | 3 [1.9,5.5] |
| Expert 2 | 6 (2,10) | 5.5 [2.9,20.1] | 6 [4,10] | 4.9 [2.6,16.3] |
| Expert 3 | 5 (2,10) | 4.7 [2.5,17.3] | 5 [3,10] | 4.3 [2.3,14.3] |
