# Supplementary material for: Health Opportunity Costs: Assessing the Implications of Uncertainty Using Elicitation Methods with Experts
Source: Med Decis Making. 2020 May 22;40(4):448–59. doi: 10.1177/0272989X20916450 (PMC7509606; doi:10.1177/0272989X20916450)
Supplement: Manuscript_expert_elicitation_HOC_MDM_4_Appendix5_online_supp – Supplemental material for Health Opportunity Costs: Assessing the Implications of Uncertainty Using Elicitation Methods with Experts [file Manuscript_expert_elicitation_HOC_MDM_4_Appendix5_online_supp.docx]

# Appendix 2: Sensitivity analyses

*2.1 Face validity, primary analyses*

In each section, participants were asked: “Are you confident the answers you gave to questions reflect your views and uncertainties?” The possibilities for answers were: ‘yes’, ‘not sure’ and ‘no’. The following sensitivity analyses: (A) consider only those that responded ‘yes’ or ‘not sure’, (B) consider only those that responded ‘yes’.

*A. Individuals that responded ‘yes’ or ‘not sure’ to whether they were confident their answers reflected their views and uncertainties*

Table 2.1A: Duration of effects, surrogacy and extrapolation – all clinical experts (only individuals that responded ‘yes’ or ‘not sure’ to whether they were confident their answers reflected their views and uncertainties).

|  |  | year 1 | year 2 | year 3 | year 4 | Total additional duration (yrs)* |
| --- | --- | --- | --- | --- | --- | --- |
|  |  | Mode [Mean] (lower, upper bounds of the 80% credible interval) | | | | |
| Circulatory | mortality effects (vs. year 1) | estimable | 0.5 [1.5] (0.3,2.4) | 0.3 [1.2] (0.2,2.2) | 0.2 [0.9] (0.2,2.1) | 1.7 [11.1] (1.1,5.1) |
|  | surrogacy  (vs. same year) | 0.6 [2.9] (0.4,3.2) | 0.7 [2.9] (0.4,3.2) | 0.6 [2.8] (0.4,3.2) | 0.5 [2.8] (0.3,3.3) | -- |
| Respiratory | mortality effects (vs. year 1) | estimable | 0.3 [1.5] (0.2,2.6) | 0.4 [0.7] (0.3,1.7) | 0.2 [0.7] (0.2,1.8) | 1.1 [9.1] (0.8,5.1) |
|  | surrogacy  (vs. same year) | 0.5 [3.9] (0.4,3.8) | 0.5 [4] (0.4,3.9) | 0.4 [3.4] (0.3,3.7) | 0.3 [3.4] (0.3,3.8) | -- |
| Gastrointestinal | mortality effects (vs. year 1) | estimable | 0.3 [1.7] (0.2,2.7) | 0.2 [1.1] (0.2,2.3) | 0.2 [0.9] (0.1,2.1) | 0.9 [11.4] (0.6,6.1) |
|  | surrogacy  (vs. same year) | 0.7 [3.6] (0.5,3.5) | 0.4 [4.5] (0.3,4.2) | 0.5 [4.3] (0.3,4.1) | 0.5 [4.2] (0.4,4) | -- |
| Neurological | mortality effects (vs. year 1) | estimable | 0.2 [1.3] (0.2,2.5) | 0.1 [0.9] (0.1,2.2) | 0.1 [1] (0.1,2.2) | 0.9 [6]  (0.6,4.3) |
|  | surrogacy  (vs. same year) | 0.7 [4.2] (0.5,3.8) | 0.8 [3.2] (0.5,3.1) | 0.8 [2.8] (0.6,2.9) | 0.6 [3.1] (0.4,3.4) | -- |
| Endocrinology | mortality effects (vs. year 1) | estimable | 0.3 [1.4] (0.2,2.5) | 0.3 [1] (0.2,2.2) | 0.2 [0.6] (0.2,1.7) | 1.3 [9]  (0.9,4.9) |
|  | surrogacy  (vs. same year) | 0.3 [4.7] (0.3,4.4) | 0.4 [6.3] (0.3,5) | 0.3 [5.2] (0.2,4.6) | 0.3 [5.6] (0.2,4.8) | -- |
| Others with mortality | mortality effects (vs. year 1) | estimable | 0.3 [1.8] (0.2,2.8) | 0.2 [1.1] (0.1,2.4) | 0.1 [0.9] (0.1,2.2) | 1.4 [9.6]  (0.9,5) |
|  | surrogacy  (vs. same year) | 0.4 [4.6] (0.3,4.3) | 0.5 [5.3] (0.4,4.4) | 0.6 [6.4] (0.4,4.8) | 0.5 [8.8] (0.4,5.7) | -- |
| Mental Health | Extrapolation (vs. same year) | 0.8 [3.5] (0.5,3.4) | 0.7 [3.4] (0.5,3.3) | 0.8 [3.1] (0.5,3.1) | 0.8 [2.8] (0.5,3) | -- |
| Musculoskeletal |  | 0.8 [3.9] (0.5,3.5) | 0.9 [3.2] (0.6,3.1) | 0.8 [2.9] (0.5,3) | 0.8 [2.6] (0.6,2.8) | -- |
| Others without mortality |  | 0.8 [3.2] (0.5,3.2) | 0.6 [2.9] (0.4,3.2) | 0.9 [2.1] (0.6,2.4) | 0.7 [2.2] (0.5,2.7) | -- |

* beyond the year of increased expenditure

*B. Individuals that responded ‘yes’ to whether they were confident their answers reflected their views and uncertainties*

Table 2.1B: Duration of effects, surrogacy and extrapolation – all clinical experts (only individuals that responded ‘yes’ to whether they were confident their answers reflected their views and uncertainties).

|  |  | year 1 | year 2 | year 3 | year 4 | Total additional duration (yrs)* |
| --- | --- | --- | --- | --- | --- | --- |
|  |  | Mode [Mean] (lower, upper bounds of the 80% credible interval) | | | | |
| Circulatory | mortality effects (vs. year 1) | estimable | 0.5 [1.5] (0.3,2.4) | 0.3 [1.3] (0.2,2.4) | 0.2 [1.0] (0.2,2.2) | 1.7 [8.4] (1.1,4.2) |
|  | surrogacy  (vs. same year) | 0.7 [2.3] (0.5,2.7) | 0.8 [3.4] (0.5,3.3) | 0.6 [3.9] (0.4,3.8) | 0.6 [4.7] (0.4,4.1) | -- |
| Respiratory | mortality effects (vs. year 1) | estimable | 0.3 [1.7] (0.2,2.7) | 0.4 [0.6] (0.3,1.5) | 0.3 [0.4] (0.2,1.3) | 1.0 [7.8]  (0.7,4.8) |
|  | surrogacy  (vs. same year) | 0.6 [6.7] (0.5,4.9) | 0.7 [7.5] (0.5,5.1) | 0.6 [7.4] (0.4,5.1) | 0.6 [8.1] (0.5,5.4) | -- |
| Gastrointestinal | mortality effects (vs. year 1) | estimable | 0.4 [2.0] (0.3,2.9) | 0.2 [1.4] (0.2,2.5) | 0.2 [1.2] (0.2,2.4) | 0.7 [12.2] (0.5,6.4) |
|  | surrogacy  (vs. same year) | 0.9 [6.3] (0.6,4.5) | 0.7 [10.0] (0.5,5.9) | 0.7 [8.9] (0.5,5.5) | 0.6 [7.2] (0.5,5.0) | -- |
| Neurological | mortality effects (vs. year 1) | estimable | 0.2 [1.7] (0.2,2.8) | 0.1 [1.1] (0.1,2.3) | 0.1 [1.0] (0.1,2.3) | 0.8 [5.6] (0.5,4.3) |
|  | surrogacy  (vs. same year) | 0.8 [7.1] (0.6,4.8) | 1.2 [4.0] (0.8,3.1) | 1.1 [4.5] (0.7,3.4) | 1.0 [6.4] (0.7,4.4) | -- |
| Endocrinology | mortality effects (vs. year 1) | estimable | 0.4 [1.9] (0.3,2.8) | 0.3 [1.3] (0.2,2.4) | 0.3 [0.7] (0.2,1.9) | 1.5 [7.8]  (1.0,4.3) |
|  | surrogacy  (vs. same year) | 1.2 [3.9] (0.8,3.0) | 0.7 [10.1] (0.5,5.9) | 1.2 [5.1] (0.8,3.7) | 1.1 [5.7] (0.7,4) | -- |
| Others with mortality | mortality effects (vs. year 1) | estimable | 0.4 [2.5] (0.3,3.2) | 0.2 [1.5] (0.2,2.6) | 0.1 [1.1] (0.1,2.4) | 1.2 [8.4] (0.8,4.8) |
|  | surrogacy  (vs. same year) | 0.9 [5.5] (0.6,4.1) | 1.2 [8.0] (0.8,4.7) | 1.2 [9.1] (0.8,5.0) | 1.3 [9.8] (0.9,5.2) | -- |
| Mental Health | Extrapolation (vs. same year) | 0.8 [4.0] (0.5,3.6) | 0.8 [3.9] (0.5,3.6) | 0.8 [3.6] (0.5,3.4) | 0.8 [3.1] (0.5,3.1) |  |
| Musculoskeletal |  | 1.0 [4.4] (0.7,3.5) | 1.0 [3.5] (0.7,3.1) | 1.0 [3.1] (0.6,2.9) | 0.9 [2.9] (0.6,2.8) |  |
| Others without mortality |  | 0.8 [3.7] (0.5,3.5) | 0.6 [3.5] (0.4,3.5) | 0.9 [2.3] (0.6,2.5) | 0.8 [2.3] (0.5,2.6) |  |

* beyond the year of increased expenditure

*2.2 Exploration of heterogeneity, primary analysis*

Table 2.2: Duration of effects, surrogacy and extrapolation -- clinical experts from specific clinical area pooled.

|  |  | year 1 | year 2 | year 3 | year 4 | Total additional duration (yrs)* |
| --- | --- | --- | --- | --- | --- | --- |
|  |  | Mode [Mean] (lower, upper bounds of the 80% credible interval) | | | | |
| Circulatory | mortality effects (vs. year 1) | estimable | 0.6 [2.3] (0.4,2.8) | 0.5 [2.3] (0.3,3) | 0.4 [2] (0.3,2.9) | 2.1 [12.8]  (1.5,5) |
|  | surrogacy  (vs. same year) | 0.9 [2.6] (0.6,2.7) | 0.8 [3.3] (0.5,3.2) | 0.7 [4.5] (0.5,4) | 0.6 [5.3] (0.5,4.3) | -- |
| Respiratory | mortality effects (vs. year 1) | estimable | 0.9 [1.2] (0.6,1.6) | 0.7 [0.8] (0.5,1.4) | 0.6 [0.6] (0.4,1.4) | 1.2 [4]  (0.8,3.1) |
|  | surrogacy  (vs. same year) | 1 [2.4] (0.7,2.4) | 1 [1.5] (0.7,1.8) | 0.9 [1.5] (0.6,1.9) | 0.8 [1.3] (0.6,1.9) | -- |
| Gastrointestinal | mortality effects (vs. year 1) | estimable | 0.7 [1] (0.4,1.7) | 0.6 [1] (0.4,1.8) | 0.5 [0.8] (0.3,1.7) | 1.6 [13.1] (1.1,5.7) |
|  | surrogacy  (vs. same year) | 0.7 [1.6] (0.5,2.2) | 0.8 [2.6] (0.5,2.8) | 0.7 [3.2] (0.5,3.3) | 0.6 [5.9] (0.5,4.6) | -- |
| Neurological | mortality effects (vs. year 1) | estimable | 0.7 [6.9] (0.5,4.9) | 0.6 [5.5] (0.5,4.4) | 0.6 [5.9] (0.5,4.6) | 2.3 [20.9] (1.5,6.6) |
|  | surrogacy  (vs. same year) | 0.9 [10.4] (0.6,5.7) | 0.8 [1.9] (0.5,2.4) | 0.7 [1.7] (0.4,2.3) | 0.6 [3.6] (0.4,3.6) | -- |
| Endocrinology | mortality effects (vs. year 1) | estimable | 0.9 [1.3] (0.6,1.8) | 0.8 [1.4] (0.5,2) | 0.3 [0.6] (0.2,1.6) | 1.3 [4.8]  (0.8,3.4) |
|  | surrogacy  (vs. same year) | 0.8 [2] (0.5,2.4) | 0.8 [2.2] (0.5,2.6) | 0.8 [2.3] (0.5,2.6) | 0.8 [2.8] (0.5,2.9) | -- |
| Others with mortality | mortality effects (vs. year 1) | -- | -- | -- | -- | -- |
|  | surrogacy  (vs. same year) | -- | -- | -- | -- | -- |
|  |  |  |  |  |  |  |
| Mental Health | Extrapolation (vs. same year) | 1.1 [2.9] (0.8,2.6) | 1.1 [2.3] (0.7,2.3) | 0.9 [2.1] (0.6,2.3) | 0.7 [2.4] (0.5,2.8) | -- |
| Musculoskeletal |  | 1.8 [6.5] (1.3,3.4) | 1.6 [4.9] (1.1,3) | 1.5 [4.1] (1,2.8) | 1.3 [3.3] (0.9,2.6) | -- |
| Others without mortality |  | -- | -- | -- | -- | -- |

* beyond the year of increased expenditure

Figure 2.1: Illustration of individual experts’ fitted distributions and the pooled distributions–clinical experts


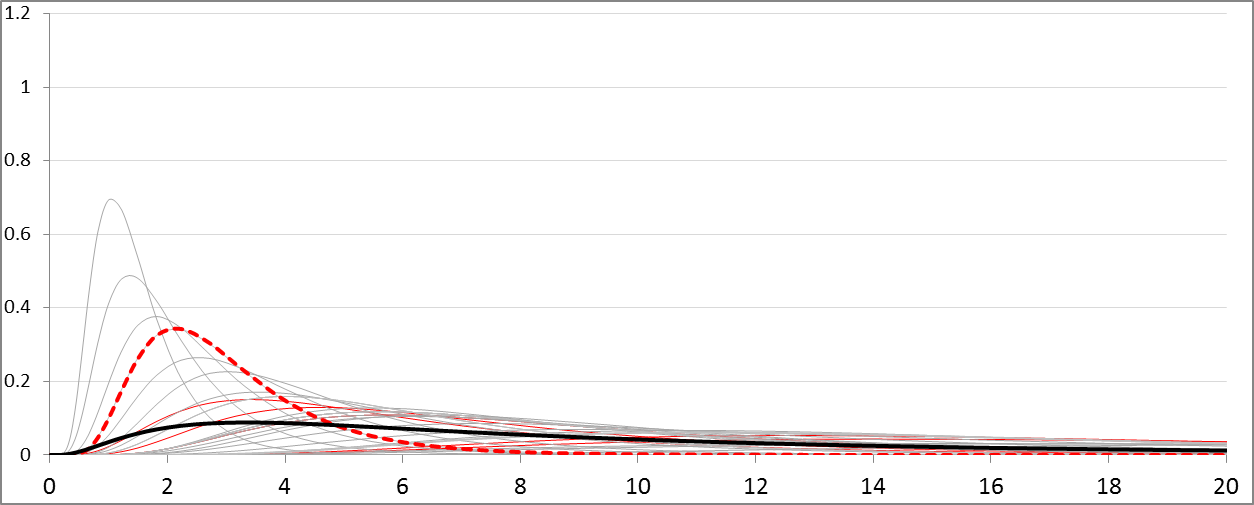


A1 (duration of effects): circulatory

**
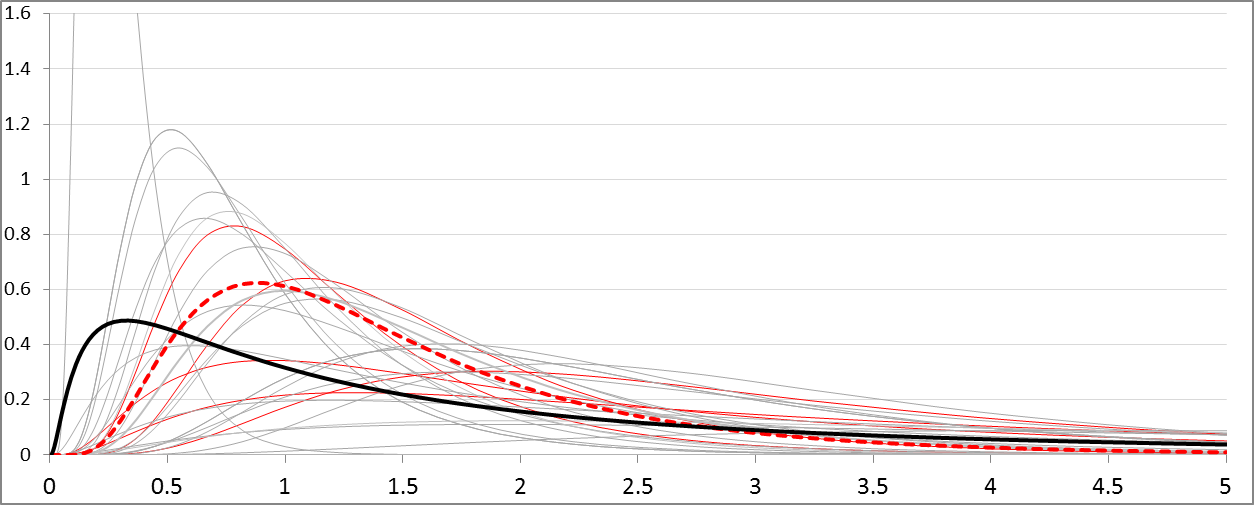
**

B1 (surrogacy), year 1: circulatory

Full red lines: clinical specialists individually; full grey lines: other individual experts; full black line: all clinical experts pooled; red dashed line: clinical specialists pooled

*2.3 Exploring heterogeneity, secondary analysis*

| Group | Description of group | N |
| --- | --- | --- |
| G1 | I have no involvement in policy | 0 |
| G2 | Governmental Bodies (DH, NHSE, PHE) | 15 |
| G3 | Non Departmental Public Bodies and Independent Departmental Expert Committees (NICE, JCVI) | 5 |
| G4 | Industry-related bodies (ABPI) | 2 |
| G5 | Patient representative organisations | 2 |
| G6 | None of the above and indicated other | 1 |

Table 2.3: Duration of effects, surrogacy and extrapolation – policy experts pooled by type of organisation.

|  |  | year 1 | year 2 | year 3 | year 4 | Total additional duration (yrs)* |
| --- | --- | --- | --- | --- | --- | --- |
|  |  | Mode [Mean] (lower, upper bounds of the 80% credible interval) | | | | |
| Circulatory | mortality effects (vs. year 1) | estimable | G2: 0.5 [1] (0.3,2)  G3: 0.2 [2.3] (0.2,5.3)  G4: 0.9 [1.6] (0.6,3)  G5: 0.3 [0.6] (0.2,1.6) | G2: 0.3 [1] (0.2,2)  G3: 0.3 [1] (0.2,2.1)  G4: 0.7 [1.2] (0.4,2.3)  G5: 0.1 [0.3] (0.1,1) | G2: 0.2 [0.8] (0.1,1.8)  G3: 0.1 [0.7] (0.1,1.5)  G4: 0.5 [1.1] (0.4,2.2)  G5: 0.2 [0.3] (0.2,0.6) | G2: 1.5 [19.2] (1.5,43.6);  G3: 5.2 [9.4] (3.5,17.1);  G4: 4.8 [10.2] (3.2,19.7)  G5: 3.3 [7] (2.2,13.5) |
|  | surrogacy  (vs. same year) | G2: 0.3 [2.8] (0.3,6.4)  G3: 0.2 [4] (0.3,9)  G4: 0.9 [2.3] (0.6,4.8)  G5: 0.5 [0.8] (0.3,1.8) | G2: 1 [2] (0.7,3.8)  G3: 0.2 [3.5] (0.3,8)  G4: 0.7 [2.8] (0.5,6.1)  G5: 0.2 [0.5] (0.1,1.7) | G2: 0.9 [1.9] (0.6,3.6)  G3: 0.4 [4.1] (0.4,9.2)  G4: 1.1 [1.7] (0.7,3)  G5: 0.6 [0.8] (0.4,1.5) | G2: 0.9 [1.8] (0.6,3.5)  G3: 0.5 [3.6] (0.4,8.2)  G4: 1 [1.5] (0.7,2.7)  G5: 0.6 [0.7] (0.4,1.4) | -- |
| Respiratory | mortality effects (vs. year 1) | estimable | G2: 0.4 [0.9] (0.3,1.7);  G3: 0.2 [1.3] (0.2,2.8);  G4: 0.7 [1.1] (0.5,1.9);  G5: 0.5 [0.9] (0.3,2.3) | G2: 0.2 [0.6] (0.2,1.3);  G3: 0.2 [0.4] (0.1,0.9);  G4: 0.4 [0.8] (0.3,1.6);  G5: 0.3 [0.7] (0.2,2) | G2: 0.1 [0.5] (0.1,1.2)  G3: 0.1 [0.3] (0.1,0.5)  G4: 0.2 [0.4] (0.1,0.8)  G5: 0.2 [0.7] (0.1,3.3) | G2: 0.5 [14.5] (0.7,32.2)  G3: 1.2 [7.5] (1,16.8);  G4: 5.1 [7.7] (3.4,13.2);  G5: 1.1 [1.9] (0.7,4.8) |
|  | surrogacy  (vs. same year) | G2: 1.2 [2.3] (0.8,4.3)  G3: 0.3 [2.9] (0.3,6.5)  G4: 0.6 [3.1] (0.5,6.9)  G5: 0.6 [1] (0.4,2.6) | G2: 1.1 [2.2] (0.7,4.1)  G3: 0.5 [3.7] (0.4,8.4)  G4: 0.6 [3.6] (0.5,8)  G5: 0.8 [1] (0.5,2) | G2: 0.9 [2] (0.6,3.9)  G3: 0.4 [3.9] (0.3,8.9)  G4: 0.5 [2.9] (0.4,6.4)  G5: 0.7 [0.9] (0.5,1.7) | G2: 0.3 [2.4] (0.3,5.4)  G3: 0.4 [4.3] (0.4,9.7)  G4: 0.4 [2.2] (0.3,4.8)  G5: 0.5 [0.6] (0.3,1.3) | -- |
| Gastrointestinal | mortality effects (vs. year 1) | estimable | G2: 0.1 [1.4] (0.1,3.1)  G3: 0.3 [3.8] (0.3,8.6)  G4: 0.6 [2.7] (0.5,5.9)  G5: 1.4 [2] (0.9,4.1) | G2: 0.2 [1] (0.1,2.1)  G3: 0.1 [2] (0.1,4.6)  G4: 0.4 [2.1] (0.3,4.7)  G5: 1.6 [3.3] (1.1,9.9) | G2: 0.2 [0.8] (0.2,1.8)  G3: 0.2 [2.2] (0.2,5.1)  G4: 0.6 [1] (0.4,1.9)  G5: 2.1 [5.7] (1.6,20.8) | G2: 1.2 [18.1] (1.3,41)  G3: 3.3 [10.7] (2.3,22.5)  G4: 4.6 [14.1] (3.2,29.3)  G5: 1.7 [7.7] (1.6,36.6) |
|  | surrogacy  (vs. same year) | G2: 0.6 [2.6] (0.5,5.7)  G3: 1.1 [1.9] (0.7,3.5)  G4: 0.6 [5.9] (0.5,13.4)  G5: 0.1 [0.5] (0.1,2.8) | G2: 0.6 [2.7] (0.5,5.8)  G3: 0.5 [2.7] (0.4,5.9)  G4: 0.6 [5.4] (0.5,12.2)  G5: 0.2 [0.5] (0.2,1.6) | G2: 0.6 [2.6] (0.4,5.7)  G3: 0.3 [3.4] (0.3,7.8)  G4: 0.5 [4.6] (0.5,10.4)  G5: 0.7 [0.9] (0.5,1.7) | G2: 0.5 [2.6] (0.4,5.7)  G3: 0.5 [2.5] (0.4,5.7)  G4: 0.4 [3.9] (0.4,8.9)  G5: 0.4 [0.6] (0.3,1.2) | -- |
| Neurological | mortality effects (vs. year 1) | estimable | G2: 0.6 [1] (0.4,1.9)  G3: 0.2 [1.4] (0.2,3.1)  G4: 0.9 [1.4] (0.6,2.4)  G5: 0 [0.7] (0.1,7.4) | G2: 0.3 [0.8] (0.2,1.6)  G3: 0.4 [0.8] (0.3,1.6)  G4: 0.7 [1.3] (0.5,2.4)  G5: 4.7 [6.3] (3.1,12.5) | G2: 0.2 [0.6] (0.1,1.4)  G3: 0.2 [0.8] (0.1,1.7)  G4: 0.7 [1.1] (0.5,1.9)  G5: 7.6 [9.8] (5.1,18.7) | G2: 1.3 [18.9] (1.4,43)  G3: 2.9 [8.4] (2,17.5)  G4: 6.2 [9.6] (4.1,16.6)  G5: 0.4 [6.4] (0.7,57.2) |
|  | surrogacy  (vs. same year) | G2: 1.4 [3] (0.9,5.8)  G3: 1.1 [3] (0.7,6.1)  G4: 1.2 [3.5] (0.8,7.1)  G5: 0.4 [1.1] (0.3,3.7) | G2: 1.3 [2.5] (0.9,4.6)  G3: 0.8 [3.4] (0.6,7.5)  G4: 1 [2.6] (0.7,5.1)  G5: 1.4 [1.7] (0.9,3.2) | G2: 1.1 [2.2] (0.7,4.1)  G3: 0.8 [3.4] (0.6,7.4)  G4: 0.6 [3.1] (0.5,6.8)  G5: 1.2 [1.5] (0.8,2.9) | G2: 1 [2] (0.6,3.9)  G3: 0.8 [3.4] (0.6,7.4)  G4: 0.5 [2.2] (0.3,4.9)  G5: 0.9 [1.2] (0.6,2.3) | -- |
| Endocrinology | mortality effects (vs. year 1) | estimable | G2: 0.1 [1.5] (0.1,3.5)  G3: 0.2 [3.1] (0.2,7)  G4: 0.5 [2.5] (0.4,5.5)  G5: 0.3 [0.6] (0.2,1.8) | G2: 0.1 [1.3] (0.1,3)  G3: 0.3 [0.9] (0.2,1.8)  G4: 0.5 [2.3] (0.4,5)  G5: 0.1 [0.4] (0.1,1.8) | G2: 0 [1.2] (0.1,2.6)  G3: 0.3 [0.9] (0.2,1.9)  G4: 0.5 [0.8] (0.3,1.4)  G5: 0.6 [0.9] (0.4,1.8) | G2: 0.9 [16.7] (1,37.6)  G3: 1.8 [6.8] (1.3,14.6)  G4: 4.1 [6.3] (2.8,10.7)  G5: 1.8 [2.4] (1.2,4.8) |
|  | surrogacy  (vs. same year) | G2: 1 [2.5] (0.7,5)  G3: 1.2 [1.9] (0.8,3.4)  G4: 0.6 [3.6] (0.5,8.1)  G5: 1.1 [1.6] (0.7,3.4) | G2: 1 [2.5] (0.7,4.9)  G3: 0.5 [2.6] (0.4,5.8)  G4: 0.6 [3.6] (0.5,8.1)  G5: 0.8 [1.3] (0.5,3.2) | G2: 1.1 [2.5] (0.7,5)  G3: 0.5 [2.6] (0.4,5.8)  G4: 0.6 [3.6] (0.5,8.1)  G5: 1.4 [2] (0.9,4.1) | G2: 1 [2.5] (0.7,5)  G3: 0.5 [2.9] (0.4,6.4)  G4: 0.5 [3.6] (0.5,8)  G5: 1.8 [2.3] (1.2,4.3) | -- |
| Others with mortality | mortality effects (vs. year 1) | estimable | G2: 0.5 [1.1] (0.3,2)  G3: 0.4 [0.8] (0.3,1.5)  G4: 0.5 [4.8] (0.5,10.8)  G5: 0.7 [1.1] (0.5,2.3) | G2: 0.2 [0.9] (0.2,1.9)  G3: 0.2 [0.6] (0.1,1.2)  G4: 0.9 [1.4] (0.6,2.5)  G5: 0.4 [0.6] (0.3,1.4) | G2: 0.2 [0.8] (0.1,1.7)  G3: 0.1 [0.9] (0.1,2)  G4: 0.6 [1.8] (0.4,3.7)  G5: 0.4 [0.6] (0.2,1.3) | G2: 1.3 [18.7] (1.4,42.4)  G3: 1.7 [4.7] (1.2,9.6)  G4: 5.7 [8.7] (3.9,14.9)  G5: 1.1 [1.7] (0.7,4.1) |
|  | surrogacy  (vs. same year) | G2: 0.7 [2.1] (0.5,4.4)  G3: 0.6 [1.2] (0.4,2.3)  G4: 0.6 [4.8] (0.5,10.9)  G5: 0.2 [0.5] (0.1,1.6) | G2: 0.7 [2] (0.5,4.2)  G3: 0.3 [2.3] (0.3,5.1)  G4: 0.5 [5.2] (0.5,11.8)  G5: 0.6 [1.1] (0.4,3.1) | G2: 0.6 [2] (0.4,4.1)  G3: 0.3 [2.3] (0.3,5.2)  G4: 0.5 [2.8] (0.4,6.3)  G5: 0.5 [0.6] (0.3,1.3) | G2: 0.6 [1.9] (0.4,4)  G3: 0.3 [2.3] (0.3,5.3)  G4: 0.5 [1.1] (0.4,2.1)  G5: 0.2 [0.4] (0.2,0.9) | -- |
| Mental Health | Extrapolation (vs. same year) | G2: 1.7 [3.6] (1.1,6.8)  G3: 0.6 [3.5] (0.5,7.8)  G4: 0.9 [5.1] (0.7,11.4)  G5: 0.9 [2.1] (0.6,6.9) | G2: 1.4 [3.5] (0.9,7)  G3: 0.7 [4.1] (0.5,9.1)  G4: 1.2 [2.4] (0.8,4.6)  G5: 0.7 [1.5] (0.5,4.7) | G2: 1.3 [3.1] (0.9,6.2)  G3: 0.5 [3.3] (0.4,7.4)  G4: 0.7 [3.3] (0.5,7.3)  G5: 0.6 [1.1] (0.4,3.1) | G2: 1.1 [3.3] (0.8,6.7)  G3: 0.5 [3.3] (0.4,7.4)  G4: 1 [1.7] (0.7,2.9)  G5: 0.4 [0.7] (0.3,1.8) | -- |
| Musculoskeletal |  | G2: 1.6 [4] (1.1,7.9)  G3: 0.7 [3.1] (0.5,6.7)  G4: 1.5 [4.4] (1.1,9)  G5: 1 [3.2] (0.8,12.7) | G2: 1.3 [3.4] (0.9,6.9)  G3: 1.2 [2.1] (0.8,3.7)  G4: 1.4 [3.6] (1,7.2)  G5: 0.6 [1.7] (0.4,6.5) | G2: 1 [3.1] (0.7,6.4)  G3: 1.2 [2] (0.8,3.6)  G4: 1.2 [2.7] (0.8,5.3)  G5: 0.2 [0.8] (0.2,4.3) | G2: 0.8 [3] (0.6,6.3)  G3: 1.2 [2] (0.8,3.6)  G4: 0.9 [2] (0.6,4)  G5: 0.1 [0.5] (0.1,3.1) | -- |
| Others without mortality |  | G2: 1.1 [3.4] (0.7,7.1)  G3: 0.7 [1.5] (0.5,2.9)  G4: 1 [5.4] (0.8,11.9)  G5: 0.4 [2.4] (0.4,12.6) | G2: 0.9 [2.9] (0.6,6.1)  G3: 0.9 [1.6] (0.6,2.9)  G4: 1.4 [2.6] (0.9,4.9)  G5: 0.1 [0.5] (0.1,3.1) | G2: 0.7 [2.7] (0.5,5.7)  G3: 0.9 [1.6] (0.6,2.9)  G4: 0.8 [3.4] (0.6,7.5)  G5: 0 [0.2] (0,10.4) | G2: 0.6 [2.6] (0.4,5.8)  G3: 0.9 [1.8] (0.6,3.6)  G4: 1.1 [1.7] (0.7,3)  G5: 0 [0.1] (0,7.1) | -- |

* beyond the year of increased expenditure

G2: Governmental Bodies (DH, NHSE, PHE) ; G3: Non Departmental Public Bodies and Independent Departmental Expert Committees (NICE, JCVI); G4: Industry-related bodies (ABPI) ; G5: Patient representative organisations; G6: Other

*2.4 Sensitivity analyses to the choice of distribution, primary analysis*

Table 2.4: Quality of fit illustrated for duration of mortality effects in circulatory disease (question A1, circulatory)

| Exp | Expert's values |  | Lognormal |  |  | Gamma |  |
| --- | --- | --- | --- | --- | --- | --- | --- |
|  |  | Fit LB | fit UB | pool of fits | Fit LB | fit UB | pool of fits |
|  |  | Mode (LB,UB) | Mode (LB,UB) | Mode (LB,UB) [Mean] | Mode (LB,UB) | Mode (LB,UB) | Mode (LB,UB) [Mean] |
| 1 | 3 (2,6) | 3 [2,12.4] | 3 [2.1,6] | 3.4 [2.3,11.5] | 3 [2,5] | 3 [1.8,6] | 3 [1.9,5.5] |
| 2 | 6 (2,10) | 5.1 [3.4,17.4] | 6 [4.4,10] | 6.2 [4.1,16.3] | 5.5 [2.9,20.1] | 6 [4,10] | 4.9 [2.6,16.3] |
| 3 | 5 (2,10) | 4.4 [2.9,15.1] | 5 [3.5,10] | 5.6 [3.8,14.5] | 4.7 [2.5,17.3] | 5 [3,10] | 4.3 [2.3,14.3] |
| 4 | 6 (1,12) | 4.6 [3.1,15.9] | 6 [4.2,12] | 6.5 [4.4,15.8] | 5.1 [2.7,18.4] | 6 [3.6,12] | 5.2 [2.8,15.7] |
| 5 | 7 (3,12) | 6.2 [4.1,21.4] | 7 [5.1,12] | 7.4 [4.9,19.9] | 6.7 [3.6,24.3] | 7 [4.6,12] | 5.8 [3.1,19.6] |
| 6 | 10 (2,12) | 7.9 [5.2,27] | 10 [8.6,12] | 9 [6,24.5] | 8.6 [4.6,31.6] | 10 [8.4,12] | 7.7 [4.1,24.9] |
| 7 | 5 (3,10) | 4.9 [3.2,16.6] | 5 [3.5,10] | 5.8 [3.9,15.6] | 5 [3,10.2] | 5 [3,10] | 5 [3,10.1] |
| 8 | 10 (5,25) | 9.2 [6.1,31.7] | 10 [6.7,25] | 12.6 [8.5,32.2] | 9.9 [5.3,34.8] | 10 [5.6,25] | 9.5 [5.1,30.5] |
| 9 | 3 (2,10) | 3 [2,12.4] | 3 [2,10] | 4.6 [3,13.2] | 3 [2,5] | 3 [1.6,10] | 2.6 [1.4,8.1] |
| 10 | 3 (0,10) | 2.1 [1.4,7.2] | 3 [2,10] | 3.6 [2.4,10] | 2.2 [1.3,4.9] | 3 [1.6,10] | 2 [1.1,8] |
| 11 | 1 (0,2) | 0.7 [0.5,2.4] | 1 [0.7,2] | 1.1 [0.7,2.5] | 0.8 [0.4,2.8] | 1 [0.6,2] | 0.9 [0.5,2.5] |
| 12 | 3 (0.5,5) | 2.3 [1.5,7.9] | 3 [2.2,5] | 3 [2,7.5] | 2.5 [1.4,9.3] | 3 [2,5] | 2.5 [1.3,7.6] |
| 13 | 3 (1,10) | 2.5 [1.7,8.7] | 3 [2,10] | 4 [2.7,10.7] | 2.8 [1.5,10.1] | 3 [1.6,10] | 2.9 [1.5,10] |
| 14 | 20 (10,40) | 18.5 [12.3,63.4] | 20 [14,40] | 22.8 [15.3,60.3] | 19.8 [10.5,70.9] | 20 [12.2,40] | 17.5 [9.3,58.4] |
| 15 | 5 (2,10) | 4.4 [2.9,15.1] | 5 [3.5,10] | 5.6 [3.8,14.5] | 4.7 [2.5,17.3] | 5 [3,10] | 4.3 [2.3,14.3] |
| 16 | 2 (1,5) | 1.8 [1.2,6.3] | 2 [1.3,5] | 2.5 [1.7,6.4] | 2 [1,7.1] | 2 [1.1,5] | 1.9 [1,6.2] |
| 17 | 10 (1,20) | 7.4 [4.9,25.4] | 10 [7,20] | 10.7 [7.3,25.6] | 8.2 [4.4,30] | 10 [6.1,20] | 8.7 [4.7,25.7] |
| 18 | 5 (3,10) | 4.9 [3.2,16.6] | 5 [3.5,10] | 5.8 [3.9,15.6] | 5 [3,10.2] | 5 [3,10] | 5 [3,10.1] |
| 19 | 15 (10,30) | 15 [10,62.1] | 15 [10.5,30] | 17.1 [11.3,57.4] | 15 [10,25.1] | 15 [9.1,30] | 14.9 [9.4,27.8] |
| 20 | 1 (0.5,3) | 0.9 [0.6,3.2] | 1 [0.7,3] | 1.4 [0.9,3.5] | 0.9 [0.5,3.7] | 1 [0.5,3] | 0.9 [0.5,3.4] |
| 21 | 5 (1,10) | 3.9 [2.6,13.5] | 5 [3.5,10] | 5.5 [3.7,13.4] | 4.3 [2.3,15.7] | 5 [3,10] | 4.4 [2.3,13.3] |
| 22 | 15 (10,25) | 15 [10,62.1] | 15 [11,25] | 15.5 [10.3,56] | 15 [10,25.1] | 15 [10,25] | 15 [10,25.1] |
| 23 | 15 (5,20) | 12.7 [8.4,43.6] | 15 [12.1,20] | 14.1 [9.4,39.5] | 13.8 [7.3,50.1] | 15 [11.7,20] | 11.7 [6.2,39.5] |
| 24 | 2 (0.1,2.5) | 1.4 [1,4.9] | 2 [1.7,2.5] | 1.8 [1.2,4.5] | 1.6 [0.9,5.8] | 2 [1.6,2.5] | 1.5 [0.8,4.7] |
| 25 | 5 (2,10) | 4.4 [2.9,15.1] | 5 [3.5,10] | 5.6 [3.8,14.5] | 4.7 [2.5,17.3] | 5 [3,10] | 4.3 [2.3,14.3] |
| 26 | 3 (1,10) | 2.5 [1.7,8.7] | 3 [2,10] | 4 [2.7,10.7] | 2.8 [1.5,10.1] | 3 [1.6,10] | 2.9 [1.5,10] |
| 27 | 10 (3,20) | 8.3 [5.5,28.5] | 10 [7,20] | 11.1 [7.5,27.8] | 9 [4.8,33] | 10 [6.1,20] | 8.7 [4.7,27.6] |
| 28 | 2 (0.1,4) | 1.4 [1,4.9] | 2 [1.4,4] | 2.1 [1.4,5] | 1.6 [0.9,5.7] | 2 [1.2,4] | 1.7 [0.9,5] |

Table 2.5: Duration of effects, surrogacy and extrapolation – all clinical experts, Gamma fit.

|  |  | year 1 | year 2 | year 3 | year 4 | Total additional duration (yrs)* |
| --- | --- | --- | --- | --- | --- | --- |
|  |  | Mode [Mean] (lower, upper bounds of the 80% credible interval) | | | | |
| Circulatory | mortality effects (vs. year 1) | estimable | 0 [1.2] (0.1,1.7) | 0 [0.9]  (0,1.1) | 0 [0.6] (0,0.9) | 8 [9.1]  (4.8,11.7) |
|  | surrogacy  (vs. same year) | 0.7 [1.7] (0.3,3.2) | 0.6 [1.6] (0.4,3.3) | 0.6 [1.6] (0.3,3) | 0.5 [1.6] (0.2,2.7) | -- |
| Respiratory | mortality effects (vs. year 1) | estimable | 0 [1.1] (0,1.2) | 0 [0.7]  (0,0.8) | 0 [0.6] (0,0.7) | 5.3 [6.9]  (1.1,4.2) |
|  | surrogacy  (vs. same year) | 1.2 [2.1] (0.9,5.2) | 1.2 [2.1] (0.8,5) | 0.7 [1.8] (0.3,3.1) | 0.7 [1.8] (0.3,2.9) | -- |
| Gastrointestinal | mortality effects (vs. year 1) | estimable | 0 [1.2] (0,1.2) | 0 [0.9]  (0,0.8) | 0 [0.7] (0,0.5) | 6.4 [7.9]  (2,6.1) |
|  | surrogacy  (vs. same year) | 1.3 [2.1] (1,5.6) | 1.2 [2.1] (0.9,5.2) | 1.1 [2.1] (0.6,4.2) | 0.9 [2] (0.4,3.2) | -- |
| Neurological | mortality effects (vs. year 1) | estimable | 0 [1]  (0,0.8) | 0 [0.6]  (0,0.4) | 0 [0.6] (0,0.3) | 2.8 [4.3]  (0.7,3.4) |
|  | surrogacy  (vs. same year) | 1.5 [2.5] (0.8,4.5) | 1.3 [2.3] (0.6,4.1) | 1.2 [2.3] (0.5,3.7) | 1.1 [2.3] (0.4,3.1) | -- |
| Endocrinology | mortality effects (vs. year 1) | estimable | 0 [1.1] (0,1.2) | 0 [0.9]  (0,0.9) | 0 [0.6] (0,0.7) | 6.2 [7.7]  (1.8,5.8) |
|  | surrogacy  (vs. same year) | 0.8 [3.4] (0.1,1.1) | 0.6 [3.2] (0.1,1) | 0.2 [3]  (0,0.9) | 0.3 [3.1] (0.1,0.9) | -- |
| Others with mortality | mortality effects (vs. year 1) | estimable | 0 [1.2] (0,1.2) | 0 [0.8]  (0,0.7) | 0 [0.6] (0,0.3) | 6.6 [8.1]  (2.1,6.3) |
|  | surrogacy  (vs. same year) | 0.5 [3.3] (0.1,0.9) | 1.1 [3.9] (0.1,1.1) | 2.1 [5.4] (0.1,1) | 3.7 [7.5] (0.1,1) | -- |
|  |  |  |  |  |  |  |
| Mental Health | Extrapolation (vs. same year) | 1.5 [2.8] (0.5,3.3) | 1.3 [2.6] (0.4,2.8) | 1.1 [2.5] (0.3,2.5) | 1.1 [2.6] (0.3,2.5) |  |
| Musculoskeletal |  | 1.8 [3] (0.6,3.6) | 1.6 [2.8] (0.5,3.3) | 1.2 [2.6] (0.3,2.7) | 1.1 [2.6] (0.3,2.5) |  |
| Others without mortality |  | 1.2 [2.3] (0.5,3.6) | 1.1 [2.2] (0.4,3.4) | 0.9 [2.1] (0.4,3.1) | 0.8 [2] (0.3,2.6) |  |

* beyond the year of increased expenditure
