# Supplementary material for: Health Opportunity Costs: Assessing the Implications of Uncertainty Using Elicitation Methods with Experts
Source: Med Decis Making. 2020 May 22;40(4):448–59. doi: 10.1177/0272989X20916450 (PMC7509606; doi:10.1177/0272989X20916450)
Supplement: Manuscript_expert_elicitation_HOC_MDM_4_Appendix6_online_supp – Supplemental material for Health Opportunity Costs: Assessing the Implications of Uncertainty Using Elicitation Methods with Experts [file Manuscript_expert_elicitation_HOC_MDM_4_Appendix6_online_supp.pdf]

Face validity and qualitative responses

Clinical experts, N = 28

		Confident that answers given expressed views and uncertainties?			workshop
ID	Speciality	Section A	B	C	
1	Circulatory	Yes, Not sure	Yes, Not sure	Yes, Not sure	1
7	Circulatory	Yes	Not sure	Not sure	2
8	Circulatory	Not sure	Not sure	Yes	2
23	Circulatory	Yes	Yes	Yes	3
9	Circulatory, neurological, musculoskeletal, other: rehabilitation	Yes	Yes	Yes	2
16	Respiratory	Yes	Yes	Yes	3
19	Respiratory, primary care	Not sure	Not sure	Yes	3
14	GI	Not sure	Not sure	Yes	3
20	GI	Not sure	Yes	Yes	3
24	GI	Not sure	Not sure	missing	3
26	Neurology	Yes	Not sure	missing	5
10	Endocrinology	Not sure	Not sure	Not sure	2
18	Endocrinology	Yes	Not sure	Yes	3
4	Endocrinology, other: general medicine	Not sure	Not sure	Not sure	1
12	Mental health	Yes	Not sure	Not sure	2
27	Mental health	Not sure	Not sure	Not sure	6
28	Mental health	Yes	No	Yes	7
6	Primary care	Yes	Not sure	Yes	2
17	Primary care	No	No	missing	3
15	Primary care, other: pharmacist	Yes	Yes	Yes	3
2	Other: anaesthetics	Yes	Not sure	Yes	1
3	Other: public health, CCG gov body member	Yes	Not sure	Not sure	1
11	Other: public health and geriatric medicine	Yes	Yes	missing	2
21	Other: ophthalmology	Not sure	Not sure	Not sure	3
25	Other: radiology	Not sure	Not sure	Not sure	4
5	No clinical expertise	Yes	Not sure	Yes	2
13	No clinical expertise	Yes	Not sure	Yes	2
22	No clinical expertise	Not sure	No	Not sure	3

Section A

ID	Are you confident the answers you gave reflect your views and uncertainties?	If you responded NOT SURE or NO, please give us some more detail as to why:
1	Yes, Not sure	Yes to cardiovascular, not sure to others
2	Yes	These figures represent my best guess but I am not an expert in any of these areas so they are to some degree an uneducated hunch.
3	Yes	My answers were based on a series of hunches based on best guesses and trying to imagine how they would play out on the basis of funding decisions and population impact of decisions. I am sure my answers reflect my views but ##? with uncertainties. Obviously making gross [sweeping?] assumptions about mortality impact of changes in investment and what investment might actually buy -- obviously.
4	Not sure	Heterogeneity in disease included within ICD areas. Inputs are likely 'clinical' whereas big drivers of mortality may be responsive to 'public health' spending (e.g. smoking, physical activity). I am unclear about the evidence base for manpower (as opposed to 'kit' or therapies) which I perceive to be a major source of costs People in 'my' disease area overwhelmingly die of another ICD area. Cancer + tumours thrown in with other ICD seems difficult. Appreciate that this effort, while associated with uncertainty, is much better than nothing! The ICD itself is a historical artefact that may not reflect a natural taxonomy of disease as we now appreciate pathophysiology.
5	Yes	blank
6	Yes	Blank
7	Yes	Blank
8	Not sure	Some categories have very wide range of conditions and unclear about which are the main cases of mortality.
9	Yes	Blank
10	Not sure	Really difficult to answer for a whole disease area. I would have difficulty even in areas I practice in!
11	Yes	blank
12	Yes	blank
13	Yes	blank
14	Not sure	The range of disease. Lack of knowledge about where the mortality burden exists for the active disease population. Trying to avoid heuristics about long term effects and balance with different disease groups. Filling in boxes in sequences (##? - boredom threshold).
15	Yes	blank
16	Yes	I am a respiratory paediatrician so have been generous in my 80% confidence interval and have not been anchored by clinical bias. Not sure what I have based my estimates on 'tho!
17	No	Too much to aggregate across the last disease area - cancer, neonates, trauma.
18	Yes	The figures previously reflects my views but one full of uncertainty. The endocrinology for e.g T1DM: we start insulin pump then [##?] but its mortality benefits will take time to appear. Similarly with Statins and Hypertensive therapy. Peak benefit of a new type of [##?] stent may be in year2 per individual. Thus I'm unsure how it goes. Furthermore, mortality in neurological CVA will be different to MS in years 1-2-3 because of pt heterogeneity.
19	Not sure	I find it very hard to trade off excess deaths in year 2-4 with residual benefit! If someone has a fatal 2nd MI deferred for year 1 to year 3 [its?] mortality can rise in year 3 vs. residual benefit from treatment in year 3. So statins have delayed benefit with more likely reduction in mortality much later vs. CABG which may shift mortality from year 1 into year 2 or 3.
20	Not sure	The groups of ICD codes are very heterogeneous and the need to average across these in terms of mortality effects is obviously difficult. Therefore I am unsure I have adequately quantified my uncertainty. Probably, on reflection I should be more uncertain.

21	Not sure	These are clearly "impossible" questions. Any certainty is delusional. For [chance ?], would have liked to check the bigger killers in each category. Then estimate impact for each of those. Then go with the [median ?] estimate. Still lots of guess work but would have felt more comfortable.
22	Not sure	They're my best guesses but confounded by (1) ignorance (2) I don't feel able to confidently assign the 80% confidence bounds
23	Yes	blank
24	Not sure	Impacts on other disease areas outside of my speciality area. Also measuring relative impact of public health/ cancer screening programme in cancer domain (relative to other conditions).
25	Not sure	Effect of removal of TX after 1 year depends on (a) whether diseases are changing (b) persistence of Tx effect [note from transcriber: Tx is assumed to represent 'treatment effect'] (c) patient mix within category (d) where Tx is available and where it would be [incurred ?].
26	Yes	There are with wide range of diagnoses under each category when makes it very challenging to [generalise?].
27	Not sure	Not content expert in physical health care
28	Yes	Uncertainty due to not knowing the area

Section B

ID	Are you confident the answers you gave reflect your views and uncertainties?	If you responded NOT SURE or NO, please give us some more detail as to why:
1	Yes, Not sure	Yes to cardiovascular, unsure on the rest
2	Not sure	Very difficult to make statements about a whole PBC, to many variables!
3	Not sure	I am not 100% sure that I've properly grasped the nature of the question, this is obviously added multiple uncertainties.
4	Not sure	As before, ICD categories are heterogeneous with respect to the conditions they include. Some symptoms such as dyspnoea, is fairly specific (cardiovascular, respiratory) others, such as pain, are not. I note that I am most pessimistic about HRQoL gains in my own disease area.
5	Not sure	More difficult to follow the guidance. Would appreciate more examples.
6	Not sure	This is very difficult in areas grouped by disease system rather than mortality burden. Surviving an MI equated with brain stem stroke would feel easier if comparing life threat in different organ system but accept the process makes this impossible.
7	Not sure	A complex health economic concept that is a challenge to extrapolate to group that have many disease processes.
8	Not sure	Difficult due to heterogeneity of conditions in each PBC - those conditions with expected mortality effects are not necessarily the same as those with QoL effects.
9	Yes	blank
10	Not sure	I'm uncertain about my uncertainty!
11	Yes	blank
12	Not sure	Feels very subjective + lack of knowledge of some disease groups confounds issues!
13	Not sure	A more complex thought process to go through than previous question and trying to do this and consider the wide range ICD codes within each PBC was challenging. In particular, the "others" category i.e. considering cancers (the variation within cancers and treatments of) and maternity.
14	Not sure	Again thinking fast becomes an attractive option. Tried to think of balance of effects but again the range of disease mortality vs morbidity is very rich and the distinction between groups, big headlines. I.e. neurology diagnosed disease vs big morbidity ##? diagnoses: spent effect greater earlier. Feels like broad brush strokes, each layer is then relative to the next.
15	Yes	blank

16	Yes	Wildly uncertain
17	No	too much heterogeneity between diseases
18	Not sure	I found this rather challenging to extrapolate Qol burden vs mortality burden while adjusting for life years gained in the groups.
19	Not sure	This is difficult because different diseases and then treatments in a with single ICD code can be expected to have quite different impacts on [##?] of its quality [##?] of life [##?]. Cancers and G.I. problems pull in opposite directions for me (GI Tx has more effects on quality than life expectancy and cancer the opposite). Not clear how to trade off these two (disease prevalence??). Hence, wider confidence intervals throughout!
20	Yes	blank
21	Not sure	Similar to previous answer
22	No	Much harder than A! I think it might be easier to do them in this order: circulatory A then Circulatory B, Respiratory A then Respiratory B etc. The mental juggling required with surrogacy [1 word ?] that change over time is both cognitively taxing and also demands a high degree of knowledge, whether explicit or implicit.
23	Yes	blank
24	Not sure	I cannot offer sufficient expertise regarding impact on endocrine or neurological disorders [int I ?] assumption has been that there is little mortality so effects are proportionally going to be greater for Qol.
25	Not sure	If mortality benefit is a measure of [?] effectiveness then you would expect balance. Some survivors will have extra QoL benefits. Some will have short and long term sequelae of treatment. For some, it is all [gains?] of no [mortality?]. For some, survival is at a cost.
26	Not sure	Very difficult! View reflect knowledge of long term conditions and their effects on QoL
27	Not sure	Not content expert
28	No	blank

Section C

ID	**Are you confident the answers you gave reflect your views and uncertainties?**	**If you responded NOT SURE or NO, please give us some more detail as to why:**
1	Yes, Not sure	This was difficult given the range of conditions in the other areas.
2	Yes	blank
3	Not sure	I'm considerably less certain in areas where multiple PBCs are lumped together.
4	Not sure	Because I doubt any mortality gain for some of these (e.g. musculoskeletal) then relative to something 'with measurable mortality effects' the QALY gain is lower being comprised of HRQoL
5	Yes	Blank
6	Yes	blank
7	Not sure	A challenge to understand the model and extrapolate to unfamiliar disease categories.
8	Yes	blank
9	Yes	blank
10	Not sure	These are not my areas of expertise, apart from some very small areas in musculoskeletal disease. This makes it really difficult to do more than guess about the effectiveness of interventions in these areas, but I think they may prove more effective than many e.g. cancer.
11		blank

12	Not sure	Other categories very difficult to consider as so diverse.
13	Yes	blank
14	Yes	This time I am able to feel the averaging effect and more comfortable here
15	Yes	blank
16	Yes	blank
17		blank
18	Yes	blank
19	Yes	blank
20	Yes	blank
21	Not sure	For mental health it depends then is a judgement on effectiveness of psychological treatment. Close to "uniform prior". For teeth/ear/eyes [are ?] very effective treatments.
22	Not sure	Wow!
23	Yes	blank
24		blank
25	Not sure	variation in disease area and Tx [note from transcriber: Tx is assumed to represent 'treatment effect']
26		Very difficult to pool the conditions to give an overall estimate
27	Not sure	Last group of disorders (skin, LD, etc) very heterogeneous.
28	Yes	blank

Overall feedback

ID	**If you have any comments about any aspects of today please add them here**
1	blank
2	blank
3	It's an incredibly hard even to conceptualise and apply experience and judgement to. Will need very careful explanation in the eventual paper and - more importantly - communication strategy. I think needs very careful set up and making sure the [##?] guide is all ready to go. Wonder if it might be worth sending out some briefing notes in advance.
4	blank
5	blank
6	Excellent insight in to the working of health/cost assessment.
7	An interesting education in some complex health economic concepts. Some pre-reading might have made easier to produce answers (if it did not interfere with the elicitation process?!).
8	blank
9	blank
10	It was a really interesting exercise and a fascinating area. I'd love to be kept informed about how it's going. Thank you for the invitation! I guess it might have been helpful to have more explanation but I realise that you are worried about anchoring and other biases introduced by giving concrete examples.
11	blank
12	Know more concrete examples may bias results but might well help to understand issues more quickly.
13	Good session, well explained given that some of what were asked to do was relatively complex. Did feel tricky considering the range of ICD codes in each category as most included

	ones that ranged trivial to life threatening. Some participants were out of the room when some of the important info on (for example) heuristics was being done. Might be worth asking people to remain present at all times.
14	blank
15	Thinking I may have been in the wrong group. I am a pharmacist working as a commissioner. I have a broad understanding of the disease areas but not the specialist knowledge of the clinicians - sorry!
16	An admirable attempt to capture the unknown but I have anxieties about the methodology here. I would be keen for the findings to be to be validated e.g Qol can be extended by medication use . The post 2008 economic down-turn gives us a nice ecological time frame to see the effects of changing healthcare expenditure on healthcare outcomes.
17	I think its very admirable what you're trying to do this [## ## ?]. However, I do have serious concerns about "rubbish in rubbish out". And also about the spurious apparent accuracy of the point estimates in [##?] early slides you showed us. At what point does inaccurate data become more damaging than not having any data?
18	Thank you v. much. Although this was a subjective and complex exercise, I've left the building a fantastic perspective into what is the price of life.
19	Very interesting but lots of trade-offs in considering how to answer the questions. Maybe [##?] many possible variables in comparison with possible subgroups.
20	blank
21	blank
22	What fun! And how difficult. But hopefully useful.
23	blank
24	blank
25	blank
26	blank
27	blank
28	blank

Policy experts, N = 25

		Confident that answers given expressed views and uncertainties			workshop
ID	Policy Body	Section A	B	C	
1	Governmental Bodies	not sure	yes	yes	1
2	Governmental Bodies	yes	yes	yes	1
3	Governmental Bodies	yes	yes	yes	1
7	Governmental Bodies	not sure	not sure	not sure	1
8	Governmental Bodies	not sure	yes	yes	1
9	Governmental Bodies	not sure	not sure	not sure	1
12	Governmental Bodies	not sure	not sure	yes	2
13	Governmental Bodies	not sure	yes	yes	2
15	Governmental Bodies	not sure	yes	yes	2
16	Governmental Bodies	not sure	not sure	not sure	2
19	Governmental Bodies	yes	yes	yes	2
20	Governmental Bodies	not sure	not sure	not sure	2
21	Governmental Bodies	yes	yes	yes	2
22	Governmental Bodies	yes	yes	yes	2
25	Governmental Bodies	not sure	not sure	missing	2
5	Other public or committees*, Other: lay member of NICE TA committee	yes	not sure	yes	1
6	Other public or committees*	yes	yes	yes	1
10	Other public or committees*	not sure	not sure	yes	1
14	Other public or committees*	yes	yes	yes	2
18	Other public or committees*	yes	yes	yes	2
17	Industry-related bodies	not sure	no	no	2
24	Industry-related bodies	no	no	no	2
11	Patient representative organisations	not sure	not sure	not sure	2
23	Patient representative organisations	not sure	no	not sure	2
4	Other: NHS Clinical Commissioning Group	not sure	not sure	not sure	1

* Non Departmental Public Bodies and Independent Departmental Expert Committees

Section A

ID	Are you confident the answers you gave reflect your views and uncertainties?	If you responded NOT SURE or NO, please give us some more detail as to why:
1	Not sure	Didn't have as much time as I would have liked to think though issues
2	Yes	I have said yes, but to be sincere my views are almost completely derived from the experts views
3	Yes	blank
4	Not sure	Not sure on the cancer & tumors (last section) - the range of potential is so vast given the number of listed disease areas. Survival, morbidity and disease progression will be so variable I am not certain it is possible to reduce this to a one number and range. Whilst more certain about the other named disease areas I am still concerned about variability of services across an entire NHS
5	Yes	I am no expert
6	Yes	blank
7	Not sure	Cancer, tumors, gum, infectious disease etc is a very wide category. Maternal and neonatal interventions can have impacts (positive and negative) decades down the live e.g in neonatology. Partly an issue of heterogeneity
8	Not sure	I was heavily influenced by the views of the clinicians and the disease experts and wasn't able to overlay much of my own opinions of the relative merits of different expenditure
9	Not sure	Difficult to think about basket of prevalent disease in some areas and what interventions affect mortality. Particularly true for neurological disease and the final basket at the bottom
10	Not sure	Hard to imagine mortality effects separate from other effects on disease. QALY may extend beyond years. Really had to think about mortality, and had to get back on A1 (?)answers as a consequence. Can't help thinking (bias) about drugs/pharmaceuticals and their effect! Rather than for example diet or other NHS expenditure
11	Not sure	blank
12	Not sure	No clinical background so relied heavily on expert data with a dose of personal experience (both professional and personal)
13	Not sure	Difference in value for A1 compared to all clinical respondents raises issue of whether I have fully understood the task; I would expect interventions to have knock-on effects, however minute, in all subsequent years
14	Yes	blank
15	Not sure	With no clinical background it is challenging to even have confidence in the uncertainty ranges provided
16	Not sure	Difficult to switch old brain into a rather unusual way of thinking
17	Not sure	Being a non-clinician, I based many of my answers on the range of clinical answers, with a particular focus on the answers from topic experts in that area. However their range of answers by topic experts was so great that I couldn't place much credibility in the answers from the experts. In addition, all experts in their area answered that mortality decreased investment on year after an increased investment. I think this was framed based on initial introductory slides and the experts did not consider how mortality may be higher after year 1
18	Yes	blank
19	Yes	blank
20	Not sure	I am not a clinician. These are gut feelings. The variety of conditions in each group within this exam more difficult as I do know about treatments for some but not (nothing!) the majority

21	Yes	Based on the quick review of clinical expert elicitation
22	Yes	blank
23	Not sure	General lack of knowledge in each therapeutic area, plus many underlying features that you think about when answering questions
24	No	Not a clinical expert on these diseases nor on the duration of benefits provided as a result of treatment. New concepts to consider and then apply without background reading or preparation will affect the value of my answers to this consultation. Limited number of clinical experts have created a reference that has wide variation in answers and therefore of limited value to me
25	Not sure	Time pressure leading to internal inconsistency in my answers. Not sure I'm the best representative from my organisation or that my organisation has much to say on the duration or mortality effects - we focus on the support and oversight of NHS hospital in England, not public health questions

Section B

ID	Are you confident the answers you gave reflect your views and uncertainties?	If you responded NOT SURE or NO, please give us some more detail as to why:
1	Yes	blank
2	Yes	As before my views rely almost solely in those of the clinicians
3	Yes	blank
4	Not sure	Trying to rationalise the relative effect of burden to mortality for a given change of resources was very difficult. Not all interventions can influence patient response in a straight line fashion, at least I don't think so.
5	Not sure	I have insufficient knowledge and expertise to provide informed answers (a trained monkey might provide better answers). Nor I am entirely sure I fully understood the task. I think you would be better to exclude my answers
6	Yes	Explanation: nothing intuitive to me so clinical expert views important. But theses differed so much that they reinforced my view that a 1:1 relationship was reasonable assumption
7	Not sure	Last category too heterogenous
8	Yes	blank
9	Not sure	Really uncertain but can't quite explain why - too many conditions with difficult quality of life mortality ratios. I think most of later years info is irrelevant because impact is mainly short lived
10	Not sure	I can only imagine the effect really in year 1. Hence why I focussed on year 1 providing a different number
11	Not sure	blank
12	Not sure	Difficult to keep focused on concepts. Expert views were extremely varied (including their own certainty in their views)! Harder to apply own experience to this more nuanced question
13	Yes	blank
14	Yes	blank
15	Yes	blank
16	Not sure	As before: complex issues, heterogeneity of diseases, bias as to to which 'circulatory' or 'neurological' diseases appear most immediate to consideration
17	No	Without any clinical knowledge my views and uncertainties are highly uncertain. I based my opinion on the clinical experts, where the ranges were massive. I have doubts that clinicians have a good understanding of the value of spend outside of their clinical expertise area, and the range of

		resources within a clinical expert group was also broad and indicates lack of consistency within topics. Also lack of consistent direction of year on year answers within clinical groups raises concerns.
18	Yes	blank
19	Yes	blank
20	Not sure	Again these are just feelings. Overall I assume that there is a positive health effect through quality of life even if not a good one
21	Yes	Based on the quick review of the clinical expert elicitation
22	Yes	blank
23	No	Lack of knowledge, [completely ?] of disease etc
24	No	I am not a clinician or have sufficient professional experiences to discuss how a disease will progress or respond to treatment. Suggest that these discussion are more directed towards health economist with the relevant clinical insight. I believe there is a bias with the underlying model that an initial investment in Year 1 will be followed by subsequent years of investment which may not reflect tratment requirements (e.g. Hep C)
25	Not sure	blank

Section C

ID	Are you confident the answers you gave reflect your views and uncertainties?	If you responded NOT SURE or NO, please give us some more detail as to why:
1	Yes	blank
2	Yes	As before views guided by the clinicians view
3	Yes	blank
4	Not sure	The creation of my uncertainty is the range of possible disease burden and how this can be projected. As an example trying to consider child or adolescent mental health issues to mental health issues later in life I found the burden/mortality trade-off difficult to fully rationalise. And trying to consider the cost section, things like deafness and vision problems was incredibly challenging
5	Yes	blank
6	Yes	blank
7	Not sure	Final category very heterogeneous
8	Yes	blank
9	Not sure	Much more difficult and lumping together all the other areas too! Could have done it far better vs circulatory or neuro or gastro better
10	Yes	blank
11	Not sure	blank
12	Yes	blank
13	Yes	blank
14	Yes	blank
15	Yes	blank
16	Not sure	As answer for B2
17	No	Direction of clinical expert estimates always decreased. Low numbers of expertise in mental health/musculoskeletal/other categories. Low number of clinical experts over all

18	Yes	blank
19	Yes	blank
20	Not sure	But overall slightly more confident that comparatively speaking I am more comfortable to assume these against the other PBCs (extrapolation)
21	Yes	Again, based on my subjective reading of clinical expert data
22	Yes	blank
23	Not sure	blank
24	No	
25	blank	

Overall feedback

ID	**If you have any comments about any aspects of today please add them here**
1	In the 'other' section there are two very different diseases cancer + traum and injuries. For T+I the recovery may be close to 100% whereas for cancer much lower. Could these be separated? Would it make sense to do individual scores and then re-score after a group discussion in future?
2	blank
3	It was very difficult to understand the questions, although you explained them as well as you could. In thinking about the response there were a lot of aspects to think about and assumptions to make. Would it have helped to have captured some of our thinking in each e.g. what we considered? It would have been helpful to have the most common ICD codes highlighted in each of [two lines] some are more common than others
4	blank
5	I am doubtful of people's ability to estimate these figures. Variation between experts may imply not just uncertainty but unreliability of expertise (or people not understanding the question)
6	blank
7	Task challenging. But many queries about the questions are not apparent that members always were clear about the fundamental question being asked. It could be helpful to give people short pre-reading material
8	blank
9	Very difficult to consider broad range of conditions. Might have been helpful to have commonest causes of death listed per each ICD chapter. Note that the cause of death coding is very biased towards respiratory disease so not convinced that this disease state accurately reflects the interventions in that area
10	On reflection, I would possibly have presented/trainsed everyone on all 4 concepts. And then revisit each before doing the exercise
11	I have profound doubts about the value of any data produced as a result of this exercise. I'm deeply concerned that the outcome of such hasty judgementes or vague estimates is likely to be used to produce a fairly precise number for the value of a displaced QALY that will then be used to deny patients with serious or life-limiting illnesses access to clinically or cost effective treatments, by holding those treatments to a standard of evidence and certainty that has not been applied to existing treatments or indeed to this coming QALY threshold. This is fascinating intellectual exercise but please be aware that this will have life or death consequences for real patients. I'm happy to discuss this further
12	Interesting afternoon! Test-retest would also make for interesting findings. Looking forward to seeing the results
13	blank
14	blank
15	blank
16	These exercises require a thought process that is different. I wonder if pre-warning of a different elicitation exercise would have been helpful in gearing respondents up re cerebral processes

17	Validity of the answers seems challenging due to the low numbers of respondents. Conceptually challenging questionnaire. Long questionnaire. Would have preferred clinical area experts to answer only questions only in their clinical area
18	blank
19	I only differentiated by views on different diseases by following the steer from clinicians evidence. Having a bit more info on the severity and nature of different disease categories would have helped me make more informed judgements
20	I found this very interesting. However, I am uncertain of its value in terms of an objective/evidence based assessment, it does however reflect the value people attach to where benefits might be, which is an interesting societal [?] piece and also of interest
21	blank
22	I don't have expert clinical (or public health) knowledge, I think my 'heuristic' has been to have as little impact on the group average as possible. I have tried to indicate that I am certain that I have a very wide degree of uncertainty in answering all these questions. I remain concerned that this process in giving scientific validity to complete guessmarks, by clinicians and policy-makers alike. I commend the attempt but worry about the interpretation of the results based on almost pefect ignorance! I am not (yet) convinced this is a more robust process than a more considered Delphi consensus approach with presentation of the (low quality) evidence that does exists from experts. But I will follow your work with great interest
23	blank
24	blank
25	blank
